# MALDI SpiralTOF high‐resolution mass spectrometry and Kendrick mass defect analysis applied to the characterization of poly(ethylene‐*co*‐vinyl acetate) copolymers

**DOI:** 10.1002/rcm.7525

**Published:** 2016-03-08

**Authors:** Thierry Fouquet, Sayaka Nakamura, Hiroaki Sato

**Affiliations:** ^1^ National Institute of Advanced Industrial Science and Technology (AIST), Environmental Measurement Technology Group, Environmental Management Research Institute (EMRI)TsukubaIbarakiJapan

## Abstract

**Rationale:**

Poly(ethylene‐*co*‐vinyl acetate) copolymers – usually referred to as EVA – are first class industrial polymers used for applications ranging from padding to photovoltaics as encapsulant for the silicon solar cells. Various techniques have been used for their characterization but the analysis of intact EVA chains using mass spectrometry (MS) has not been reported so far.

**Methods:**

Three copolymers containing 18, 25 and 40 wt% vinyl acetate (VA) have been characterized using an off‐line coupling of size‐exclusion chromatography (SEC) and matrix‐assisted laser desorption/ionization (MALDI) spiral‐time‐of‐flight (TOF) high‐resolution mass spectrometry (HRMS). The representativeness of those results for the entire samples has been checked using ^13^C NMR spectroscopy. Lastly, Kendrick mass defect analysis has been proposed as an alternative and user‐friendly data treatment method.

**Results:**

The shortest chains isolated by SEC fractionation and mass‐analyzed by HRMS have been thoroughly described in terms of end‐groups (found to be hydrogens) and co‐monomeric composition. The VA content was successfully derived from the peak assignments in MS spectra for the EVA 40 wt% and 25 wt% while it tended to be overestimated for the latest EVA 18 wt% (increasing poly(ethylene) character). Similar results have been found using a faster data treatment method relying on the Kendrick mass defect analysis of the MS data.

**Conclusions:**

EVA low molecular weight intact oligomers have been extensively characterized by MS for the first time and the structural features confidently extended to the full sample according to NMR data. The Kendrick mass analysis finally constituted an efficient method for a fast evaluation of their VA content with no need for manual assignment. © 2016 The Authors. *Rapid Communications in Mass Spectrometry* Published by John Wiley & Sons Ltd.

Poly(ethylene‐*co*‐vinyl acetate), also known as ethylene‐vinyl acetate or ethylene vinyl acetate copolymer – further noted EVA for sake of clarity – designates a class of copolymers of prime importance for various industrial and high‐technology applications. EVA is produced by the copolymerization of ethylene (E) and vinyl acetate (VA) monomers, usually under high pressure and high temperature (high‐pressure ethylene polymerization, e.g. autoclave process^1^). Varying the content in VA repeating units will result in different properties (flexibility, crystallinity, melting temperature, softness…) which will further dictate the applications of the copolymer. Low VA content EVA (<10 wt%) are typically used as additives to bitumen.^2^ The major EVA formulation consists nevertheless of 28–33 wt% VA^3^, ^4^, ^5^, ^6^ and finds applications as padding (EVA foam^7^), as part of hot melt adhesives,^8^, ^9^ or as encapsulating material for silicon solar cells, protecting the photovoltaic module from moisture, oxygen and mechanical stress.^3^ Such importance in the industrial landscape thus requires specific analytical techniques and methodologies to be developed to ensure a proper characterization of the materials at every step of the process. In particular, the starting EVA copolymer (before cross‐linking or blending with other polymers) should be thoroughly described in terms of end‐groups, branching and VA content. If several techniques have been proposed in the literature – ranging from NMR^10^, ^11^, ^12^, ^13^, ^14^ to rheological or thermal measurements^15^, ^16^ and Fourier transform infrared spectroscopy (FTIR)^17^ to X‐ray photoelectron spectroscopy (XPS),^18^ the use of mass spectrometry has been mentioned in the form of pyrolysis‐gas chromatography MS fingerprints^19^ or time‐of‐flight secondary ion mass spectrometry (TOF‐SIMS)^20^ only – inducing in both cases a cleavage of the polymeric backbone. To the best of our knowledge and despite its increasing use for the characterization of polymeric materials,^21^, ^22^, ^23^, ^24^, ^25^, ^26^ especially with soft ion sources (electrospray ionization (ESI), matrix‐assisted laser desorption/ionization (MALDI)) to preserve the integrity of the polymeric chains, no report has dealt with the analysis of *intact* EVA copolymers by MS. ESI‐ or MALDI‐MS configurations allow the mass measurement of each individual chain, highlighting the occurrence of different distributions and allowing an accurate description of the samples in terms of repeating units, degrees of polymerization or end‐groups.^27^ The present article proposes a full analytical strategy relying on the use of size‐exclusion chromatography (SEC) and high‐resolution mass spectrometry (HRMS) to gain insight into the microstructure of three commercial EVA samples varying by their VA content (40 wt%, 25 wt% and 18 wt%, further noted EVA40, EVA25 and EVA18) with the aim of deciphering the terminations of the chain and evaluate the VA content from the MS data. The preliminary fractionation of polymeric samples by SEC and the subsequent analysis of the collected fractions by MALDI‐MS – often referred to as 'off‐line coupling'^28^, ^29^ – has been reported by several authors as an efficient analytical technique for polydisperse samples.^30^ Kendrick mass defect (KMD) analysis, recently proposed as a user‐friendly MS data visualization from polymer mass spectra,^31^ will be finally applied for the fast and reliable discrimination of the EVA samples. The three VA contents have been specifically chosen (a) to mimic the 28–33 wt% VA content mainly used in the industry (EVA25)^3^ and (b) as reference (EVA40) sample and limit sample (EVA18), easily and hardly analyzable, respectively.

## Experimental

### Chemicals

Poly(ethylene‐*co*‐vinyl acetate) copolymers (abbreviated as EVA) and {(2*E*)‐2‐methyl‐3‐[4‐(2‐methyl‐2‐propanyl)phenyl]‐2‐propen‐1‐ylidene}malononitrile (known as DCTB) were purchased from Sigma Aldrich (St. Louis, MO, USA). Chloroform (CHCl_3_) used in SEC and tetrahydrofuran (THF) were from Wako Pure Chemical Industries (Osaka, Japan). The poly(methyl methacrylate) standard (*M*_p_ = 1310 g mol^–1^) used for the external calibration of the MALDI mass spectra was purchased from Polymer Laboratories (Church Stretton, UK).

### Size‐exclusion chromatography

Size‐exclusion chromatography (SEC) measurements and fractionations were performed using a HLC8220 GPC system (Tosoh, Tokyo, Japan) equipped with a refractive index detector (RID). Separation was carried out using two TSKgel multipore H_XL_‐M columns (7.8 mm × 300 mm) connected in series following a multipore Hxl guard column. CHCl_3_ was used as the mobile phase at a flow rate of 1 mL min^–1^. EVA samples were dissolved in CHCl_3_ at 2 mg mL^–1^, filtered using Millex syringe‐driven filter units (Merck Millipore, Carrigtwohill, Ireland) and 200 μL of the so‐formed solution were injected for the SEC elutions. Fractionation of the polymeric samples was done by collecting aliquots of 0.5 mL (30 s) into vials directly after the RID, further allowed to air dry for a couple of hours and submitted to mass analysis. Following this re‐concentration procedure, the sensitivity of the mass spectrometer allowed satisfactory mass spectra to be recorded from one single analytical SEC run only. The fractions of interest in the present study were collected after 18'30''–19'00'' (fraction #1), 19'00''–19'30'' (fraction #2) and 19'30''–20'00'' (fraction #3) of elution. Fractions of higher molecular weights were also collected (16'30''–18'30'') and mass‐analyzed (see Supporting Information).

### Mass spectrometry

MALDI mass spectra were recorded using a JMS‐S3000 SpiralTOF™ mass spectrometer (JEOL, Tokyo, Japan). A Nd:YLF laser irradiated the spots made from the deposition of 1 μL of a matrix/salt/sample solution (5 μL of DCTB at 20 mg mL^–1^ in THF, 1 μL of NaTFA at 2 mg mL^–1^ in THF and 1 μL of the aliquot from a SEC single elution) on a target plate and allowed to air‐dry. DCTB has been expressly chosen as it provides the best signal‐to‐noise (S/N) ratios at a low laser fluence, then allowing high resolution to be achieved. In addition, it has been found that the use of alternative matrices such as dithranol or 2,5‐DHB not only dramatically deteriorates the S/N ratios but also favors the detection of the VA‐rich oligomers detrimentally to the E‐rich species, leading to overestimations of the VA content (data not shown). The so‐generated ions were accelerated by a 20 kV high voltage and went through the SpiralTOF analyzer along a spiral trajectory (approximate path length: 17 m) before their detection.^32^ The delay time was set according to the mass range of the sample (230 ns for fractions #3, 240 ns for fractions #2 and 350 ns for fractions #1) to keep the peak width ΔM<0.03 Da at FWHM over the mass range of interest. Calibration was performed externally using the sodium adducts of a poly(methyl methacrylate) standard (DCTB, no salt added).

### ^13^C NMR

^13^C and DEPT135 NMR spectra were recorded using a JNM‐ECX400 spectrometer (JEOL, Tokyo, Japan) at the ^13^C resonance frequency of 100 MHz. Samples were dissolved in deuterated chloroform (CDCl_3_) at 50°C and analyzed at 30°C (accumulated spectra: 3000 for ^13^C NMR and 1440 for DEPT135 NMR) using a TH5ATFG2 5 mm probe. Assignment of ^13^C NMR signals was done according to values referenced in the literature.^10^

### Kendrick mass defect analysis

As proposed by Sato *et al*.,^31^ using the repeating unit of a polymeric backbone as the base unit for the Kendrick masses (further noted KM) allows a fast visualization of homologue series in a Kendrick mass defect plot (shortened in KMD plot). In the case of EVA copolymers, KM could be indifferently calculated using E or VA as the base unit. The E unit (C_2_H_4_, m_IUPAC_ = 28.03130 Da) has been chosen here for the sake of simplicity as a simple extension of the methylene unit (CH_2_, m_IUPAC_ = 14.01565 Da) traditionally used for fuels.^33^ Accurate mass measurements on the IUPAC scale of the co‐oligomeric adducts from EVA samples are converted into Kendrick masses with E as the base unit as follows:

(1) $$\mathrm{KM}\left(\text{oligomer}\right)=m_{\text{IUPAC}}\left(\text{oligomer}\right)\cdot \frac{28}{28.03130}$$

The KM values are then decomposed in the nominal Kendrick mass (the rounded KM to the next highest integer, noted NKM) and the Kendrick mass defect (noted KMD) calculated as follows:

(2) $$\mathrm{KMD}\left(\text{oligomer}\right)=\mathrm{NKM}\left(\text{oligomer}\right)-\mathrm{KM}\left(\text{oligomer}\right)$$

The KMD plot displays the KMD of the detected oligomeric adducts as a function of their NKM using a 'bubble chart' where each disk expresses a data triplet (NKM, KMD, abundance). The size of the disk reflects the abundance of the associated oligomer in the mass spectrum. Species containing the same number of VA units within their skeleton will line up horizontally (variation of the number of E units only) while points will shift to the oblique direction if varying their content of VA units (with the number of E units being constant).

## Results and Discussion

### Comments on the mass analysis of EVA copolymers

**Figure 1:**
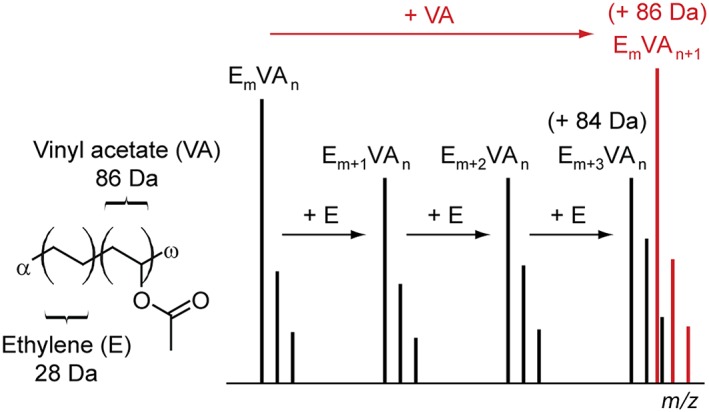
Left: generic structure of an EVA copolymer with undefined α/ω terminations. Right: Schematic representation of the isobaric issue arising from the masses of the repeating units. Adding one VA unit or three E units to an E_m_VA_n_ oligomer leads to +86 Da shifted E_m_VA_n+1_ or +84 Da shifted E_m+3_VA_n_, respectively. The ^13^C_2_ isotope of E_m+3_VA_n_ and the monoisotopic peak of E_m_VA_n+1_ are isobaric.

EVA copolymers are composed of a varying number of ethylene units (C_2_H_4_, 28.0313 Da) and vinyl acetate units (C_4_H_6_O_2_, 86.0368 Da). The generic structure of an EVA copolymer with undefined initiating and terminating end‐groups (noted α and ω) is depicted on the left in Fig. 1. A rapid inspection of the masses of these repeating units provides evidence of two overlapping issues which will further dictate the requirements for a proper mass analysis.

Starting from a hypothetic E_m_VA_n_ co‐oligomer (i.e. a copolymeric chain containing m ethylene units and n vinyl acetate units, regardless of the end‐groups and the adducted cation), adding one VA unit induces a +86 Da mass shift while adding *three* E units increases the *m/z* ratio of 84 Da (Fig. 1, right). E_m+3_VA_n_ and E_m_VA_n+1_ are thus spaced by 2 *m/z* only. In the high‐mass range, the resolution of MS devices is lower than 1 Da (e.g. TOF in linear mode for *m/z* exceeding 10,000) and one would thus fail at assigning the detected peaks to E_m+3_VA_n_ and/or E_m_VA_n+1_ for long polymeric chains. In addition, the ^13^C_2_ isotope of the E_m+3_VA_n_ co‐oligomer will interfere with the monoisotopic peak of E_m_VA_n+1_, both shifted by +86 Da from the first E_m_VA_n_ chain. The exact masses of E_m+3_VA_n_(^13^C_2_) and E_m_VA_n+1_ differ by 0.06 Da. Generally speaking, for species whose carbon content exceeds 100 atoms, the ^13^C isotope is more intense than the monoisotopic peak and the ^13^C_2_ isotope is half the ^12^C intensity. To ensure once more a correct peak assignment in the mass spectra of EVA copolymers for co‐oligomers exceeding 1500 g mol^–1^, isotopes should be properly differentiated using a high‐resolution mass analyzer to avoid severe biases (R >25,000 at *m/z* 1500 for the isotope separation mentioned above). In summary, a satisfactory mass analysis of EVA would hence be limited to (a) co‐oligomers of low molecular weight and (b) using a mass analyzer of high resolving power. A preliminary fractionation of commercial EVA samples by SEC followed by the MALDI mass analysis of the shortest chains using a SpiralTOF analyzer^31^, ^32^ constitutes a full analytical strategy expected to overcome the two pitfalls listed above.

### SEC‐MALDI of EVA copolymers

**Figure 2:**
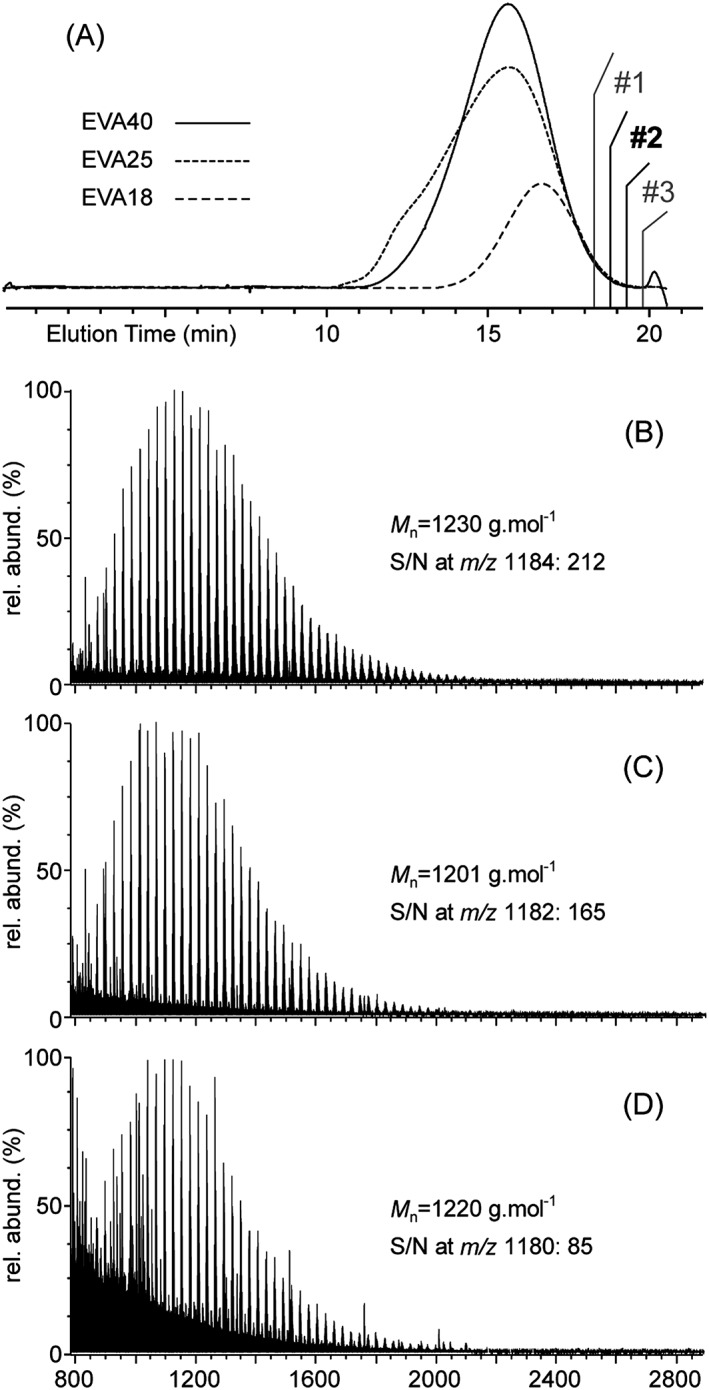
(A) SEC chromatogram of the three EVA samples in CHCl_3_ at a targeted 2 mg mL^–1^ concentration (solid line: EVA40; short dashed line: EVA25; long dashed line: EVA18). The three collected fractions #1–#3 further mass analyzed are highlighted. (B–D) MALDI mass spectra of fractions #2 collected from the SEC elution of EVA40, EVA25 and EVA18, respectively. The number average molecular weights *M*_n_ and signal‐to‐noise (S/N) ratios are listed in insets. MALDI mass spectra of fractions #3 and #1 are depicted in the Supplementary Figs. S1 and S2, respectively (Supporting Information).

The solubility of EVA samples in CHCl_3_ at room temperature has been found to depend on the VA content. EVA40 and EVA25 readily solubilize at a working concentration of 2 mg mL^–1^ while EVA18 is clearly partly insoluble at the same concentration after several days in solution. The decrease in the number of polar VA groups concomitantly with the increase in the poly(ethylene) (PE) character of EVA accounts for this behavior. The SEC chromatograms recorded for the three EVA samples (targeted concentration: 2 mg mL^–1^) are depicted in Fig. 2(A) (EVA40, solid line; EVA25: short dashed line; EVA18: long dashed line). The peak intensity clearly decreases from EVA40 to EVA18. This variation in peak intensity probably reflects the variation in sample solubility in CHCl_3_, but alternative reasons cannot be dismissed. The decrease in the VA content potentially yields a decrease in the refractive index increment (dn/dc) (found to vary with the co‐monomeric composition under certain conditions^34^) or a stronger interaction with the stationary phase. The following MS data support nevertheless the solubility variation as a highly probable hypothesis. In addition, if the elution of EVA40 and EVA25 lead to SEC chromatograms with similar shapes (number average molecular weight *M*_n_ = 42,200 g mol^–1^ for EVA40, *M*_n_ = 43,600 g.mol^–1^ for EVA25 PS equivalent), EVA18 strongly departs from this fingerprint with the detection of a peak strongly shifted towards the longer retention times (i.e. the lower molecular weights), which indicates the shortest chains only have been solubilized (*M*_n_ = 11,200 g mol^–1^ PS equivalent). The use of chlorinated/aromatic solvents such as trichlorobenzene and SEC at high temperature would allow this solubility issue to be overcome but raises the problem of the highly specific SEC system and columns to be used – not to mention the tedious evaporation step for the MALDI analysis, putting a damper on the applicability of the below‐described procedure in any laboratory. Since the mass analysis will be limited to the low molecular weight fractions following the analytical strategy commented in the first section, such a solubility issue using CHCl_3_ does nevertheless not constitute a drawback for the forthcoming characterization by MALDI‐MS. As mentioned in the introduction, the samples of interest for industrial applications are also mostly composed of more than 25 wt% VA.^3^ If EVA40 is a 'reference' material, EVA18 acts as the 'limit' sample highlighting the apparent limitations of the presently reported methodology.

The EVA chains have then been fractionated by collecting 30‐s aliquots directly after the RI detector. Of particular interest for the forthcoming high‐resolution mass analysis, the shortest chains eluting in the last minutes (18'30'' to 20' of total elution) have been collected in three aliquots numbered #1 to #3. Those fractions are highlighted in Fig. 2(A). The MALDI mass spectra subsequently recorded from the fractions #2 of the three EVA samples are depicted in Figs. 2(B) (EVA40), 2(C) (EVA25) and 2(D) (EVA18). Fractions #2 will be used throughout the text while results from fractions #1 and #3 will be systematically reported in the Supporting Information for the sake of simplicity. Contrary to the SEC fingerprints which differ between samples, the SEC‐MALDI‐MS off‐line coupling leads to similar mass spectra in terms of mass range and shape, with three Gaussian‐like distributions centered around 1220 g mol^–1^ (EVA40: *M*_n_ = 1230 g mol^–1^; EVA25: *M*_n_ = 1201 g mol^–1^ and EVA18: *M*_n_ = 1220 g mol^–1^). However, a clear deterioration of the S/N ratio is observed from EVA40 to EVA18. Matrix adducts and background peaks are detected with great abundance in the latter case. Values of S/N ratios calculated at a given *m/z* ratio (the maximum of the pattern, see Fig. 4) are listed in Figs. 2(B)–2(D), found at 212 at *m/z* 1184 for EVA40, 165 at *m/z* 1182 for EVA25 and 65 at *m/z* 1180 for EVA18. As for the solubility issue, the increasing PE‐like character of EVA when decreasing the content of VA makes the mass analysis of highly apolar chains^35^ more tedious by affecting the desorption and the ionization efficiencies. The S/N ratios calculated from the MALDI mass spectra of fractions #3 and #1 (Supplementary Figs. S1 and S2, Supporting Information) are also decreasing concomitantly with the content of VA, corroborating the above mentioned findings.

### Structural characterization of EVA chains

**Figure 3:**
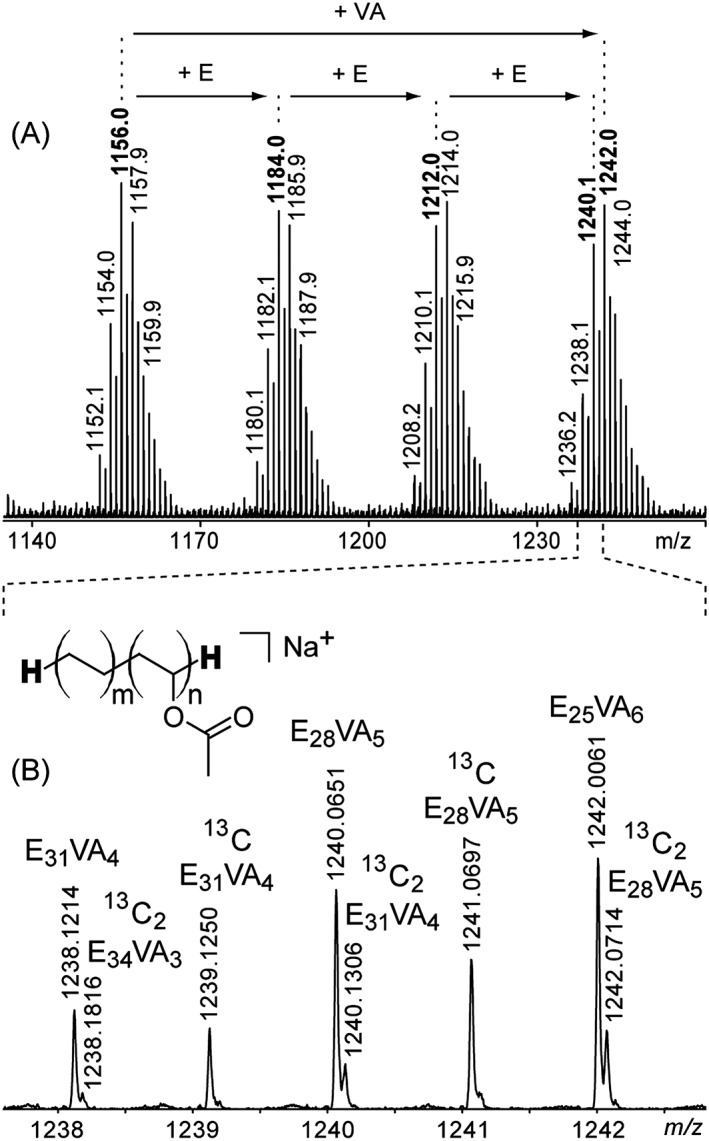
(A) Restricted mass spectrum of fraction #2 from EVA40 (*m/z* range: 1140–1260). (B) Magnification of (A) (*m/z* range: 1238–1243). Peaks are annotated with their composition in E and VA units (E_m_VA_n_). The generic structure with H as end‐groups derived from the accurate mass measurements is depicted in the inset.

The following discussion is focused on the 'reference' EVA40 for the sake of clarity. Owing to the complexity of the recorded mass spectra displayed in Fig. 2, a restricted spectrum (*m/z* range: 1140–1260) is depicted in Fig. 3(A). A set of four patterns is detected and mass shifts of 28 Da (i.e. ethylene unit E) and 86 Da (i.e. vinyl acetate unit VA) are readily evidenced. Starting from a first oligomer at *m/z* 1156.0, adding one, two and three E units leads to peaks detected at *m/z* 1184.0, 1212.0 and 1240.0, respectively, while adding one VA unit produces a peak at *m/z* 1242.0. Note the similarity of such patterns with the simulated mass spectrum depicted in Fig. 1. Looking even closer at the MS data (Fig. 3(B), *m/z* range: 1238–1243), two isobaric species clearly contribute to the signal of some peaks. The high resolving power of the SpiralTOF analyzer hence allows the expected contributions of the ^13^C isotopes to be separated from the monoisotopic peaks (as presented in Fig. 1) and ensures correct peak picking and associated assignments.

Elemental compositions have been evaluated based on the accurate mass measurements listed in Supplementary Table S1 (Supporting Information). All the detected peaks could be assigned to sodiated adducts of EVA oligomers with hydrogen as both initiating and terminating end‐group. The generic structure of oligomers found in the fraction #2 of EVA40 is depicted in the inset of Fig. 3(B). No other species with alternative terminations have been found in the mass spectrum. All the peaks detected in Fig. 3(B) could be further labelled with their respective E and VA content (in the form of E_m_VA_n_) and discriminated as either monoisotopic or ^13^C_x_ isotopic peaks. For instance, the peak at *m/z* 1240.0651 is assigned to a [H‐E_28_‐VA_5_–H+Na]^+^ adduct (shortened as E_28_VA_5_ in Fig. 3(B)) and is properly differentiated from the ^13^C_2_ isotope of [H–E_28_–VA_5_–H+Na]^+^ detected at *m/z* 1240.1306. Similar results have been obtained for EVA25 and EVA18 in terms of end‐groups with a unique H‐E_m_VA_n_‐H structure adducted with a sodium cation in both cases.

**Figure 4:**
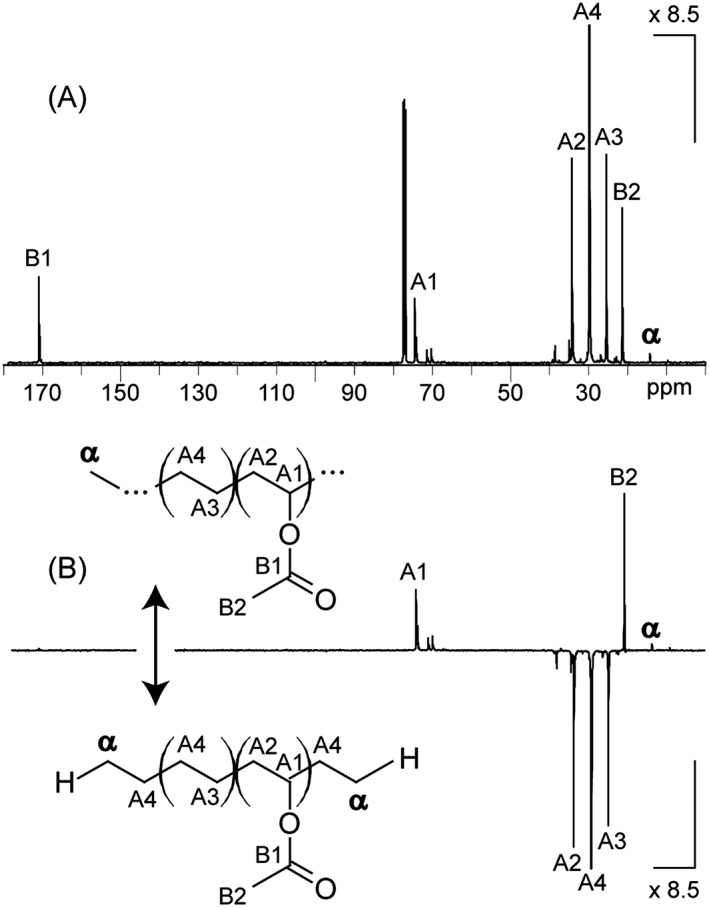
(A) ^13^C NMR spectrum and (B) DEPT135 NMR spectrum of EVA40 solubilized in CDCl_3_. Carbons from the polymeric backbone are designated with the letters A and B while the proposed methyl end‐group is designated with α.

Restricting the mass analysis to those low molecular weight fractions allows the high resolving power of the SpiralTOF to be exploited and the pitfalls mentioned in the first section to be overcome but it puts into question the extension of these structural assessments to the entire EVA sample. NMR experiments have thus been conducted on the whole sample to check the representativeness of the MS results. The ^13^C and DEPT135 NMR spectra have been recorded for the EVA40 sample and are depicted in Fig. 4.

The NMR spectra of EVA40 are extremely similar to the NMR data reported in the literature^10^, ^11^, ^12^, ^13^, ^14^ and the peak assignment has been done following the reported nomenclature.^10^ Aliphatic carbons from the VA unit are detected at 74.3 and 34.1 ppm and noted as A1 (‐CH‐, positive phase in DEPT135) and A2 (‐CH_2_‐, negative phase in DEPT135). The quaternary carbon B1 and the methyl groups of VA, noted B2, are detected at 170.8 (unseen in the DEPT135 spectrum) and 21.2 ppm (‐CH_3_, positive phase in DEPT135), respectively. The two carbon atoms constituting an ethylene monomer unit linked to a VA unit are seen at 25.3 (noted A3) and 29.6 ppm (noted A4), both of negative phase in DEPT135 (‐CH_2_‐ groups). E units not surrounded by VA units would be detected by a chemical shift similar to A4. The peak of noticeable abundance at 14.1 ppm has been assigned to another methyl group (‐CH_3_, positive phase in DEPT135) acting as chain termination and noted α. The generic structure of EVA accounting for such an NMR fingerprint is depicted in the inset at the top of Fig. 4(B). Another representation of the same EVA copolymer with methyl groups as initiating and terminating groups is depicted in the inset at the bottom of Fig. 4(B) with the VA units surrounded by E units. From the NMR point of view, such a polymeric backbone is methyl/methyl‐terminated, i.e. **CH**_**3**_‐CH_2_‐E_m_‐VA_n_‐CH_2_‐**CH**_**3**_, which is strictly identical to H/H as end‐groups from the MS point of view. The first and last carbon atoms from the first and last E units could also be seen as parts of the backbone as in **H‐CH**_**2**_‐CH_2_‐E_m_‐VA_n_‐CH_2_‐**CH**_**2**_ **‐H** which corresponds to **H**‐E_m+1_‐VA_n_‐E‐**H**. This structure is isomeric to the generic H‐E_m+2_‐VA_n_‐H depicted in Fig. 3(B). In other words, the structure of EVA40 proposed from NMR is consistent with the structure derived from the SEC‐MALDI‐MS analysis, validating the latter as a technique in its own right to characterize the EVA copolymers scrutinized in the present study from their low molecular weight fractions only with satisfactory representativeness. Consequently, the last step for a complete characterization of these commercial EVA samples which consists of the evaluation of the VA content (mol% or wt% for the molar and weight fractions, respectively) could be meaningfully conducted from the MS results only.

### Evaluation of the VA content from the native MS data

**Figure 5:**
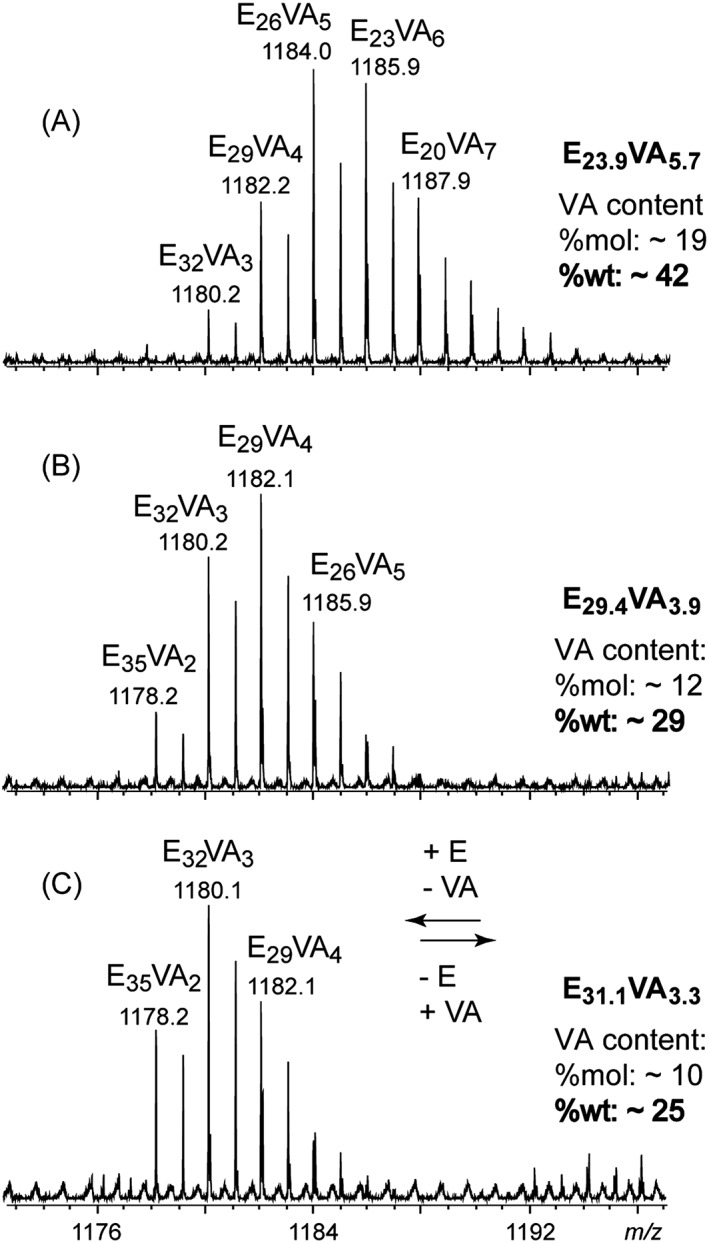
Restricted MALDI mass spectra of the fractions #2 collected from the SEC elution of (A) EVA40, (B) EVA25 and (C) EVA18 (mass range: 1173–1197). Composition in terms of ethylene (E) and vinyl acetate (VA) units are mentioned for each major peak as E_m_VA_n_ considering H as end‐group. Average compositions and computed VA contents (molar and weight) are mentioned alongside the spectra.

Magnifications of the SEC‐MALDI mass spectra of the three commercial EVA samples (*m/z* range: ~1170–1200) are depicted in Fig. 5 (A: EVA40, B: EVA25 and C: EVA18). Echoing the results from the previous section, all the detected peaks are assigned to sodium adducts of EVA oligomers with a varying number of E units (m) and VA units (n) and hydrogen atoms as end‐groups. The E_m_VA_n_ compositions are mentioned in Fig. 5 and listed in Table 1. Highlighting the performance of the SpiralTOF mass analyzer, the average error value for the assignments has been found to be +0.2 ppm.

**Table 1:** Accurate mass measurements and assignments of peaks detected in the restricted MALDI mass spectra of EVA40, EVA25 and EVA18 (Fig. 5)

	**Elemental** **Comp.**	**(*m/z*)**_**exp**_	**δ** **(ppm)**	**Assignment**	**Ab.** **(%)**	**Average** **Comp.**	**VA** **(%wt)**
**EVA40**	C_76_H_148_NaO_6_^+^	1180.1168	+0.0	[H‐(E_32_VA_3_)‐H + Na]^+^	18	E_23.9_VA_5.7_	42+/‐1
	C_74_H_142_NaO_8_^+^	1182.0589	‐0.6	[H‐(E_29_VA_4_)‐H + Na]^+^	55		
	C_72_H_136_NaO_10_^+^	1184.0030	+0.4	[H‐(E_26_VA_5_)‐H + Na]^+^	100		
	C_70_H_130_NaO_12_^+^	1185.9461	+0.5	[H‐(E_23_VA_6_)‐H + Na]^+^	96		
	C_68_H_124_NaO_14_^+^	1187.8886	+0.2	[H‐(E_20_VA_7_)‐H + Na]^+^	57		
	C_66_H_118_NaO_16_^+^	1189.8312	+0.0	[H‐(E_17_VA_8_)‐H + Na]^+^	28		
	C_64_H_112_NaO_18_^+^	1191.7737	‐0.3	[H‐(E_14_VA_9_)‐H + Na]^+^	12		
**EVA25**	C_78_H_154_NaO_4_^+^	1178.1739	+0.0	[H‐(E_35_VA_2_)‐H + Na]^+^	26	E_29.4_VA_3.9_	29+/‐2
	C_76_H_148_NaO_6_^+^	1180.1167	‐0.1	[H‐(E_32_VA_3_)‐H + Na]^+^	77		
	C_74_H_142_NaO_8_^+^	1182.0595	‐0.2	[H‐(E_29_VA_4_)‐H + Na]^+^	100		
	C_72_H_136_NaO_10_^+^	1184.0023	‐0.2	[H‐(E_26_VA_5_)‐H + Na]^+^	55		
	C_70_H_130_NaO_12_^+^	1185.9429	‐2.2	[H‐(E_23_VA_6_)‐H + Na]^+^	18		
**EVA18**	C_78_H_154_NaO_4_^+^	1178.1744	+0.4	[H‐(E_35_VA_2_)‐H + Na]^+^	49	E_31.1_VA_3.3_	25+/‐2
	C_76_H_148_NaO_6_^+^	1180.1178	+0.8	[H‐(E_32_VA_3_)‐H + Na]^+^	100		
	C_74_H_142_NaO_8_^+^	1182.0606	+0.8	[H‐(E_29_VA_4_)‐H + Na]^+^	71		
	C_72_H_136_NaO_10_^+^	1184.0068	+3.6	[H‐(E_26_VA_5_)‐H + Na]^+^	28		

VA content is derived from the composition in E and VA units of each peak convoluted by their abundance.

If the general shape of the whole mass spectrum is similar regardless of the EVA sample (Fig. 2), the magnifications depicted in Fig. 5 greatly differ between EVA40, EVA25 and EVA18. A clear shift in the pattern towards lower *m/z* ratios is observed from EVA40 to EVA18, which corresponds to an increase (resp. decrease) in the number of E (resp. VA) units within the detected oligomeric backbones (Table 1). Qualitatively speaking, MS thus allows the three samples to be discriminated. To gain more quantitative insights, the VA content has been tentatively evaluated considering the composition E_m_VA_n_ of each peak convoluted by their relative abundance to calculate an average E_m av_VA_n av_ composition. The molar content (mol%) and weight content (wt%) of VA are then derived from this average composition according to mol%(VA) = n_av_/(m_av_ + n_av_) and wt%(VA) = 86*n_av_/(28*m_av_ + 86*n_av_). Results from such calculations applied to the restricted mass spectra are mentioned for each spectrum in Fig. 5 and listed in Table 1.

VA contents of approximatively 42 wt%, 29 wt% and 25 wt% have been found for EVA40, EVA25 and EVA18, respectively. A decrease in the weight fraction of VA is thus highlighted from EVA40 to EVA18 and the values are in good agreement with the contents provided by the supplier for EVA40 and EVA25. A bias tends to appear for the last EVA18 with an overestimation of the VA content (+40% as compared to the value provided by the supplier). As for the SEC and MS measurements, EVA18 acts as the limit sample for the evaluation of the VA content. The limited solubility of EVA18 in CHCl_3_ concomitantly to desorption and ionization issues undoubtedly account for such bias. The VA content has been previously found not to vary with the molecular weight using high‐performance liquid chromatography (HPLC) measurements^36^ and we obtained similar results with a steady VA content calculated for fractions #1 and #3 (as shown in Supplementary Figs. S3 and S4, Supporting Information). A unique value for the VA content regardless of the degree of polymerization ensures once more the representativeness of the results from the low molecular weight oligomers for the whole sample. Interestingly, the calculation of the VA content conducted on the whole mass spectra gave similar results (43 wt% for EVA40, 29 wt% for EVA25 and 24 wt% for EVA18). The complete data are listed in Supplementary Tables S2–S4 (Supporting Information). Limiting the evaluation to a single pattern in lieu of the whole mass spectrum thus does not bias the VA content calculation but drastically speeds up the procedure (4 to 7 values to be treated vs. 100 to 200 values for the whole mass spectra). The composition in E and VA units has nevertheless to be evaluated manually for each point. The last section of this paper deals with the use of the Kendrick masses and KMD plots to both easily interpret the MS data in a user‐friendly representation and instantaneously calculate the VA content of any sample.

### Kendrick mass defect plots as an alternative data treatment method

**Figure 6:**
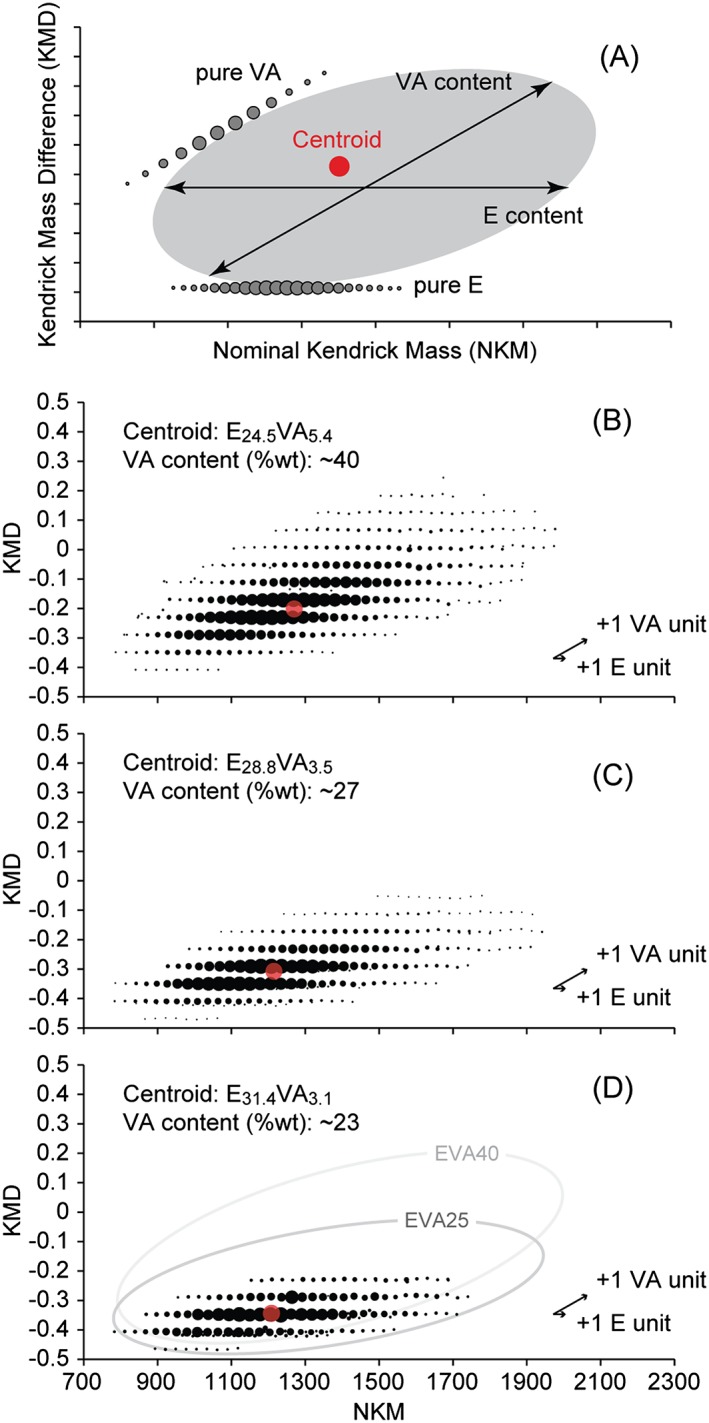
(A) Generic representation of KMD plotted as a function of NKM (so‐called KMD plot) for hypothetic pure VA and pure E polymers (oblique and horizontal lines, resp.) and EVA copolymer (grey spot). VA content is calculated from the composition in E and VA units at the centroid of the plot. The size of each disk reflects the relative abundance of the associated oligomeric adduct in the mass spectrum (so‐called bubble chart). (B)‐(D) KMD plots for the fractions #2 collected from the SEC elution of EVA40, EVA25 and EVA18 with the calculated VA content (centroid: red dot). Schematic KMD plots for EVA40 and EVA25 are reproduced in (C) for the sake of comparison (light grey and dark grey ellipses, respectively).

The Kendrick masses are calculated according to the procedure described in the Experimental section from the accurate mass measurements. Plotting the Kendrick mass defect (KMD) as a function of the nominal Kendrick masses (NKM) congeners with a varying number of base units *only* will line up horizontally.^31^ Considering E as the base unit, PE adducts would line up horizontally (same KMD value regardless of the degree of polymerization, NKM increasing by one 28 Da monomer unit at a time) while a pure VA would lead to an oblique plot, each addition of a VA unit increasing the KMD value of 0.0593 and the NKM value of 86 Da (see Fig. 6(A) for hypothetic KMD plots of pure PE and pure VA). Plotting KMD vs. NKM for an EVA copolymer for which the number of E *and* VA units vary will lead to a scatter plot with an elliptic shape, as schematically represented in Fig. 6(A) (grey spot). Such representation of the MS data allows a rapid interpretation, the dimensions of the plot in the horizontal and oblique directions giving information about the degree of polymerization in terms of E and VA units, respectively (Fig. 6(A)). An ellipse‐shaped plot with its major axis along the horizontal direction would correspond to an E‐rich aliquot while an ellipse along the oblique direction would be associated with a VA‐rich sample. The coordinates of the centroid of the KMD plot (weighted average of the NKM and KMD values) noted (NKM(centroid); KMD(centroid)) are also linked to the average n and m compositions of E and VA as follows:

(3) $$N\mathrm{KM}\left(\text{centroid}\right)=\mathrm{NKM}\left(\alpha +\omega +\text{cation}\right)+\mathrm{NKM}\left(E\right)\cdot m_{\text{centroid}}+\mathrm{NKM}\left(\mathrm{VA}\right)\cdot n_{\text{centroid}}$$

(4) $$\mathrm{KMD}\left(\text{centroid}\right)=\mathrm{KMD}\left(\alpha +\omega +\text{cation}\right)+\mathrm{KMD}\left(\mathrm{VA}\right)\cdot n_{\text{centroid}}$$

The number of VA units at the centroid n_centroid_ is obtained from Eqn. (4) according to:

(5) $$n_{\text{centroid}}=(\mathrm{KMD}\left(\text{centroid}\right)-\mathrm{KMD}\left(\alpha +\omega +\text{cation}\right)/\mathrm{KMD}\left(\mathrm{VA}\right)$$

and the number of E units at the centroid m_centroid_ is then calculated from Eqn. (3) as follows:

(6) $$m_{\text{centroid}}=\mathrm{NKM}\left(\text{centroid}\right)-\mathrm{NKM}\left(\alpha +\omega +\text{cation}\right)-\mathrm{NKM}\left(\mathrm{VA}\right)\cdot n_{\text{centroid}})/\mathrm{NKM}\left(E\right)$$

In the present case, α = ω=H and cation=Na^+^, KMD(α + ω + cation) = 0.0225, KMD(VA) = 0.0593, NKM(α + ω + cation)=25, NKM(E) = 28 and NKM(VA) = 86. Note that these equations are valid for any point in the KMD plot, which means one could use the KMD and NKM values found for any oligomer adduct to decipher its co‐monomeric composition instantaneously.

The KMD plots for the fractions #2 of EVA40, EVA25 and EVA18 are depicted in Figs. 6(B)–6(D) and the associated centroids are highlighted by a red dot. Each point of the plot corresponds to a given E_m_VA_n_ co‐oligomer. The E_m+1_VA_n_ congener is aligned on the same horizontal line (same KMD since the VA content does not change) with a NKM shift of +28 Da. The E_m_VA_n+1_ congener is shifted by +86 Da in terms of NKM and +0.0593 in terms of KMD, hence aligned in the oblique direction. The shapes of the plots are in accordance with the expected nature of the EVA aliquots. From EVA40 to EVA18, the elliptic shape tends to flatten with its major axis being closer and closer to the horizontal direction, i.e. closer to a pure PE sample. Elliptic approximations of the KMD plots for EVA40 and EVA25 are reproduced in Fig. 6(D) for sake of comparison (light and dark grey, respectively). The flattening of the plots from EVA40 to EVA18 is concomitant with a decrease in their dimension in the horizontal direction, highlighting the increasing difficulty to detect the E‐rich chains. The average compositions for EVA40, EVA25 and EVA18 calculated from the centroids have been found at E_24.5_VA_5.4_, E_28.8_VA_3.5_ and E_31.4_VA_3.1_, in other words 40 wt%, 27 wt% and 23 wt%, respectively. As an alternative *visualization* method of the native MS data, the VA contents computed from the KMD plot are similar to those computed from the MALDI mass spectra and in good agreement with the values provided by the supplier for EVA40 and EVA25, while a bias still arises for EVA18 with a noticeable overestimation. At least the correct trend (i.e. a decrease in VA content) is highlighted using either native MA data or KMD plots in a qualitative manner. Since EVA25 is the sample of interest for many industrial applications (typical VA content: 28–33 wt%), the bias observed for EVA18 should not call into question the analytical procedure proposed here. As a short digression, the KMD plots for the fractions #1 and #3 are depicted in Supplementary Figs. S5 and S6 (Supporting Information). No variation is observed over the mass range covered by the three collected fractions (*m/z* 700–3000) with VA contents (wt%) found at 41 (#1) and 40 (#3) for EVA40, 28 (#1) and 27 (#3) for EVA25 and 22 (#1) and 22 (#3) for EVA18.

It is worth mentioning that four aliquots from EVA25 collected *before* the fraction #1 discussed in the present paper have been submitted to MALDI‐MS analysis. The associated mass spectra are depicted in Supplementary Fig. S7 (Supporting Information) and display clean Gaussian‐shaped distributions (*M*_n_ = 3600, 4900, 7500 and 10,900 g mol^–1^). However, the resolution in such a high mass range is not high enough to overcome the assignment pitfalls mentioned in the first section (+2 Da between E_m+3_VA_n_ and E_m_VA_n+1_, isobaric E_m+3_VA_n_(^13^C_2_) and E_m_VA_n+1_). Proper peak assignment (composition in E and VA units) is not possible, nor is the evaluation of the VA content from either the native MS data or KMD plots. Oligomers of higher molecular weight collected from the SEC elution of EVA samples could hence be mass analyzed, but the information extracted from the so‐recorded mass spectra should be limited to the molecular weights only.

## Conclusions

First results about the characterization of commercial EVA copolymers with different VA contents using high‐resolution mass spectrometry have been reported. As a major structural feature for polymeric chains, the end‐groups of the low molecular weight oligomers were successfully characterized using a MALDI SpiralTOF apparatus following a preliminary fractionation by SEC, regardless of the VA content of the aliquots. Such VA content was also satisfactorily evaluated from the mass data for the EVA samples of industrial interest (VA content >25%) while it tended to be overestimated for the samples with low VA content owing to their increasing PE‐like character. Lastly, Kendrick mass defect analysis was proposed as an alternative data treatment method for the discrimination of the EVA samples and the evaluation of their VA content. A major advantage of the KMD plot relies on the speed of data treatment especially for copolymers. Once the end‐groups have been characterized, spectra with correct calibration can be automatically converted into KMD plots using automatic peak picking, the centroid and its associated E_m_VA_n_ determined instantaneously, that is to say the VA content, in addition to the degrees of polymerizations (shape of the KMD plot). Such a user‐friendly data treatment method would be of high interest for time‐consuming routine or comparative studies for which many samples must be analyzed.

## Supporting information

Supporting Info item
