# Supplementary material for: MALDI SpiralTOF high‐resolution mass spectrometry and Kendrick mass defect analysis applied to the characterization of poly(ethylene‐co‐vinyl acetate) copolymers
Source: Rapid Commun Mass Spectrom. 2016 Mar 8;30(7):973–81. doi: 10.1002/rcm.7525 (PMC4787217; doi:10.1002/rcm.7525)
Supplement: Supplementary file 1 — Supporting Info item [file RCM-30-973-s001.docx]

**MALDI Spiral-TOF high resolution mass spectrometry and Kendrick mass defect analysis applied to the characterization of poly(ethylene-co-vinyl acetate) copolymers**

Thierry Fouquet*, Sayaka Nakamura and Hiroaki Sato*

National Institute of Advanced Industrial Science and Technology (AIST), Environmental Measurement Technology Group, Environmental Management Research Institute (EMRI), Tsukuba, Ibaraki, Japan.

**Correspondence to: thierry.fouquet@aist.go.jp, sato-hiroaki@aist.go.jp*

**SUPPORTING INFORMATION**

**Content:**

**Figure S1 & S2.** SEC chromatogram of the three EVA samples and MALDI-MS spectra of fractions #3 (**Fig. S1**) and #1 (**Fig. S2**) collected from the SEC elutions.

**Table S1.** Accurate mass measurements and associated compositions in E and VA units from the MALDI mass spectrum of the fraction #2 collected from the SEC elution of EVA40.

**Figure S3 & S4.** Restricted MALDI-MS spectra of the fractions #3 (**Fig. S3**) and #1 (**Fig. S4**).

**Evaluation of the E and VA compositions from KMD.**

**Table S2-S4.** VA content calculated from the accurate mass measurements and compositions in E and VA units from the mass spectra of EVA40 (Table S2), EVA25 (Table S3), EVA18 (Table S4)

**Figure S5 & S6.** KMD plots from the fractions #3 (**Fig. S5**) and #1 (**Fig. S6**).

**Fig. S7.** SEC chromatogram of EVA25 and MALDI mass spectra of high molecular weight fractions.


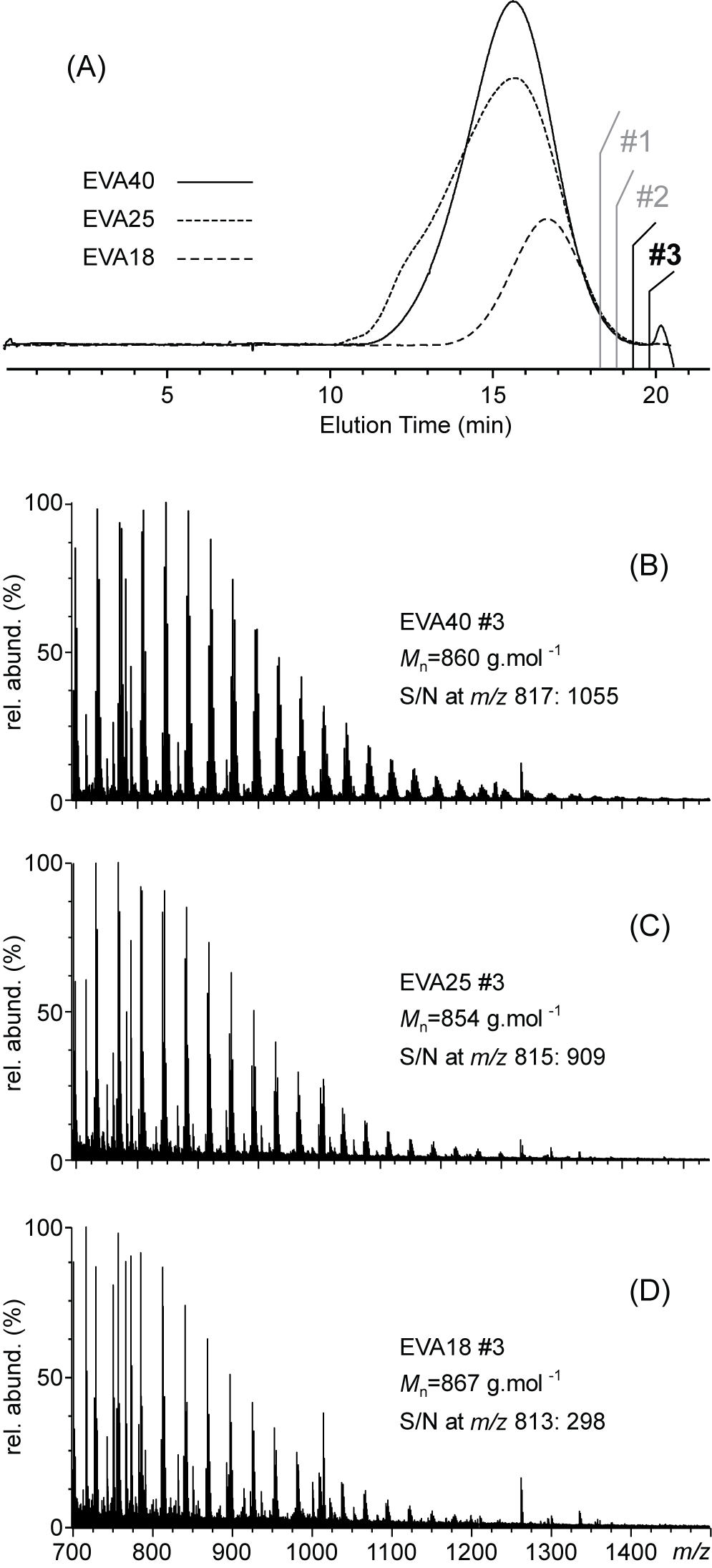


**Figure S1.** (A) SEC chromatogram of the three EVA samples in chloroform (solid line: EVA40; short dashed line: EVA25; long dashed line: EVA18). The three collected fractions #1-#3 further mass analyzed are highlighted. (B)-(D) MALDI-MS spectra of fractions #3 collected from the SEC elution of EVA40, EVA25 and EVA18, respectively. The number average molecular weights *M*_n_ and signal-to-noise ratios are listed in insets. Note the decrease in terms of S/N when decreasing the VA content from EVA40 to EVA25 and EVA18.


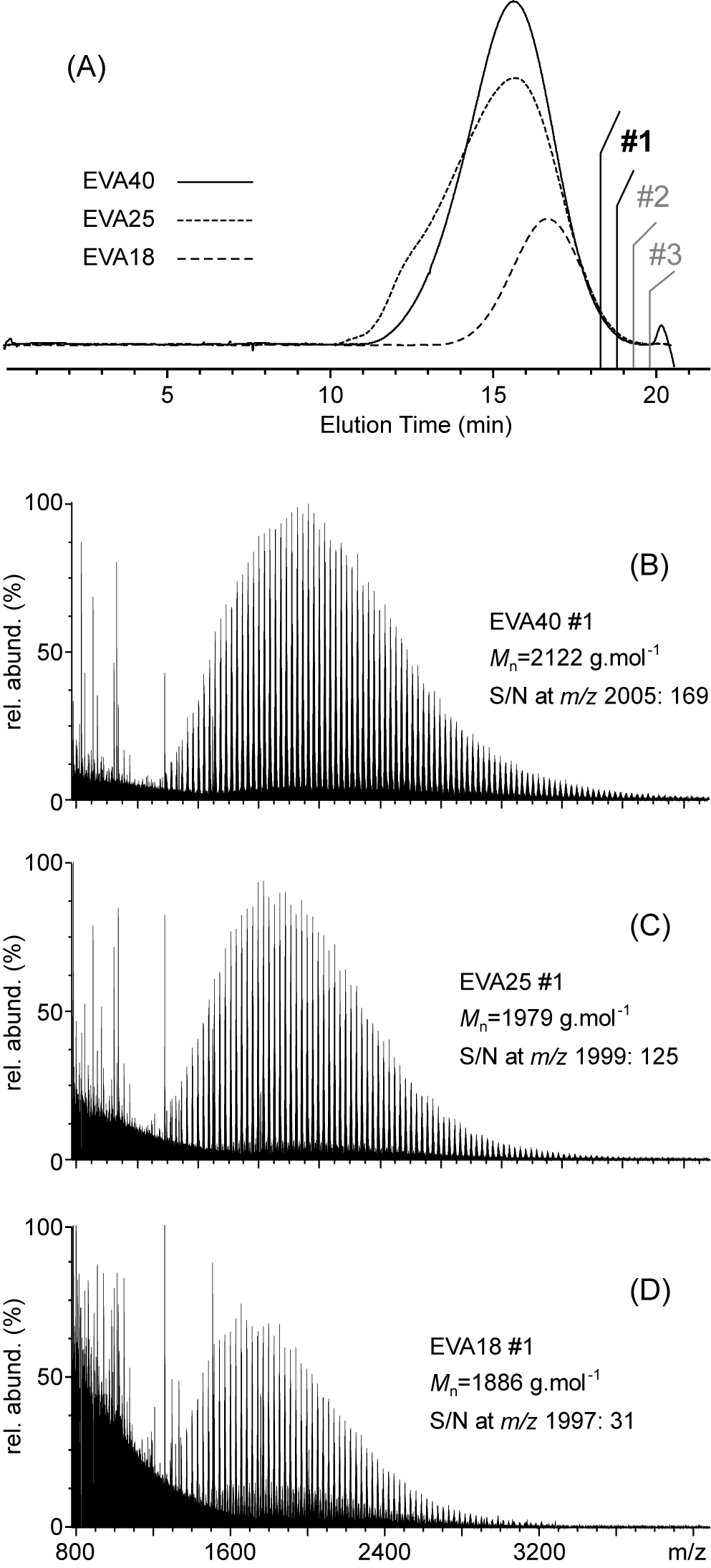


**Figure S2.** (A) SEC chromatogram of the three EVA samples in chloroform (solid line: EVA40; short dashed line: EVA25; long dashed line: EVA18). The three collected fractions #1-#3 further mass analyzed are highlighted. (B)-(D) MALDI-MS spectra of fractions #1 collected from the SEC elution of EVA40, EVA25 and EVA18, respectively. The number average molecular weights *M*_n_ and signal-to-noise ratios are listed in insets. Note both the decrease in terms of S/N and the shift towards lower *M*_n_ values when decreasing the VA content from EVA40 to EVA25 and EVA18.

The *M*_n_ calculated from the MALDI mass spectra of fractions #1 noticeably shift towards lower values for EVA25 (*M*_n_=1979 g mol^-1^) and EVA18 (*M*_n_=1886 g mol^-1^) as compared to EVA40 (*M*_n_=2122 g mol^-1^) (i.e. a difference of almost three VA units or more than eight E units between EVA18 and EVA40). Regardless of the sample (EVA40, EVA25 or EVA18), chains of “high molecular weight” containing too many E units and not enough VA units (to promote interactions with the cation) are undoubtedly under detected. In EVA25 and EVA18, those chains are more preponderant by nature than in EVA40 and their under detection leads to a bias in the average molecular weight. However it is unclear if one Mn is underestimated (EVA18 or EVA25) or if the other is overestimated (EVA40).

**Table S1.** Accurate mass measurements and associated compositions in E and VA units from the MALDI mass spectrum of the fraction #2 collected from the SEC elution of EVA40.

**Generic composition: H-(E)_m_-(VA)_n_-H + Na^+^**

| \| **Elemental composition** \| **(*m/z*)_th_** \| **(*m/z*)_expe_** \| **Err (ppm)** \| **E** \| **VA** \| \| --- \| --- \| --- \| --- \| --- \| --- \| \| C_54_H_104_O_6_Na^+^ \| 871.7725 \| 871.7722 \| -0.3 \| 21 \| 3 \| \| C_52_H_98_O_8_Na^+^ \| 873.7154 \| 873.7156 \| 0.2 \| 18 \| 4 \| \| C_50_H_92_O_10_Na^+^ \| 875.6583 \| 875.6587 \| 0.5 \| 15 \| 5 \| \| C_48_H_86_O_12_Na^+^ \| 877.6011 \| 877.6020 \| 0.9 \| 12 \| 6 \| \| C_56_H_108_O_6_Na^+^ \| 899.8038 \| 899.8031 \| -0.8 \| 22 \| 3 \| \| C_54_H_102_O_8_Na^+^ \| 901.7467 \| 901.7461 \| -0.6 \| 19 \| 4 \| \| C_52_H_96_O_10_Na^+^ \| 903.6896 \| 903.6891 \| -0.5 \| 16 \| 5 \| \| C_50_H_90_O_12_Na^+^ \| 905.6324 \| 905.6317 \| -0.8 \| 13 \| 6 \| \| C_60_H_118_O_4_Na^+^ \| 925.8922 \| 925.8917 \| -0.6 \| 26 \| 2 \| \| C_58_H_112_O_6_Na^+^ \| 927.8351 \| 927.8351 \| 0.0 \| 23 \| 3 \| \| C_56_H_106_O_8_Na^+^ \| 929.7780 \| 929.7784 \| 0.4 \| 20 \| 4 \| \| C_54_H_100_O_10_Na^+^ \| 931.7209 \| 931.7204 \| -0.4 \| 17 \| 5 \| \| C_52_H_94_O_12_Na^+^ \| 933.6637 \| 933.6620 \| -1.8 \| 14 \| 6 \| \| C_62_H_122_O_4_Na^+^ \| 953.9235 \| 953.9230 \| -0.6 \| 27 \| 2 \| \| C_60_H_116_O_6_Na^+^ \| 955.8664 \| 955.8667 \| 0.3 \| 24 \| 3 \| \| C_58_H_110_O_8_Na^+^ \| 957.8093 \| 957.8092 \| -0.1 \| 21 \| 4 \| \| C_56_H_104_O_10_Na^+^ \| 959.7522 \| 959.7526 \| 0.5 \| 18 \| 5 \| \| C_54_H_98_O_12_Na^+^ \| 961.6950 \| 961.6944 \| -0.7 \| 15 \| 6 \| \| C_52_H_92_O_14_Na^+^ \| 963.6379 \| 963.6378 \| -0.2 \| 12 \| 7 \| \| C_64_H_126_O_4_Na^+^ \| 981.9548 \| 981.9551 \| 0.2 \| 28 \| 2 \| \| C_62_H_120_O_6_Na^+^ \| 983.8977 \| 983.8973 \| -0.4 \| 25 \| 3 \| \| C_60_H_114_O_8_Na^+^ \| 985.8406 \| 985.8406 \| 0.0 \| 22 \| 4 \| \| C_58_H_108_O_10_Na^+^ \| 987.7835 \| 987.7831 \| -0.3 \| 19 \| 5 \| \| C_56_H_102_O_12_Na^+^ \| 989.7263 \| 989.7260 \| -0.3 \| 16 \| 6 \| \| C_54_H_96_O_14_Na^+^ \| 991.6692 \| 991.6686 \| -0.6 \| 13 \| 7 \| \| C_64_H_124_O_6_Na^+^ \| 1011.9290 \| 1011.9284 \| -0.6 \| 26 \| 3 \| \| C_62_H_118_O_8_Na^+^ \| 1013.8719 \| 1013.8725 \| 0.6 \| 23 \| 4 \| \| C_60_H_112_O_10_Na^+^ \| 1015.8148 \| 1015.8153 \| 0.6 \| 20 \| 5 \| \| C_58_H_106_O_12_Na^+^ \| 1017.7576 \| 1017.7574 \| -0.2 \| 17 \| 6 \| \| C_56_H_100_O_14_Na^+^ \| 1019.7005 \| 1019.6996 \| -0.9 \| 14 \| 7 \| \| C_66_H_128_O_6_Na^+^ \| 1039.9603 \| 1039.9604 \| 0.1 \| 27 \| 3 \| \| C_64_H_122_O_8_Na^+^ \| 1041.9032 \| 1041.9034 \| 0.2 \| 24 \| 4 \| \| C_62_H_116_O_10_Na^+^ \| 1043.8461 \| 1043.8460 \| -0.1 \| 21 \| 5 \| \| C_60_H_110_O_12_Na^+^ \| 1045.7889 \| 1045.7893 \| 0.3 \| 18 \| 6 \| \| C_58_H_104_O_14_Na^+^ \| 1047.7318 \| 1047.7318 \| 0.0 \| 15 \| 7 \| \| C_56_H_98_O_16_Na^+^ \| 1049.6747 \| 1049.6758 \| 1.0 \| 12 \| 8 \| \| C_68_H_132_O_6_Na^+^ \| 1067.9916 \| 1067.9920 \| 0.3 \| 28 \| 3 \| \| C_66_H_126_O_8_Na^+^ \| 1069.9345 \| 1069.9342 \| -0.3 \| 25 \| 4 \| \| C_64_H_120_O_10_Na^+^ \| 1071.8774 \| 1071.8775 \| 0.1 \| 22 \| 5 \| \| C_62_H_114_O_12_Na^+^ \| 1073.8202 \| 1073.8201 \| -0.2 \| 19 \| 6 \| \| C_60_H_108_O_14_Na^+^ \| 1075.7631 \| 1075.7632 \| 0.0 \| 16 \| 7 \| \| C_58_H_102_O_16_Na^+^ \| 1077.7060 \| 1077.7064 \| 0.4 \| 13 \| 8 \| \| C_70_H_136_O_6_Na^+^ \| 1096.0229 \| 1096.0224 \| -0.4 \| 29 \| 3 \| | \| **Elemental composition** \| **(*m/z*)_th_** \| **(*m/z*)_expe_** \| **Err (ppm)** \| **E** \| **VA** \| \| --- \| --- \| --- \| --- \| --- \| --- \| \| C_68_H_130_O_8_Na^+^ \| 1097.9658 \| 1097.9660 \| 0.2 \| 26 \| 4 \| \| C_66_H_124_O_10_Na^+^ \| 1099.9087 \| 1099.9083 \| -0.4 \| 23 \| 5 \| \| C_64_H_118_O_12_Na^+^ \| 1101.8515 \| 1101.8516 \| 0.0 \| 20 \| 6 \| \| C_62_H_112_O_14_Na^+^ \| 1103.7944 \| 1103.7948 \| 0.3 \| 17 \| 7 \| \| C_60_H_106_O_16_Na^+^ \| 1105.7373 \| 1105.7371 \| -0.2 \| 14 \| 8 \| \| C_72_H_140_O_6_Na^+^ \| 1124.0542 \| 1124.0540 \| -0.2 \| 30 \| 3 \| \| C_70_H_134_O_8_Na^+^ \| 1125.9971 \| 1125.9969 \| -0.1 \| 27 \| 4 \| \| C_68_H_128_O_10_Na^+^ \| 1127.9400 \| 1127.9406 \| 0.5 \| 24 \| 5 \| \| C_66_H_122_O_12_Na^+^ \| 1129.8828 \| 1129.8830 \| 0.2 \| 21 \| 6 \| \| C_64_H_116_O_14_Na^+^ \| 1131.8257 \| 1131.8260 \| 0.2 \| 18 \| 7 \| \| C_62_H_110_O_16_Na^+^ \| 1133.7686 \| 1133.7685 \| -0.1 \| 15 \| 8 \| \| C_74_H_144_O_6_Na^+^ \| 1152.0855 \| 1152.0861 \| 0.5 \| 31 \| 3 \| \| C_72_H_138_O_8_Na^+^ \| 1154.0284 \| 1154.0281 \| -0.3 \| 28 \| 4 \| \| C_70_H_132_O_10_Na^+^ \| 1155.9713 \| 1155.9710 \| -0.2 \| 25 \| 5 \| \| C_68_H_126_O_12_Na^+^ \| 1157.9141 \| 1157.9137 \| -0.4 \| 22 \| 6 \| \| C_66_H_120_O_14_Na^+^ \| 1159.8570 \| 1159.8572 \| 0.2 \| 19 \| 7 \| \| C_64_H_114_O_16_Na^+^ \| 1161.7999 \| 1161.7993 \| -0.5 \| 16 \| 8 \| \| C_76_H_148_O_6_Na^+^ \| 1180.1168 \| 1180.1168 \| 0.0 \| 32 \| 3 \| \| C_74_H_142_O_8_Na^+^ \| 1182.0597 \| 1182.0589 \| -0.6 \| 29 \| 4 \| \| C_72_H_136_O_10_Na^+^ \| 1184.0026 \| 1184.0030 \| 0.4 \| 26 \| 5 \| \| C_70_H_130_O_12_Na^+^ \| 1185.9454 \| 1185.9461 \| 0.5 \| 23 \| 6 \| \| C_68_H_124_O_14_Na^+^ \| 1187.8883 \| 1187.8886 \| 0.2 \| 20 \| 7 \| \| C_66_H_118_O_16_Na^+^ \| 1189.8312 \| 1189.8312 \| 0.0 \| 17 \| 8 \| \| C_64_H_112_O_18_Na^+^ \| 1191.7741 \| 1191.7737 \| -0.3 \| 14 \| 9 \| \| C_78_H_152_O_6_Na^+^ \| 1208.1481 \| 1208.1479 \| -0.2 \| 33 \| 3 \| \| C_76_H_146_O_8_Na^+^ \| 1210.0910 \| 1210.0904 \| -0.5 \| 30 \| 4 \| \| C_74_H_140_O_10_Na^+^ \| 1212.0339 \| 1212.0339 \| 0.0 \| 27 \| 5 \| \| C_72_H_134_O_12_Na^+^ \| 1213.9767 \| 1213.9766 \| -0.2 \| 24 \| 6 \| \| C_70_H_128_O_14_Na^+^ \| 1215.9196 \| 1215.9196 \| 0.0 \| 21 \| 7 \| \| C_68_H_122_O_16_Na^+^ \| 1217.8625 \| 1217.8618 \| -0.6 \| 18 \| 8 \| \| C_66_H_116_O_18_Na^+^ \| 1219.8054 \| 1219.8052 \| -0.2 \| 15 \| 9 \| \| C_80_H_156_O_6_Na^+^ \| 1236.1794 \| 1236.1780 \| -1.1 \| 34 \| 3 \| \| C_80_H_156_O_6_Na^+^ \| 1238.1223 \| 1238.1214 \| -0.7 \| 31 \| 4 \| \| C_80_H_156_O_6_Na^+^ \| 1240.0652 \| 1240.0651 \| -0.1 \| 28 \| 5 \| \| C_80_H_156_O_6_Na^+^ \| 1242.0080 \| 1242.0081 \| 0.0 \| 25 \| 6 \| \| C_80_H_156_O_6_Na^+^ \| 1243.9509 \| 1243.9496 \| -1.0 \| 22 \| 7 \| \| C_80_H_156_O_6_Na^+^ \| 1245.8938 \| 1245.8935 \| -0.2 \| 19 \| 8 \| \| C_80_H_156_O_6_Na^+^ \| 1247.8367 \| 1247.8359 \| -0.6 \| 16 \| 9 \| \| C_80_H_154_O_8_Na^+^ \| 1266.1536 \| 1266.1576 \| 3.2 \| 32 \| 4 \| \| C_78_H_148_O_10_Na^+^ \| 1268.0965 \| 1268.0962 \| -0.2 \| 29 \| 5 \| \| C_76_H_142_O_12_Na^+^ \| 1270.0393 \| 1270.0388 \| -0.4 \| 26 \| 6 \| \| C_74_H_136_O_14_Na^+^ \| 1271.9822 \| 1271.9819 \| -0.3 \| 23 \| 7 \| \| C_72_H_130_O_16_Na^+^ \| 1273.9251 \| 1273.9253 \| 0.2 \| 20 \| 8 \| |
| --- | --- | --- | --- | --- | --- | --- | --- | --- | --- | --- | --- | --- | --- | --- | --- | --- | --- | --- | --- | --- | --- | --- | --- | --- | --- | --- | --- | --- | --- | --- | --- | --- | --- | --- | --- | --- | --- | --- | --- | --- | --- | --- | --- | --- | --- | --- | --- | --- | --- | --- | --- | --- | --- | --- | --- | --- | --- | --- | --- | --- | --- | --- | --- | --- | --- | --- | --- | --- | --- | --- | --- | --- | --- | --- | --- | --- | --- | --- | --- | --- | --- | --- | --- | --- | --- | --- | --- | --- | --- | --- | --- | --- | --- | --- | --- | --- | --- | --- | --- | --- | --- | --- | --- | --- | --- | --- | --- | --- | --- | --- | --- | --- | --- | --- | --- | --- | --- | --- | --- | --- | --- | --- | --- | --- | --- | --- | --- | --- | --- | --- | --- | --- | --- | --- | --- | --- | --- | --- | --- | --- | --- | --- | --- | --- | --- | --- | --- | --- | --- | --- | --- | --- | --- | --- | --- | --- | --- | --- | --- | --- | --- | --- | --- | --- | --- | --- | --- | --- | --- | --- | --- | --- | --- | --- | --- | --- | --- | --- | --- | --- | --- | --- | --- | --- | --- | --- | --- | --- | --- | --- | --- | --- | --- | --- | --- | --- | --- | --- | --- | --- | --- | --- | --- | --- | --- | --- | --- | --- | --- | --- | --- | --- | --- | --- | --- | --- | --- | --- | --- | --- | --- | --- | --- | --- | --- | --- | --- | --- | --- | --- | --- | --- | --- | --- | --- | --- | --- | --- | --- | --- | --- | --- | --- | --- | --- | --- | --- | --- | --- | --- | --- | --- | --- | --- | --- | --- | --- | --- | --- | --- | --- | --- | --- | --- | --- | --- | --- | --- | --- | --- | --- | --- | --- | --- | --- | --- | --- | --- | --- | --- | --- | --- | --- | --- | --- | --- | --- | --- | --- | --- | --- | --- | --- | --- | --- | --- | --- | --- | --- | --- | --- | --- | --- | --- | --- | --- | --- | --- | --- | --- | --- | --- | --- | --- | --- | --- | --- | --- | --- | --- | --- | --- | --- | --- | --- | --- | --- | --- | --- | --- | --- | --- | --- | --- | --- | --- | --- | --- | --- | --- | --- | --- | --- | --- | --- | --- | --- | --- | --- | --- | --- | --- | --- | --- | --- | --- | --- | --- | --- | --- | --- | --- | --- | --- | --- | --- | --- | --- | --- | --- | --- | --- | --- | --- | --- | --- | --- | --- | --- | --- | --- | --- | --- | --- | --- | --- | --- | --- | --- | --- | --- | --- | --- | --- | --- | --- | --- | --- | --- | --- | --- | --- | --- | --- | --- | --- | --- | --- | --- | --- | --- | --- | --- | --- | --- | --- | --- | --- | --- | --- | --- | --- | --- | --- | --- | --- | --- | --- | --- | --- | --- | --- | --- | --- | --- | --- | --- | --- | --- | --- | --- | --- | --- | --- | --- | --- | --- | --- | --- | --- | --- | --- | --- | --- | --- | --- | --- | --- | --- | --- | --- | --- | --- | --- | --- | --- | --- | --- | --- | --- | --- | --- | --- | --- | --- | --- | --- | --- | --- | --- | --- | --- | --- | --- | --- | --- | --- | --- | --- | --- | --- | --- | --- | --- | --- | --- | --- | --- | --- | --- | --- | --- | --- | --- | --- | --- | --- | --- | --- | --- | --- | --- | --- | --- | --- | --- | --- | --- | --- | --- | --- | --- | --- | --- | --- | --- | --- | --- | --- |

**Table S1** (next)

| \| **Elemental composition** \| **(*m/z*)_th_** \| **(*m/z*)_expe_** \| **Err (ppm)** \| **E** \| **VA** \| \| --- \| --- \| --- \| --- \| --- \| --- \| \| C_70_H_124_O_18_Na^+^ \| 1275.8680 \| 1275.8691 \| 0.9 \| 17 \| 9 \| \| C_68_H_118_O_20_Na^+^ \| 1277.8109 \| 1277.8114 \| 0.4 \| 14 \| 10 \| \| C_82_H_158_O_8_Na^+^ \| 1294.1849 \| 1294.1876 \| 2.1 \| 33 \| 4 \| \| C_80_H_152_O_10_Na^+^ \| 1296.1278 \| 1296.1274 \| -0.3 \| 30 \| 5 \| \| C_78_H_146_O_12_Na^+^ \| 1298.0706 \| 1298.0701 \| -0.4 \| 27 \| 6 \| \| C_76_H_140_O_14_Na^+^ \| 1300.0135 \| 1300.0125 \| -0.8 \| 24 \| 7 \| \| C_74_H_134_O_16_Na^+^ \| 1301.9564 \| 1301.9558 \| -0.5 \| 21 \| 8 \| \| C_72_H_128_O_18_Na^+^ \| 1303.8993 \| 1303.8981 \| -0.9 \| 18 \| 9 \| \| C_70_H_122_O_20_Na^+^ \| 1305.8422 \| 1305.8411 \| -0.8 \| 15 \| 10 \| \| C_84_H_162_O_8_Na^+^ \| 1322.2162 \| 1322.2251 \| 6.7 \| 34 \| 4 \| \| C_82_H_156_O_10_Na^+^ \| 1324.1591 \| 1324.1619 \| 2.1 \| 31 \| 5 \| \| C_80_H_150_O_12_Na^+^ \| 1326.1019 \| 1326.0975 \| -3.4 \| 28 \| 6 \| \| C_78_H_144_O_14_Na^+^ \| 1328.0448 \| 1328.0463 \| 1.1 \| 25 \| 7 \| \| C_76_H_138_O_16_Na^+^ \| 1329.9877 \| 1329.9930 \| 4.0 \| 22 \| 8 \| \| C_74_H_132_O_18_Na^+^ \| 1331.9306 \| 1331.9420 \| 8.6 \| 19 \| 9 \| \| C_72_H_126_O_20_Na^+^ \| 1333.8735 \| 1333.8792 \| 4.3 \| 16 \| 10 \| \| C_86_H_166_O_8_Na^+^ \| 1350.2475 \| 1350.2523 \| 3.6 \| 35 \| 4 \| \| C_84_H_160_O_10_Na^+^ \| 1352.1904 \| 1352.1789 \| -8.5 \| 32 \| 5 \| \| C_82_H_154_O_12_Na^+^ \| 1354.1332 \| 1354.1361 \| 2.1 \| 29 \| 6 \| \| C_80_H_148_O_14_Na^+^ \| 1356.0761 \| 1356.0853 \| 6.8 \| 26 \| 7 \| \| C_78_H_142_O_16_Na^+^ \| 1358.0190 \| 1358.0263 \| 5.4 \| 23 \| 8 \| \| C_76_H_136_O_18_Na^+^ \| 1359.9619 \| 1359.9582 \| -2.7 \| 20 \| 9 \| \| C_74_H_130_O_20_Na^+^ \| 1361.9048 \| 1361.9060 \| 0.9 \| 17 \| 10 \| \| C_88_H_170_O_8_Na^+^ \| 1378.2788 \| 1378.2785 \| -0.2 \| 36 \| 4 \| \| C_86_H_170_O_10_Na^+^ \| 1380.2217 \| 1380.2199 \| -1.2 \| 33 \| 5 \| \| C_84_H_164_O_12_Na^+^ \| 1382.1645 \| 1382.1592 \| -3.8 \| 30 \| 6 \| \| C_82_H_158_O_14_Na^+^ \| 1384.1074 \| 1384.1038 \| -2.6 \| 27 \| 7 \| \| C_80_H_152_O_16_Na^+^ \| 1386.0503 \| 1386.0463 \| -2.9 \| 24 \| 8 \| \| C_78_H_146_O_18_Na^+^ \| 1387.9932 \| 1387.9908 \| -1.7 \| 21 \| 9 \| \| C_76_H_140_O_20_Na^+^ \| 1389.9361 \| 1389.9354 \| -0.5 \| 18 \| 10 \| \| C_74_H_134_O_22_Na^+^ \| 1391.8789 \| 1391.8778 \| -0.8 \| 15 \| 11 \| \| C_90_H_174_O_8_Na^+^ \| 1406.3101 \| 1406.3101 \| 0.0 \| 37 \| 4 \| \| C_88_H_168_O_10_Na^+^ \| 1408.2530 \| 1408.2599 \| 4.9 \| 34 \| 5 \| \| C_86_H_162_O_12_Na^+^ \| 1410.1958 \| 1410.1887 \| -5.1 \| 31 \| 6 \| \| C_84_H_156_O_14_Na^+^ \| 1412.1387 \| 1412.1417 \| 2.1 \| 28 \| 7 \| \| C_82_H_150_O_16_Na^+^ \| 1414.0816 \| 1414.0862 \| 3.3 \| 25 \| 8 \| \| C_80_H_144_O_18_Na^+^ \| 1416.0245 \| 1416.0224 \| -1.5 \| 22 \| 9 \| \| C_78_H_138_O_20_Na^+^ \| 1417.9674 \| 1417.9669 \| -0.3 \| 19 \| 10 \| \| C_76_H_132_O_22_Na^+^ \| 1419.9102 \| 1419.9125 \| 1.6 \| 16 \| 11 \| \| C_92_H_178_O_8_Na^+^ \| 1434.3414 \| 1434.3289 \| -8.7 \| 38 \| 4 \| \| C_90_H_172_O_10_Na^+^ \| 1436.2843 \| 1436.2872 \| 2.1 \| 35 \| 5 \| \| C_88_H_166_O_12_Na^+^ \| 1438.2271 \| 1438.2318 \| 3.2 \| 32 \| 6 \| \| C_86_H_160_O_14_Na^+^ \| 1440.1700 \| 1440.1658 \| -2.9 \| 29 \| 7 \| \| C_84_H_154_O_16_Na^+^ \| 1442.1129 \| 1442.1104 \| -1.8 \| 26 \| 8 \| | \| **Elemental composition** \| **(*m/z*)_th_** \| **(*m/z*)_expe_** \| **Err (ppm)** \| **E** \| **VA** \| \| --- \| --- \| --- \| --- \| --- \| --- \| \| C_82_H_148_O_18_Na^+^ \| 1444.0558 \| 1444.0549 \| -0.6 \| 23 \| 9 \| \| C_80_H_142_O_20_Na^+^ \| 1445.9987 \| 1446.0027 \| 2.8 \| 20 \| 10 \| \| C_78_H_136_O_22_Na^+^ \| 1447.9415 \| 1447.9398 \| -1.2 \| 17 \| 11 \| \| C_92_H_176_O_10_Na^+^ \| 1464.3156 \| 1464.3198 \| 2.9 \| 36 \| 5 \| \| C_90_H_170_O_12_Na^+^ \| 1466.2584 \| 1466.2644 \| 4.0 \| 33 \| 6 \| \| C_88_H_164_O_14_Na^+^ \| 1468.2013 \| 1468.1963 \| -3.4 \| 30 \| 7 \| \| C_86_H_158_O_16_Na^+^ \| 1470.1442 \| 1470.1461 \| 1.3 \| 27 \| 8 \| \| C_84_H_152_O_18_Na^+^ \| 1472.0871 \| 1472.0801 \| -4.7 \| 24 \| 9 \| \| C_82_H_146_O_20_Na^+^ \| 1474.0300 \| 1474.0353 \| 3.6 \| 21 \| 10 \| \| C_80_H_140_O_22_Na^+^ \| 1475.9728 \| 1475.9724 \| -0.3 \| 18 \| 11 \| \| C_78_H_134_O_24_Na^+^ \| 1477.9157 \| 1477.9202 \| 3.0 \| 15 \| 12 \| \| C_94_H_180_O_10_Na^+^ \| 1492.3469 \| 1492.3547 \| 5.3 \| 37 \| 5 \| \| C_92_H_174_O_12_Na^+^ \| 1494.2897 \| 1494.2907 \| 0.6 \| 34 \| 6 \| \| C_90_H_168_O_14_Na^+^ \| 1496.2326 \| 1496.2369 \| 2.9 \| 31 \| 7 \| \| C_88_H_162_O_16_Na^+^ \| 1498.1755 \| 1498.1728 \| -1.8 \| 28 \| 8 \| \| C_86_H_156_O_18_Na^+^ \| 1500.1184 \| 1500.1122 \| -4.1 \| 25 \| 9 \| \| C_84_H_150_O_20_Na^+^ \| 1502.0613 \| 1502.0619 \| 0.4 \| 22 \| 10 \| \| C_82_H_144_O_22_Na^+^ \| 1504.0041 \| 1504.0006 \| -2.3 \| 19 \| 11 \| \| C_96_H_184_O_10_Na^+^ \| 1520.3782 \| 1520.3788 \| 0.4 \| 38 \| 5 \| \| C_94_H_178_O_12_Na^+^ \| 1522.3210 \| 1522.3369 \| 10.4 \| 35 \| 6 \| \| C_92_H_172_O_14_Na^+^ \| 1524.2639 \| 1524.2728 \| 5.8 \| 32 \| 7 \| \| C_90_H_166_O_16_Na^+^ \| 1526.2068 \| 1526.2055 \| -0.8 \| 29 \| 8 \| \| C_88_H_160_O_18_Na^+^ \| 1528.1497 \| 1528.1462 \| -2.3 \| 26 \| 9 \| \| C_86_H_154_O_20_Na^+^ \| 1530.0926 \| 1530.0837 \| -5.8 \| 23 \| 10 \| \| C_98_H_188_O_10_Na^+^ \| 1548.4095 \| 1548.3978 \| -7.6 \| 39 \| 5 \| \| C_96_H_182_O_12_Na^+^ \| 1550.3523 \| 1550.3495 \| -1.8 \| 36 \| 6 \| \| C_94_H_176_O_14_Na^+^ \| 1552.2952 \| 1552.2981 \| 1.8 \| 33 \| 7 \| \| C_92_H_170_O_16_Na^+^ \| 1554.2381 \| 1554.2467 \| 5.5 \| 30 \| 8 \| \| C_90_H_164_O_18_Na^+^ \| 1556.1810 \| 1556.1746 \| -4.1 \| 27 \| 9 \| \| C_88_H_158_O_20_Na^+^ \| 1558.1239 \| 1558.1185 \| -3.5 \| 24 \| 10 \| \| C_86_H_152_O_22_Na^+^ \| 1560.0667 \| 1560.0559 \| -6.9 \| 21 \| 11 \| \| C_84_H_146_O_24_Na^+^ \| 1562.0096 \| 1562.0093 \| -0.2 \| 18 \| 12 \| \| C_82_H_140_O_26_Na^+^ \| 1563.9525 \| 1563.9531 \| 0.4 \| 15 \| 13 \| \| C_100_H_192_O_10_Na^+^ \| 1576.4408 \| 1576.4341 \| -4.2 \| 40 \| 5 \| \| C_98_H_186_O_12_Na^+^ \| 1578.3836 \| 1578.3780 \| -3.6 \| 37 \| 6 \| \| C_96_H_180_O_14_Na^+^ \| 1580.3265 \| 1580.3265 \| 0.0 \| 34 \| 7 \| \| C_94_H_174_O_16_Na^+^ \| 1582.2694 \| 1582.2783 \| 5.6 \| 31 \| 8 \| \| C_92_H_168_O_18_Na^+^ \| 1584.2123 \| 1584.2142 \| 1.2 \| 28 \| 9 \| \| C_90_H_162_O_20_Na^+^ \| 1586.1552 \| 1586.1501 \| -3.2 \| 25 \| 10 \| \| C_88_H_156_O_22_Na^+^ \| 1588.0980 \| 1588.1034 \| 3.4 \| 22 \| 11 \| \| C_86_H_150_O_24_Na^+^ \| 1590.0409 \| 1590.0409 \| 0.0 \| 19 \| 12 \| \| C_84_H_144_O_26_Na^+^ \| 1591.9838 \| 1591.9727 \| -7.0 \| 16 \| 13 \| \| C_102_H_196_O_10_Na^+^ \| 1604.4721 \| 1604.4721 \| 0.0 \| 41 \| 5 \| \| C_100_H_190_O_12_Na^+^ \| 1606.4149 \| 1606.4144 \| -0.4 \| 38 \| 6 \| |
| --- | --- | --- | --- | --- | --- | --- | --- | --- | --- | --- | --- | --- | --- | --- | --- | --- | --- | --- | --- | --- | --- | --- | --- | --- | --- | --- | --- | --- | --- | --- | --- | --- | --- | --- | --- | --- | --- | --- | --- | --- | --- | --- | --- | --- | --- | --- | --- | --- | --- | --- | --- | --- | --- | --- | --- | --- | --- | --- | --- | --- | --- | --- | --- | --- | --- | --- | --- | --- | --- | --- | --- | --- | --- | --- | --- | --- | --- | --- | --- | --- | --- | --- | --- | --- | --- | --- | --- | --- | --- | --- | --- | --- | --- | --- | --- | --- | --- | --- | --- | --- | --- | --- | --- | --- | --- | --- | --- | --- | --- | --- | --- | --- | --- | --- | --- | --- | --- | --- | --- | --- | --- | --- | --- | --- | --- | --- | --- | --- | --- | --- | --- | --- | --- | --- | --- | --- | --- | --- | --- | --- | --- | --- | --- | --- | --- | --- | --- | --- | --- | --- | --- | --- | --- | --- | --- | --- | --- | --- | --- | --- | --- | --- | --- | --- | --- | --- | --- | --- | --- | --- | --- | --- | --- | --- | --- | --- | --- | --- | --- | --- | --- | --- | --- | --- | --- | --- | --- | --- | --- | --- | --- | --- | --- | --- | --- | --- | --- | --- | --- | --- | --- | --- | --- | --- | --- | --- | --- | --- | --- | --- | --- | --- | --- | --- | --- | --- | --- | --- | --- | --- | --- | --- | --- | --- | --- | --- | --- | --- | --- | --- | --- | --- | --- | --- | --- | --- | --- | --- | --- | --- | --- | --- | --- | --- | --- | --- | --- | --- | --- | --- | --- | --- | --- | --- | --- | --- | --- | --- | --- | --- | --- | --- | --- | --- | --- | --- | --- | --- | --- | --- | --- | --- | --- | --- | --- | --- | --- | --- | --- | --- | --- | --- | --- | --- | --- | --- | --- | --- | --- | --- | --- | --- | --- | --- | --- | --- | --- | --- | --- | --- | --- | --- | --- | --- | --- | --- | --- | --- | --- | --- | --- | --- | --- | --- | --- | --- | --- | --- | --- | --- | --- | --- | --- | --- | --- | --- | --- | --- | --- | --- | --- | --- | --- | --- | --- | --- | --- | --- | --- | --- | --- | --- | --- | --- | --- | --- | --- | --- | --- | --- | --- | --- | --- | --- | --- | --- | --- | --- | --- | --- | --- | --- | --- | --- | --- | --- | --- | --- | --- | --- | --- | --- | --- | --- | --- | --- | --- | --- | --- | --- | --- | --- | --- | --- | --- | --- | --- | --- | --- | --- | --- | --- | --- | --- | --- | --- | --- | --- | --- | --- | --- | --- | --- | --- | --- | --- | --- | --- | --- | --- | --- | --- | --- | --- | --- | --- | --- | --- | --- | --- | --- | --- | --- | --- | --- | --- | --- | --- | --- | --- | --- | --- | --- | --- | --- | --- | --- | --- | --- | --- | --- | --- | --- | --- | --- | --- | --- | --- | --- | --- | --- | --- | --- | --- | --- | --- | --- | --- | --- | --- | --- | --- | --- | --- | --- | --- | --- | --- | --- | --- | --- | --- | --- | --- | --- | --- | --- | --- | --- | --- | --- | --- | --- | --- | --- | --- | --- | --- | --- | --- | --- | --- | --- | --- | --- | --- | --- | --- | --- | --- | --- | --- | --- | --- | --- | --- | --- | --- | --- | --- | --- | --- | --- | --- | --- | --- | --- | --- | --- | --- | --- | --- | --- | --- | --- | --- | --- | --- | --- | --- | --- | --- | --- | --- | --- | --- | --- | --- | --- | --- | --- |

**Table S1** (next)

| \| **Elemental composition** \| **(*m/z*)_th_** \| **(*m/z*)_expe_** \| **Err (ppm)** \| **E** \| **VA** \| \| --- \| --- \| --- \| --- \| --- \| --- \| \| C_98_H_184_O_14_Na^+^ \| 1608.3578 \| 1608.3566 \| -0.8 \| 35 \| 7 \| \| C_96_H_178_O_16_Na^+^ \| 1610.3007 \| 1610.2893 \| -7.1 \| 32 \| 8 \| \| C_94_H_172_O_18_Na^+^ \| 1612.2436 \| 1612.2379 \| -3.5 \| 29 \| 9 \| \| C_92_H_166_O_20_Na^+^ \| 1614.1865 \| 1614.1785 \| -4.9 \| 26 \| 10 \| \| C_90_H_160_O_22_Na^+^ \| 1616.1293 \| 1616.1319 \| 1.6 \| 23 \| 11 \| \| C_104_H_200_O_10_Na^+^ \| 1632.5034 \| 1632.5212 \| 10.9 \| 42 \| 5 \| \| C_102_H_194_O_12_Na^+^ \| 1634.4462 \| 1634.4460 \| -0.2 \| 39 \| 6 \| \| C_100_H_188_O_14_Na^+^ \| 1636.3891 \| 1636.3819 \| -4.4 \| 36 \| 7 \| \| C_98_H_182_O_16_Na^+^ \| 1638.3320 \| 1638.3415 \| 5.8 \| 33 \| 8 \| \| C_92_H_164_O_22_Na^+^ \| 1644.1606 \| 1644.1587 \| -1.2 \| 24 \| 11 \| \| C_90_H_158_O_24_Na^+^ \| 1646.1035 \| 1646.0994 \| -2.5 \| 21 \| 12 \| \| C_96_H_176_O_18_Na^+^ \| 1640.2749 \| 1640.2743 \| -0.4 \| 30 \| 9 \| \| C_94_H_170_O_20_Na^+^ \| 1642.2178 \| 1642.2260 \| 5.0 \| 27 \| 10 \| \| C_104_H_198_O_12_Na^+^ \| 1662.4775 \| 1662.4649 \| -7.6 \| 40 \| 6 \| | \| **Elemental composition** \| **(*m/z*)_th_** \| **(*m/z*)_expe_** \| **Err (ppm)** \| **E** \| **VA** \| \| --- \| --- \| --- \| --- \| --- \| --- \| \| C_102_H_192_O_14_Na^+^ \| 1664.4204 \| 1664.4056 \| -8.9 \| 37 \| 7 \| \| C_100_H_186_O_16_Na^+^ \| 1666.3633 \| 1666.3557 \| -4.5 \| 34 \| 8 \| \| C_98_H_180_O_18_Na^+^ \| 1668.3062 \| 1668.3091 \| 1.7 \| 31 \| 9 \| \| C_96_H_174_O_20_Na^+^ \| 1670.2491 \| 1670.2656 \| 9.9 \| 28 \| 10 \| \| C_94_H_168_O_22_Na^+^ \| 1672.1919 \| 1672.1935 \| 1.0 \| 25 \| 11 \| \| C_92_H_162_O_24_Na^+^ \| 1674.1348 \| 1674.1310 \| -2.3 \| 22 \| 12 \| \| C_106_H_202_O_12_Na^+^ \| 1690.5088 \| 1690.5061 \| -1.6 \| 41 \| 6 \| \| C_104_H_196_O_14_Na^+^ \| 1692.4517 \| 1692.4467 \| -3.0 \| 38 \| 7 \| \| C_102_H_190_O_16_Na^+^ \| 1694.3946 \| 1694.4064 \| 7.0 \| 35 \| 8 \| \| C_100_H_184_O_18_Na^+^ \| 1696.3375 \| 1696.3264 \| -6.5 \| 32 \| 9 \| \| C_98_H_178_O_20_Na^+^ \| 1698.2804 \| 1698.2766 \| -2.2 \| 29 \| 10 \| \| C_96_H_172_O_22_Na^+^ \| 1700.2232 \| 1700.2283 \| 3.0 \| 26 \| 11 \| \|  \|  \|  \|  \|  \|  \| |
| --- | --- | --- | --- | --- | --- | --- | --- | --- | --- | --- | --- | --- | --- | --- | --- | --- | --- | --- | --- | --- | --- | --- | --- | --- | --- | --- | --- | --- | --- | --- | --- | --- | --- | --- | --- | --- | --- | --- | --- | --- | --- | --- | --- | --- | --- | --- | --- | --- | --- | --- | --- | --- | --- | --- | --- | --- | --- | --- | --- | --- | --- | --- | --- | --- | --- | --- | --- | --- | --- | --- | --- | --- | --- | --- | --- | --- | --- | --- | --- | --- | --- | --- | --- | --- | --- | --- | --- | --- | --- | --- | --- | --- | --- | --- | --- | --- | --- | --- | --- | --- | --- | --- | --- | --- | --- | --- | --- | --- | --- | --- | --- | --- | --- | --- | --- | --- | --- | --- | --- | --- | --- | --- | --- | --- | --- | --- | --- | --- | --- | --- | --- | --- | --- | --- | --- | --- | --- | --- | --- | --- | --- | --- | --- | --- | --- | --- | --- | --- | --- | --- | --- | --- | --- | --- | --- | --- | --- | --- | --- | --- | --- | --- | --- | --- | --- | --- | --- | --- | --- | --- | --- | --- | --- | --- | --- |


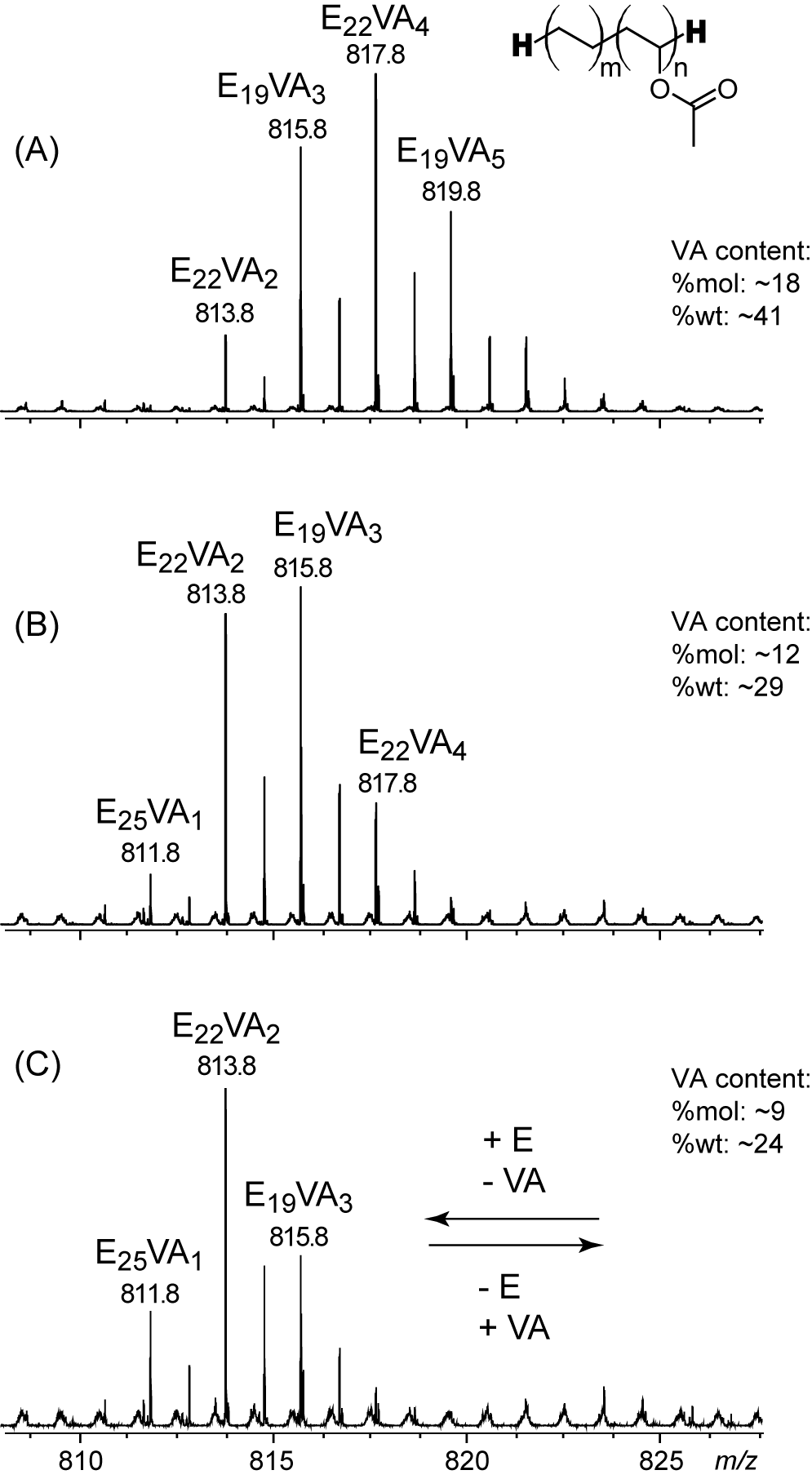


**Figure S3.** Restricted MALDI-MS spectra of the fractions #3 of (A) EVA40, (B) EVA25 and (C) EVA18. Composition in terms of E units m and VA units n are mentioned for each major peak as E_m_VA_n_ considering hydrogen atoms as end-groups.

Content of VA is calculated based on the m and n values convoluted by the relative intensity of each peak (applying this procedure to a unique pattern or to the whole mass spectrum provides similar results).

EVA40: %VA (%wt) ~ 41

EVA25: %VA (%wt) ~ 29

EVA18: %VA (%wt) ~ 24


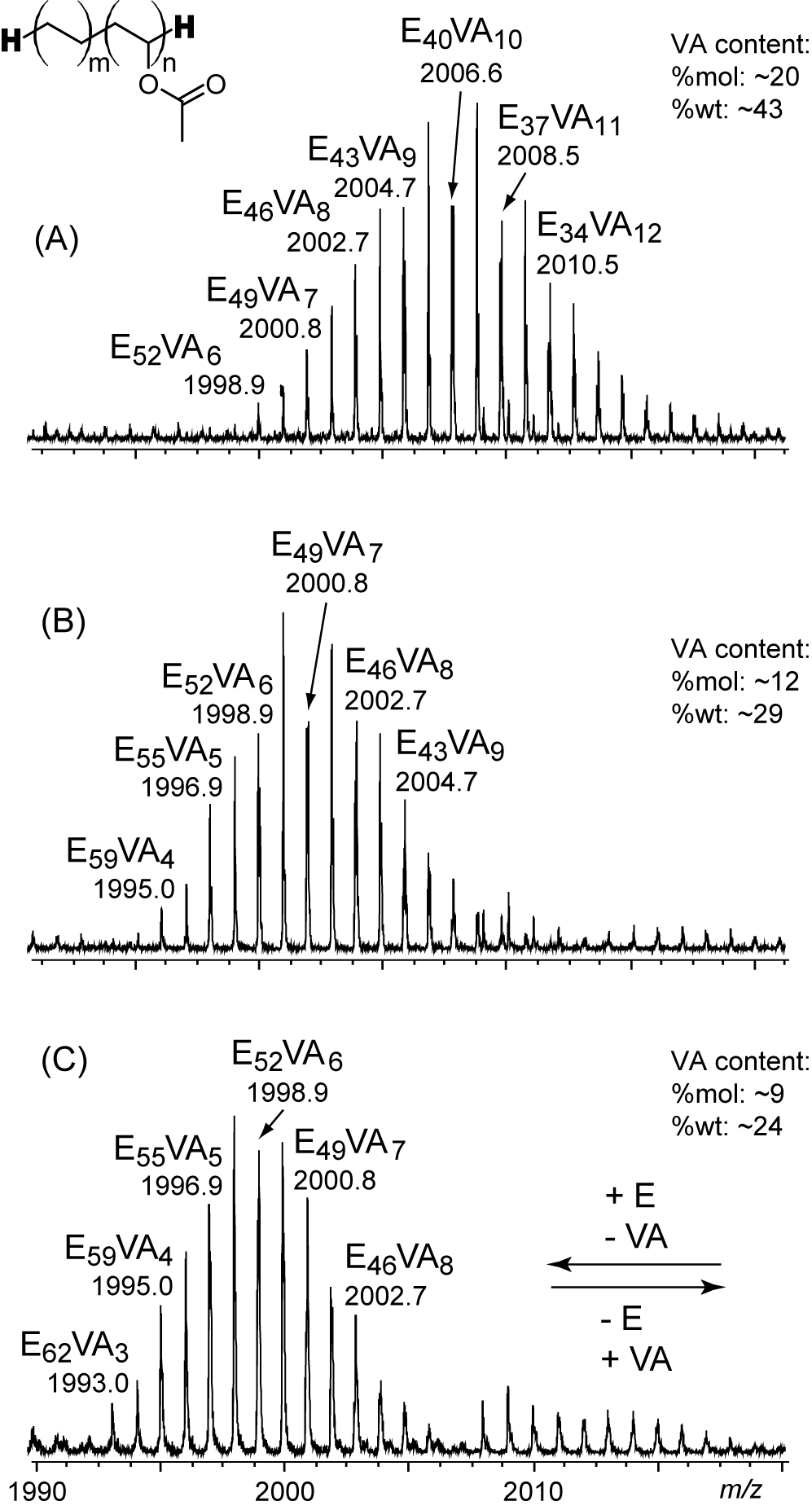


**Figure S4.** Restricted MALDI-MS spectra of the fractions #3 of (A) EVA40, (B) EVA25 and (C) EVA18. Composition in terms of E units m and VA units n are mentioned for each major peak as E_m_VA_n_ considering hydrogen atoms as end-groups.

Content of VA is calculated based on the m and n values convoluted by the relative intensity of each peak (applying this procedure to a unique pattern or to the whole mass spectrum provides similar results).

EVA40: %VA (%wt) ~ 43

EVA25: %VA (%wt) ~ 29

EVA18: %VA (%wt) ~ 24

**Evaluation of the E and VA compositions from KMD.**

Starting from the definition of the mass of a given E_m_VA_n_ oligomer (with α and ω end-groups):

| $m/z(oligomer)=m\left( \alpha)+m(\omega)+m/z(cation \right)+m\left( E \right)\cdot m+m\left( VA \right)\cdot n$ | (1) |
| --- | --- |

The Kendrick mass is calculated as follows:

| $KM(oligomer)=KM\left( \alpha+\omega+cation \right)+KM\left( E \right)\cdot m+KM\left( VA \right)\cdot n$ | (2) |
| --- | --- |

The nominal Kendrick mass is the rounded value of KM:

| $NKM\left( oligomer \right)=NKM\left( \alpha+\omega+cation \right)+NKM\left( E \right)\cdot m+NKM\left( VA \right)\cdot n$ | (3) |
| --- | --- |

And the Kendrick mass defect KMD is calculated as follows:

| $KMD\left( oligomer \right)=NKM\left( oligomer \right)-KM(oligomer)$ | (4) |
| --- | --- |

Replacing KM(oligomer) by Eq. 2 and NKM(oligomer) by Eq. 3 in Eq. 4:

| $KMD\left( oligomer \right)= NKM\left( \alpha+\omega+cation \right)+NKM\left( E \right)\cdot m+NKM\left( VA \right)\cdot n-(KM\left( \alpha+\omega+cation \right)+KM\left( E \right)\cdot m+KM\left( VA \right)\cdot n)$ | (5) |
| --- | --- |

Which could be re-written as follows:

| $KMD\left( oligomer \right)= NKM\left( \alpha+\omega+cation \right)- KM\left( \alpha+\omega+cation \right)+NKM\left( E \right)\cdot m-KM\left( E \right)\cdot m+NKM\left( VA \right)\cdot n-KM\left( VA \right)\cdot n$ | (6) |
| --- | --- |

With:

| $NKM\left( \alpha+\omega+cation \right)- KM\left( \alpha+\omega+cation \right)=KMD\left( \alpha+\omega+cation \right)$ | | (7) |
| --- | --- | --- |
| $NKM\left( VA \right)\cdot n-KM\left( VA \right)\cdot n=KMD\left( VA \right)\cdot n$ | (8) | |

Considering E as the base unit, KM(E)=NKM(E)=28

| $NKM\left( E \right)\cdot m-KM\left( E \right)\cdot m=KMD\left( E \right)\cdot m=0$ | (9) |
| --- | --- |

Eq. 6 thus simplifies in:

| $KMD\left( oligomer \right)= KMD\left( \alpha+\omega+cation \right)+KMD\left( VA \right)\cdot n$ | (10) |
| --- | --- |

Eq.3 and Eq. 10 could be used to calculate the number m of E units and the number n of VA units of a given co-oligomer using its NKM and KMD (calculated from the accurate mass measurement).

With m and n as the unknowns, Eq. 10 and Eq. 3 are re-written as follows:

| $n={(KMD\left( oligomer \right)-KMD\left( \alpha+\omega+cation \right))}/{KMD(VA)}$ | (11) |
| --- | --- |
| $m={(NKM\left( oligomer \right)-NKM\left( \alpha+\omega+cation \right)-NKM\left( VA \right)\cdot n)}/{NKM(E)}$ | (12) |

Considering α=ω=H, cation=Na^+^, Eq. 13 and Eq. 14 are re-written as follows:

| $n={(KMD\left( oligomer \right)-0.022501)}/{0.059289}$ | (13) |
| --- | --- |
| $m={(NKM\left( oligomer \right)-25-86\cdot n)}/{28}$ | (14) |

In particular at the centroid of the KMD plot, the average composition in E and VA from which the VA content (%wt) is derived is calculated as follows:

| $n_{centroid}={(KMD\left( centroid \right)-0.022501)}/{0.059289}$ | (15) |
| --- | --- |
| $m_{centroid}={(NKM\left( centroid \right)-25-86\cdot n_{centroid})}/{28}$ | (16) |

**Table S2.** VA content (mol% and wt%) calculated from the accurate mass measurements and associated compositions in E and VA units from the MALDI mass spectrum of EVA40 (fraction #2).

| \| ***m/z*** \| **ab.**  **(%)** \| **VA** \| **E** \| **VA*ab.** \| **E*ab.** \| \| --- \| --- \| --- \| --- \| --- \| --- \| \| 871.7722 \| 24 \| 3 \| 21 \| 71 \| 500 \| \| 873.7156 \| 30 \| 4 \| 18 \| 119 \| 535 \| \| 875.6587 \| 17 \| 5 \| 15 \| 83 \| 248 \| \| 877.6020 \| 6 \| 6 \| 12 \| 36 \| 72 \| \| 899.8031 \| 31 \| 3 \| 22 \| 94 \| 690 \| \| 901.7461 \| 39 \| 4 \| 19 \| 158 \| 750 \| \| 903.6891 \| 27 \| 5 \| 16 \| 136 \| 436 \| \| 905.6317 \| 11 \| 6 \| 13 \| 64 \| 138 \| \| 925.8917 \| 9 \| 2 \| 26 \| 17 \| 223 \| \| 927.8351 \| 33 \| 3 \| 23 \| 98 \| 754 \| \| 929.7784 \| 51 \| 4 \| 20 \| 204 \| 1021 \| \| 931.7204 \| 39 \| 5 \| 17 \| 193 \| 657 \| \| 933.6620 \| 18 \| 6 \| 14 \| 109 \| 254 \| \| 953.9230 \| 8 \| 2 \| 27 \| 17 \| 227 \| \| 955.8667 \| 38 \| 3 \| 24 \| 113 \| 907 \| \| 957.8092 \| 67 \| 4 \| 21 \| 266 \| 1399 \| \| 959.7526 \| 52 \| 5 \| 18 \| 262 \| 943 \| \| 961.6944 \| 22 \| 6 \| 15 \| 132 \| 329 \| \| 963.6378 \| 9 \| 7 \| 12 \| 60 \| 103 \| \| 981.9551 \| 7 \| 2 \| 28 \| 15 \| 205 \| \| 983.8973 \| 36 \| 3 \| 25 \| 107 \| 896 \| \| 985.8406 \| 72 \| 4 \| 22 \| 290 \| 1593 \| \| 987.7831 \| 65 \| 5 \| 19 \| 325 \| 1234 \| \| 989.7260 \| 33 \| 6 \| 16 \| 199 \| 530 \| \| 991.6686 \| 13 \| 7 \| 13 \| 89 \| 166 \| \| 1011.9284 \| 34 \| 3 \| 26 \| 103 \| 894 \| \| 1013.8725 \| 80 \| 4 \| 23 \| 319 \| 1836 \| \| 1015.8153 \| 79 \| 5 \| 20 \| 394 \| 1576 \| \| 1017.7574 \| 45 \| 6 \| 17 \| 268 \| 759 \| \| 1019.6996 \| 16 \| 7 \| 14 \| 109 \| 219 \| \| 1039.9604 \| 29 \| 3 \| 27 \| 87 \| 781 \| \| 1041.9034 \| 70 \| 4 \| 24 \| 279 \| 1675 \| \| 1043.8460 \| 86 \| 5 \| 21 \| 430 \| 1807 \| \| 1045.7893 \| 56 \| 6 \| 18 \| 338 \| 1013 \| \| 1047.7318 \| 23 \| 7 \| 15 \| 159 \| 341 \| \| 1049.6758 \| 9 \| 8 \| 12 \| 74 \| 111 \| \| 1067.9920 \| 29 \| 3 \| 28 \| 86 \| 802 \| \| 1069.9342 \| 76 \| 4 \| 25 \| 303 \| 1895 \| | \| ***m/z*** \| **ab.**  **(%)** \| **VA** \| **E** \| **VA*ab.** \| **E*ab.** \| \| --- \| --- \| --- \| --- \| --- \| --- \| \| 1071.8775 \| 94 \| 5 \| 22 \| 471 \| 2071 \| \| 1073.8201 \| 67 \| 6 \| 19 \| 400 \| 1267 \| \| 1075.7632 \| 27 \| 7 \| 16 \| 191 \| 436 \| \| 1077.7064 \| 12 \| 8 \| 13 \| 99 \| 160 \| \| 1096.0224 \| 22 \| 3 \| 29 \| 67 \| 650 \| \| 1097.9660 \| 74 \| 4 \| 26 \| 296 \| 1922 \| \| 1099.9083 \| 96 \| 5 \| 23 \| 478 \| 2197 \| \| 1101.8516 \| 70 \| 6 \| 20 \| 418 \| 1392 \| \| 1103.7948 \| 35 \| 7 \| 17 \| 242 \| 588 \| \| 1105.7371 \| 18 \| 8 \| 14 \| 145 \| 253 \| \| 1124.0540 \| 20 \| 3 \| 30 \| 61 \| 606 \| \| 1125.9969 \| 63 \| 4 \| 27 \| 250 \| 1689 \| \| 1127.9406 \| 100 \| 5 \| 24 \| 500 \| 2400 \| \| 1129.8830 \| 85 \| 6 \| 21 \| 508 \| 1778 \| \| 1131.8260 \| 41 \| 7 \| 18 \| 289 \| 744 \| \| 1133.7685 \| 22 \| 8 \| 15 \| 175 \| 329 \| \| 1152.0861 \| 19 \| 3 \| 31 \| 56 \| 574 \| \| 1154.0281 \| 57 \| 4 \| 28 \| 229 \| 1604 \| \| 1155.9710 \| 100 \| 5 \| 25 \| 500 \| 2498 \| \| 1157.9137 \| 88 \| 6 \| 22 \| 526 \| 1929 \| \| 1159.8572 \| 42 \| 7 \| 19 \| 296 \| 802 \| \| 1161.7993 \| 23 \| 8 \| 16 \| 186 \| 372 \| \| 1180.1168 \| 17 \| 3 \| 32 \| 50 \| 530 \| \| 1182.0589 \| 50 \| 4 \| 29 \| 201 \| 1456 \| \| 1184.0030 \| 91 \| 5 \| 26 \| 455 \| 2367 \| \| 1185.9461 \| 87 \| 6 \| 23 \| 523 \| 2005 \| \| 1187.8886 \| 52 \| 7 \| 20 \| 362 \| 1033 \| \| 1189.8312 \| 26 \| 8 \| 17 \| 205 \| 436 \| \| 1191.7737 \| 11 \| 9 \| 14 \| 100 \| 156 \| \| 1208.1479 \| 12 \| 3 \| 33 \| 35 \| 386 \| \| 1210.0904 \| 46 \| 4 \| 30 \| 184 \| 1381 \| \| 1212.0339 \| 86 \| 5 \| 27 \| 430 \| 2323 \| \| 1213.9766 \| 94 \| 6 \| 24 \| 562 \| 2246 \| \| 1215.9196 \| 57 \| 7 \| 21 \| 400 \| 1199 \| \| 1217.8618 \| 26 \| 8 \| 18 \| 212 \| 476 \| \| 1219.8052 \| 16 \| 9 \| 15 \| 140 \| 233 \| \| 1236.1780 \| 10 \| 3 \| 34 \| 31 \| 350 \| \| 1238.1214 \| 37 \| 4 \| 31 \| 147 \| 1139 \| |
| --- | --- | --- | --- | --- | --- | --- | --- | --- | --- | --- | --- | --- | --- | --- | --- | --- | --- | --- | --- | --- | --- | --- | --- | --- | --- | --- | --- | --- | --- | --- | --- | --- | --- | --- | --- | --- | --- | --- | --- | --- | --- | --- | --- | --- | --- | --- | --- | --- | --- | --- | --- | --- | --- | --- | --- | --- | --- | --- | --- | --- | --- | --- | --- | --- | --- | --- | --- | --- | --- | --- | --- | --- | --- | --- | --- | --- | --- | --- | --- | --- | --- | --- | --- | --- | --- | --- | --- | --- | --- | --- | --- | --- | --- | --- | --- | --- | --- | --- | --- | --- | --- | --- | --- | --- | --- | --- | --- | --- | --- | --- | --- | --- | --- | --- | --- | --- | --- | --- | --- | --- | --- | --- | --- | --- | --- | --- | --- | --- | --- | --- | --- | --- | --- | --- | --- | --- | --- | --- | --- | --- | --- | --- | --- | --- | --- | --- | --- | --- | --- | --- | --- | --- | --- | --- | --- | --- | --- | --- | --- | --- | --- | --- | --- | --- | --- | --- | --- | --- | --- | --- | --- | --- | --- | --- | --- | --- | --- | --- | --- | --- | --- | --- | --- | --- | --- | --- | --- | --- | --- | --- | --- | --- | --- | --- | --- | --- | --- | --- | --- | --- | --- | --- | --- | --- | --- | --- | --- | --- | --- | --- | --- | --- | --- | --- | --- | --- | --- | --- | --- | --- | --- | --- | --- | --- | --- | --- | --- | --- | --- | --- | --- | --- | --- | --- | --- | --- | --- | --- | --- | --- | --- | --- | --- | --- | --- | --- | --- | --- | --- | --- | --- | --- | --- | --- | --- | --- | --- | --- | --- | --- | --- | --- | --- | --- | --- | --- | --- | --- | --- | --- | --- | --- | --- | --- | --- | --- | --- | --- | --- | --- | --- | --- | --- | --- | --- | --- | --- | --- | --- | --- | --- | --- | --- | --- | --- | --- | --- | --- | --- | --- | --- | --- | --- | --- | --- | --- | --- | --- | --- | --- | --- | --- | --- | --- | --- | --- | --- | --- | --- | --- | --- | --- | --- | --- | --- | --- | --- | --- | --- | --- | --- | --- | --- | --- | --- | --- | --- | --- | --- | --- | --- | --- | --- | --- | --- | --- | --- | --- | --- | --- | --- | --- | --- | --- | --- | --- | --- | --- | --- | --- | --- | --- | --- | --- | --- | --- | --- | --- | --- | --- | --- | --- | --- | --- | --- | --- | --- | --- | --- | --- | --- | --- | --- | --- | --- | --- | --- | --- | --- | --- | --- | --- | --- | --- | --- | --- | --- | --- | --- | --- | --- | --- | --- | --- | --- | --- | --- | --- | --- | --- | --- | --- | --- | --- | --- | --- | --- | --- | --- | --- | --- | --- | --- | --- | --- | --- | --- | --- | --- | --- | --- | --- | --- | --- | --- | --- | --- | --- | --- | --- | --- | --- | --- | --- | --- | --- | --- | --- | --- | --- | --- | --- | --- | --- | --- | --- | --- | --- | --- | --- | --- | --- | --- | --- | --- | --- | --- | --- | --- |

**Table S2.** (next)

| \| ***m/z*** \| **ab.**  **(%)** \| **VA** \| **E** \| **VA*ab.** \| **E*ab.** \| \| --- \| --- \| --- \| --- \| --- \| --- \| \| 1240.0651 \| 80 \| 5 \| 28 \| 402 \| 2251 \| \| 1242.0081 \| 93 \| 6 \| 25 \| 556 \| 2316 \| \| 1243.9496 \| 61 \| 7 \| 22 \| 426 \| 1339 \| \| 1245.8935 \| 33 \| 8 \| 19 \| 264 \| 628 \| \| 1247.8359 \| 15 \| 9 \| 16 \| 135 \| 241 \| \| 1266.1576 \| 28 \| 4 \| 32 \| 113 \| 902 \| \| 1268.0962 \| 71 \| 5 \| 29 \| 355 \| 2061 \| \| 1270.0388 \| 80 \| 6 \| 26 \| 479 \| 2078 \| \| 1271.9819 \| 61 \| 7 \| 23 \| 430 \| 1412 \| \| 1273.9253 \| 32 \| 8 \| 20 \| 254 \| 636 \| \| 1275.8691 \| 16 \| 9 \| 17 \| 148 \| 280 \| \| 1277.8114 \| 10 \| 10 \| 14 \| 100 \| 140 \| \| 1294.1876 \| 24 \| 4 \| 33 \| 97 \| 798 \| \| 1296.1274 \| 61 \| 5 \| 30 \| 306 \| 1839 \| \| 1298.0701 \| 80 \| 6 \| 27 \| 482 \| 2171 \| \| 1300.0125 \| 60 \| 7 \| 24 \| 417 \| 1429 \| \| 1301.9558 \| 34 \| 8 \| 21 \| 271 \| 713 \| \| 1303.8981 \| 22 \| 9 \| 18 \| 200 \| 399 \| \| 1305.8411 \| 11 \| 10 \| 15 \| 109 \| 163 \| \| 1322.2251 \| 16 \| 4 \| 34 \| 62 \| 530 \| \| 1324.1619 \| 48 \| 5 \| 31 \| 238 \| 1474 \| \| 1326.0975 \| 72 \| 6 \| 28 \| 431 \| 2012 \| \| 1328.0463 \| 62 \| 7 \| 25 \| 433 \| 1547 \| \| 1329.9930 \| 34 \| 8 \| 22 \| 269 \| 740 \| \| 1331.9420 \| 13 \| 9 \| 19 \| 115 \| 244 \| \| 1333.8792 \| 9 \| 10 \| 16 \| 91 \| 145 \| \| 1350.2523 \| 15 \| 4 \| 35 \| 61 \| 537 \| \| 1352.1789 \| 30 \| 5 \| 32 \| 152 \| 976 \| \| 1354.1361 \| 64 \| 6 \| 29 \| 387 \| 1869 \| \| 1356.0853 \| 46 \| 7 \| 26 \| 321 \| 1192 \| \| 1358.0263 \| 32 \| 8 \| 23 \| 254 \| 731 \| \| 1359.9582 \| 20 \| 9 \| 20 \| 179 \| 398 \| \| 1361.9060 \| 11 \| 10 \| 17 \| 114 \| 193 \| \| 1378.2785 \| 14 \| 4 \| 36 \| 54 \| 489 \| \| 1380.2199 \| 34 \| 5 \| 33 \| 172 \| 1133 \| \| 1382.1592 \| 56 \| 6 \| 30 \| 337 \| 1684 \| \| 1384.1038 \| 54 \| 7 \| 27 \| 377 \| 1453 \| \| 1386.0463 \| 37 \| 8 \| 24 \| 300 \| 900 \| | \| ***m/z*** \| **ab.**  **(%)** \| **VA** \| **E** \| **VA*ab.** \| **E*ab.** \| \| --- \| --- \| --- \| --- \| --- \| --- \| \| 1387.9908 \| 21 \| 9 \| 21 \| 188 \| 439 \| \| 1389.9354 \| 12 \| 10 \| 18 \| 118 \| 212 \| \| 1391.8778 \| 7 \| 11 \| 15 \| 77 \| 105 \| \| 1406.3101 \| 10 \| 4 \| 37 \| 39 \| 360 \| \| 1408.2599 \| 22 \| 5 \| 34 \| 111 \| 756 \| \| 1410.1887 \| 44 \| 6 \| 31 \| 264 \| 1363 \| \| 1412.1417 \| 54 \| 7 \| 28 \| 375 \| 1499 \| \| 1414.0862 \| 36 \| 8 \| 25 \| 286 \| 894 \| \| 1416.0224 \| 20 \| 9 \| 22 \| 180 \| 440 \| \| 1417.9669 \| 12 \| 10 \| 19 \| 125 \| 237 \| \| 1419.9125 \| 6 \| 11 \| 16 \| 67 \| 98 \| \| 1434.3289 \| 6 \| 4 \| 38 \| 25 \| 239 \| \| 1436.2872 \| 19 \| 5 \| 35 \| 97 \| 679 \| \| 1438.2318 \| 37 \| 6 \| 32 \| 220 \| 1174 \| \| 1440.1658 \| 48 \| 7 \| 29 \| 339 \| 1406 \| \| 1442.1104 \| 38 \| 8 \| 26 \| 304 \| 987 \| \| 1444.0549 \| 20 \| 9 \| 23 \| 177 \| 453 \| \| 1446.0027 \| 12 \| 10 \| 20 \| 118 \| 235 \| \| 1447.9398 \| 8 \| 11 \| 17 \| 84 \| 130 \| \| 1464.3198 \| 16 \| 5 \| 36 \| 81 \| 584 \| \| 1466.2644 \| 32 \| 6 \| 33 \| 195 \| 1071 \| \| 1468.1963 \| 42 \| 7 \| 30 \| 296 \| 1269 \| \| 1470.1461 \| 32 \| 8 \| 27 \| 257 \| 867 \| \| 1472.0801 \| 18 \| 9 \| 24 \| 159 \| 424 \| \| 1474.0353 \| 12 \| 10 \| 21 \| 115 \| 242 \| \| 1475.9724 \| 9 \| 11 \| 18 \| 98 \| 160 \| \| 1477.9202 \| 4 \| 12 \| 15 \| 45 \| 56 \| \| 1492.3547 \| 12 \| 5 \| 37 \| 60 \| 448 \| \| 1494.2907 \| 28 \| 6 \| 34 \| 168 \| 949 \| \| 1496.2369 \| 32 \| 7 \| 31 \| 223 \| 988 \| \| 1498.1728 \| 32 \| 8 \| 28 \| 256 \| 895 \| \| 1500.1122 \| 19 \| 9 \| 25 \| 172 \| 476 \| \| 1502.0619 \| 12 \| 10 \| 22 \| 123 \| 271 \| \| 1504.0006 \| 8 \| 11 \| 19 \| 89 \| 154 \| \| 1520.3788 \| 11 \| 5 \| 38 \| 56 \| 424 \| \| 1522.3369 \| 15 \| 6 \| 35 \| 89 \| 518 \| \| 1524.2728 \| 24 \| 7 \| 32 \| 170 \| 776 \| \| 1526.2055 \| 27 \| 8 \| 29 \| 219 \| 795 \| |
| --- | --- | --- | --- | --- | --- | --- | --- | --- | --- | --- | --- | --- | --- | --- | --- | --- | --- | --- | --- | --- | --- | --- | --- | --- | --- | --- | --- | --- | --- | --- | --- | --- | --- | --- | --- | --- | --- | --- | --- | --- | --- | --- | --- | --- | --- | --- | --- | --- | --- | --- | --- | --- | --- | --- | --- | --- | --- | --- | --- | --- | --- | --- | --- | --- | --- | --- | --- | --- | --- | --- | --- | --- | --- | --- | --- | --- | --- | --- | --- | --- | --- | --- | --- | --- | --- | --- | --- | --- | --- | --- | --- | --- | --- | --- | --- | --- | --- | --- | --- | --- | --- | --- | --- | --- | --- | --- | --- | --- | --- | --- | --- | --- | --- | --- | --- | --- | --- | --- | --- | --- | --- | --- | --- | --- | --- | --- | --- | --- | --- | --- | --- | --- | --- | --- | --- | --- | --- | --- | --- | --- | --- | --- | --- | --- | --- | --- | --- | --- | --- | --- | --- | --- | --- | --- | --- | --- | --- | --- | --- | --- | --- | --- | --- | --- | --- | --- | --- | --- | --- | --- | --- | --- | --- | --- | --- | --- | --- | --- | --- | --- | --- | --- | --- | --- | --- | --- | --- | --- | --- | --- | --- | --- | --- | --- | --- | --- | --- | --- | --- | --- | --- | --- | --- | --- | --- | --- | --- | --- | --- | --- | --- | --- | --- | --- | --- | --- | --- | --- | --- | --- | --- | --- | --- | --- | --- | --- | --- | --- | --- | --- | --- | --- | --- | --- | --- | --- | --- | --- | --- | --- | --- | --- | --- | --- | --- | --- | --- | --- | --- | --- | --- | --- | --- | --- | --- | --- | --- | --- | --- | --- | --- | --- | --- | --- | --- | --- | --- | --- | --- | --- | --- | --- | --- | --- | --- | --- | --- | --- | --- | --- | --- | --- | --- | --- | --- | --- | --- | --- | --- | --- | --- | --- | --- | --- | --- | --- | --- | --- | --- | --- | --- | --- | --- | --- | --- | --- | --- | --- | --- | --- | --- | --- | --- | --- | --- | --- | --- | --- | --- | --- | --- | --- | --- | --- | --- | --- | --- | --- | --- | --- | --- | --- | --- | --- | --- | --- | --- | --- | --- | --- | --- | --- | --- | --- | --- | --- | --- | --- | --- | --- | --- | --- | --- | --- | --- | --- | --- | --- | --- | --- | --- | --- | --- | --- | --- | --- | --- | --- | --- | --- | --- | --- | --- | --- | --- | --- | --- | --- | --- | --- | --- | --- | --- | --- | --- | --- | --- | --- | --- | --- | --- | --- | --- | --- | --- | --- | --- | --- | --- | --- | --- | --- | --- | --- | --- | --- | --- | --- | --- | --- | --- | --- | --- | --- | --- | --- | --- | --- | --- | --- | --- | --- | --- | --- | --- | --- | --- | --- | --- | --- | --- | --- | --- | --- | --- | --- | --- | --- | --- | --- | --- | --- | --- | --- | --- | --- | --- | --- | --- | --- | --- | --- | --- | --- | --- | --- | --- | --- | --- | --- | --- | --- | --- | --- | --- | --- | --- | --- | --- |

**Table S2.** (next)

| \| ***m/z*** \| **ab.**  **(%)** \| **VA** \| **E** \| **VA*ab.** \| **E*ab.** \| \| --- \| --- \| --- \| --- \| --- \| --- \| \| 1528.1462 \| 19 \| 9 \| 26 \| 172 \| 497 \| \| 1530.0837 \| 11 \| 10 \| 23 \| 106 \| 243 \| \| 1548.3978 \| 7 \| 5 \| 39 \| 33 \| 255 \| \| 1550.3495 \| 17 \| 6 \| 36 \| 105 \| 628 \| \| 1552.2981 \| 26 \| 7 \| 33 \| 181 \| 853 \| \| 1554.2467 \| 21 \| 8 \| 30 \| 170 \| 638 \| \| 1556.1746 \| 17 \| 9 \| 27 \| 152 \| 456 \| \| 1558.1185 \| 10 \| 10 \| 24 \| 102 \| 245 \| \| 1560.0559 \| 8 \| 11 \| 21 \| 84 \| 160 \| \| 1562.0093 \| 4 \| 12 \| 18 \| 43 \| 65 \| \| 1563.9531 \| 2 \| 13 \| 15 \| 29 \| 33 \| \| 1576.4341 \| 6 \| 5 \| 40 \| 29 \| 229 \| \| 1578.3780 \| 14 \| 6 \| 37 \| 82 \| 504 \| \| 1580.3265 \| 21 \| 7 \| 34 \| 145 \| 705 \| \| 1582.2783 \| 20 \| 8 \| 31 \| 160 \| 619 \| \| 1584.2142 \| 16 \| 9 \| 28 \| 143 \| 444 \| \| 1586.1501 \| 10 \| 10 \| 25 \| 101 \| 253 \| \| 1588.1034 \| 6 \| 11 \| 22 \| 66 \| 133 \| \| 1590.0409 \| 6 \| 12 \| 19 \| 67 \| 107 \| \| 1591.9727 \| 2 \| 13 \| 16 \| 22 \| 27 \| \| 1604.4721 \| 4 \| 5 \| 41 \| 21 \| 171 \| \| 1606.4144 \| 12 \| 6 \| 38 \| 69 \| 438 \| \| 1608.3566 \| 19 \| 7 \| 35 \| 131 \| 657 \| \| 1610.2893 \| 15 \| 8 \| 32 \| 121 \| 484 \| \| 1612.2379 \| 14 \| 9 \| 29 \| 123 \| 397 \| \| 1614.1785 \| 10 \| 10 \| 26 \| 96 \| 250 \| \| 1616.1319 \| 6 \| 11 \| 23 \| 71 \| 149 \| \| 1632.5212 \| 4 \| 5 \| 42 \| 18 \| 152 \| \| 1634.4460 \| 10 \| 6 \| 39 \| 58 \| 374 \| \| 1636.3819 \| 16 \| 7 \| 36 \| 110 \| 567 \| \| 1638.3415 \| 14 \| 8 \| 33 \| 112 \| 463 \| \| 1640.2743 \| 14 \| 9 \| 30 \| 130 \| 432 \| \| 1642.2260 \| 8 \| 10 \| 27 \| 84 \| 227 \| \| 1644.1587 \| 6 \| 11 \| 24 \| 66 \| 145 \| \| 1646.0994 \| 4 \| 12 \| 21 \| 49 \| 86 \| \| 1662.4649 \| 5 \| 6 \| 40 \| 32 \| 215 \| \| 1664.4056 \| 10 \| 7 \| 37 \| 67 \| 354 \| \| 1666.3557 \| 13 \| 8 \| 34 \| 106 \| 452 \| | \| ***m/z*** \| **ab.**  **(%)** \| **VA** \| **E** \| **VA*ab.** \| **E*ab.** \| \| --- \| --- \| --- \| --- \| --- \| --- \| \| 1668.3091 \| 11 \| 9 \| 31 \| 97 \| 335 \| \| 1670.2656 \| 6 \| 10 \| 28 \| 57 \| 160 \| \| 1672.1935 \| 6 \| 11 \| 25 \| 70 \| 159 \| \| 1674.1310 \| 4 \| 12 \| 22 \| 48 \| 89 \| \| 1690.5061 \| 5 \| 6 \| 41 \| 33 \| 222 \| \| 1692.4467 \| 9 \| 7 \| 38 \| 64 \| 349 \| \| 1694.4064 \| 11 \| 8 \| 35 \| 85 \| 372 \| \| 1696.3264 \| 11 \| 9 \| 32 \| 97 \| 346 \| \| 1698.2766 \| 8 \| 10 \| 29 \| 78 \| 226 \| \| 1700.2283 \| 4 \| 11 \| 26 \| 49 \| 116 \| \|  \|  \|  \|  \| **VAav** \| **Eav** \| \|  \|  \|  \|  \| 6.0 \| 24.6 \| \|  \|  \| %VA (mol) \| \| 0.20 \|  \| \|  \|  \| **%VA (wt)** \| \| **0.43** \|  \| \|  \|  \|  \|  \|  \|  \| \|  \|  \|  \|  \|  \|  \| \|  \|  \|  \|  \|  \|  \| \|  \|  \|  \|  \|  \|  \| \|  \|  \|  \|  \|  \|  \| \|  \|  \|  \|  \|  \|  \| \|  \|  \|  \|  \|  \|  \| \|  \|  \|  \|  \|  \|  \| \|  \|  \|  \|  \|  \|  \| \|  \|  \|  \|  \|  \|  \| \|  \|  \|  \|  \|  \|  \| \|  \|  \|  \|  \|  \|  \| \|  \|  \|  \|  \|  \|  \| \|  \|  \|  \|  \|  \|  \| \|  \|  \|  \|  \|  \|  \| \|  \|  \|  \|  \|  \|  \| \|  \|  \|  \|  \|  \|  \| \|  \|  \|  \|  \|  \|  \| \|  \|  \|  \|  \|  \|  \| \|  \|  \|  \|  \|  \|  \| \|  \|  \|  \|  \|  \|  \| \|  \|  \|  \|  \|  \|  \| \|  \|  \|  \|  \|  \|  \| \|  \|  \|  \|  \|  \|  \| |
| --- | --- | --- | --- | --- | --- | --- | --- | --- | --- | --- | --- | --- | --- | --- | --- | --- | --- | --- | --- | --- | --- | --- | --- | --- | --- | --- | --- | --- | --- | --- | --- | --- | --- | --- | --- | --- | --- | --- | --- | --- | --- | --- | --- | --- | --- | --- | --- | --- | --- | --- | --- | --- | --- | --- | --- | --- | --- | --- | --- | --- | --- | --- | --- | --- | --- | --- | --- | --- | --- | --- | --- | --- | --- | --- | --- | --- | --- | --- | --- | --- | --- | --- | --- | --- | --- | --- | --- | --- | --- | --- | --- | --- | --- | --- | --- | --- | --- | --- | --- | --- | --- | --- | --- | --- | --- | --- | --- | --- | --- | --- | --- | --- | --- | --- | --- | --- | --- | --- | --- | --- | --- | --- | --- | --- | --- | --- | --- | --- | --- | --- | --- | --- | --- | --- | --- | --- | --- | --- | --- | --- | --- | --- | --- | --- | --- | --- | --- | --- | --- | --- | --- | --- | --- | --- | --- | --- | --- | --- | --- | --- | --- | --- | --- | --- | --- | --- | --- | --- | --- | --- | --- | --- | --- | --- | --- | --- | --- | --- | --- | --- | --- | --- | --- | --- | --- | --- | --- | --- | --- | --- | --- | --- | --- | --- | --- | --- | --- | --- | --- | --- | --- | --- | --- | --- | --- | --- | --- | --- | --- | --- | --- | --- | --- | --- | --- | --- | --- | --- | --- | --- | --- | --- | --- | --- | --- | --- | --- | --- | --- | --- | --- | --- | --- | --- | --- | --- | --- | --- | --- | --- | --- | --- | --- | --- | --- | --- | --- | --- | --- | --- | --- | --- | --- | --- | --- | --- | --- | --- | --- | --- | --- | --- | --- | --- | --- | --- | --- | --- | --- | --- | --- | --- | --- | --- | --- | --- | --- | --- | --- | --- | --- | --- | --- | --- | --- | --- | --- | --- | --- | --- | --- | --- | --- | --- | --- | --- | --- | --- | --- | --- | --- | --- | --- | --- | --- | --- | --- | --- | --- | --- | --- | --- | --- | --- | --- | --- | --- | --- | --- | --- | --- | --- | --- | --- | --- | --- | --- | --- | --- | --- | --- | --- | --- | --- | --- | --- | --- | --- | --- | --- | --- | --- | --- | --- | --- | --- | --- | --- | --- | --- | --- | --- | --- | --- | --- | --- | --- | --- | --- | --- | --- | --- | --- | --- | --- | --- | --- | --- | --- | --- | --- | --- | --- | --- | --- | --- | --- | --- | --- | --- | --- | --- | --- | --- | --- | --- | --- | --- | --- | --- | --- | --- | --- | --- | --- | --- | --- | --- | --- | --- | --- | --- | --- | --- | --- | --- | --- | --- | --- | --- | --- | --- | --- | --- | --- | --- | --- | --- | --- | --- | --- | --- | --- | --- | --- | --- | --- | --- | --- | --- | --- | --- | --- | --- | --- | --- | --- | --- | --- | --- | --- | --- | --- | --- | --- | --- | --- | --- | --- | --- | --- | --- | --- | --- | --- | --- | --- | --- | --- | --- | --- | --- | --- | --- | --- | --- | --- | --- | --- |

**Table S3.** VA content (mol% and wt%) calculated from the accurate mass measurements and associated compositions in E and VA units from the MALDI mass spectrum of EVA25 (fraction #2).

| \| **Elemental composition** \| \| ***m/z*** \| **ab.**  **(%)** \| **VA** \| **E** \| **VA*ab.** \| **E*ab.** \| \| --- \| --- \| --- \| --- \| --- \| --- \| --- \| --- \| \| C_56_H_110_O_4_Na^+^ \| 869.8279 \| \| 27 \| 2 \| 24 \| 53 \| 641 \| \| C_54_H_104_O_6_Na^+^ \| 871.7720 \| \| 38 \| 3 \| 21 \| 114 \| 795 \| \| C_52_H_98_O_8_Na^+^ \| 873.7149 \| \| 15 \| 4 \| 18 \| 58 \| 262 \| \| C_58_H_114_O_4_Na^+^ \| 897.8606 \| \| 33 \| 2 \| 25 \| 66 \| 830 \| \| C_56_H_108_O_6_Na^+^ \| 899.8012 \| \| 52 \| 3 \| 22 \| 157 \| 1148 \| \| C_54_H_102_O_8_Na^+^ \| 901.7474 \| \| 24 \| 4 \| 19 \| 97 \| 459 \| \| C_60_H_118_O_4_Na^+^ \| 925.8927 \| \| 41 \| 2 \| 26 \| 82 \| 1063 \| \| C_58_H_112_O_6_Na^+^ \| 927.8354 \| \| 66 \| 3 \| 23 \| 199 \| 1527 \| \| C_56_H_106_O_8_Na^+^ \| 929.7765 \| \| 35 \| 4 \| 20 \| 140 \| 701 \| \| C_54_H_100_O_10_Na^+^ \| 931.7196 \| \| 8 \| 5 \| 17 \| 38 \| 128 \| \| C_62_H_122_O_4_Na^+^ \| 953.9217 \| \| 36 \| 2 \| 27 \| 73 \| 980 \| \| C_60_H_116_O_6_Na^+^ \| 955.8650 \| \| 78 \| 3 \| 24 \| 235 \| 1877 \| \| C_58_H_110_O_8_Na^+^ \| 957.8067 \| \| 46 \| 4 \| 21 \| 183 \| 960 \| \| C_56_H_104_O_10_Na^+^ \| 959.7504 \| \| 11 \| 5 \| 18 \| 57 \| 206 \| \| C_64_H_126_O_4_Na^+^ \| 981.9536 \| \| 46 \| 2 \| 28 \| 91 \| 1276 \| \| C_62_H_120_O_6_Na^+^ \| 983.8999 \| \| 87 \| 3 \| 25 \| 260 \| 2170 \| \| C_60_H_114_O_8_Na^+^ \| 985.8410 \| \| 55 \| 4 \| 22 \| 219 \| 1205 \| \| C_58_H_108_O_10_Na^+^ \| 987.7839 \| \| 17 \| 5 \| 19 \| 86 \| 328 \| \| C_66_H_130_O_4_Na^+^ \| 1009.9887 \| \| 44 \| 2 \| 29 \| 87 \| 1266 \| \| C_64_H_124_O_6_Na^+^ \| 1011.9296 \| \| 97 \| 3 \| 26 \| 292 \| 2529 \| \| C_62_H_118_O_8_Na^+^ \| 1013.8724 \| \| 66 \| 4 \| 23 \| 266 \| 1528 \| \| C_60_H_112_O_10_Na^+^ \| 1015.8134 \| \| 20 \| 5 \| 20 \| 102 \| 408 \| \| C_68_H_134_O_4_Na^+^ \| 1038.0176 \| \| 42 \| 2 \| 30 \| 85 \| 1268 \| \| C_66_H_128_O_6_Na^+^ \| 1039.9593 \| \| 97 \| 3 \| 27 \| 291 \| 2622 \| \| C_64_H_122_O_8_Na^+^ \| 1041.9028 \| \| 85 \| 4 \| 24 \| 339 \| 2031 \| \| C_62_H_116_O_10_Na^+^ \| 1043.8443 \| \| 30 \| 5 \| 21 \| 149 \| 624 \| \| C_70_H_138_O_4_Na^+^ \| 1066.0466 \| \| 40 \| 2 \| 31 \| 81 \| 1254 \| \| C_68_H_132_O_6_Na^+^ \| 1067.9917 \| \| 100 \| 3 \| 28 \| 300 \| 2800 \| \| C_66_H_126_O_8_Na^+^ \| 1069.9348 \| \| 81 \| 4 \| 25 \| 325 \| 2031 \| \| C_64_H_120_O_10_Na^+^ \| 1071.8759 \| \| 32 \| 5 \| 22 \| 160 \| 705 \| \| C_62_H_114_O_12_Na^+^ \| 1073.8263 \| \| 7 \| 6 \| 19 \| 41 \| 131 \| \| C_72_H_142_O_4_Na^+^ \| 1094.0793 \| \| 36 \| 2 \| 32 \| 72 \| 1152 \| \| C_70_H_136_O_6_Na^+^ \| 1096.0231 \| \| 89 \| 3 \| 29 \| 268 \| 2595 \| \| C_68_H_130_O_8_Na^+^ \| 1097.9649 \| \| 88 \| 4 \| 26 \| 352 \| 2286 \| \| C_66_H_124_O_10_Na^+^ \| 1099.9045 \| \| 34 \| 5 \| 23 \| 172 \| 791 \| \| C_64_H_118_O_12_Na^+^ \| 1101.8534 \| \| 9 \| 6 \| 20 \| 55 \| 183 \| \| C_74_H_146_O_4_Na^+^ \| 1122.1132 \| \| 28 \| 2 \| 33 \| 55 \| 914 \| \| C_72_H_140_O_6_Na^+^ \| 1124.0547 \| \| 97 \| 3 \| 30 \| 290 \| 2896 \| \| C_70_H_134_O_8_Na^+^ \| 1125.9979 \| \| 90 \| 4 \| 27 \| 361 \| 2439 \| \| C_68_H_128_O_10_Na^+^ \| 1127.9389 \| \| 45 \| 5 \| 24 \| 225 \| 1081 \| \| C_66_H_122_O_12_Na^+^ \| 1129.8816 \| \| 11 \| 6 \| 21 \| 67 \| 236 \| \| C_76_H_150_O_4_Na^+^ \| 1150.1423 \| \| 27 \| 2 \| 34 \| 54 \| 913 \| \| C_74_H_144_O_6_Na^+^ \| 1152.0845 \| \| 87 \| 3 \| 31 \| 261 \| 2695 \| \| C_72_H_138_O_8_Na^+^ \| 1154.0283 \| \| 97 \| 4 \| 28 \| 388 \| 2717 \| \| C_70_H_132_O_10_Na^+^ \| 1155.9698 \| \| 43 \| 5 \| 25 \| 217 \| 1085 \| | \| **Elemental composition** \| \| ***m/z*** \| **ab.**  **(%)** \| **VA** \| **E** \| **VA*ab.** \| **E*ab.** \| \| --- \| --- \| --- \| --- \| --- \| --- \| --- \| --- \| \| C_68_H_126_O_12_Na^+^ \| 1157.9090 \| \| 14 \| 6 \| 22 \| 82 \| 300 \| \| C_66_H_120_O_14_Na^+^ \| 1159.8577 \| \| 4 \| 7 \| 19 \| 28 \| 75 \| \| C_78_H_154_O_4_Na^+^ \| 1178.1732 \| \| 24 \| 2 \| 35 \| 49 \| 853 \| \| C_76_H_148_O_6_Na^+^ \| 1180.1152 \| \| 74 \| 3 \| 32 \| 223 \| 2377 \| \| C_74_H_142_O_8_Na^+^ \| 1182.0587 \| \| 94 \| 4 \| 29 \| 378 \| 2740 \| \| C_72_H_136_O_10_Na^+^ \| 1183.9999 \| \| 50 \| 5 \| 26 \| 249 \| 1297 \| \| C_70_H_130_O_12_Na^+^ \| 1185.9426 \| \| 17 \| 6 \| 23 \| 102 \| 391 \| \| C_68_H_124_O_14_Na^+^ \| 1187.8909 \| \| 4 \| 7 \| 20 \| 31 \| 88 \| \| C_76_H_158_O_4_Na^+^ \| 1206.2056 \| \| 18 \| 2 \| 36 \| 35 \| 639 \| \| C_78_H_152_O_6_Na^+^ \| 1208.1465 \| \| 67 \| 3 \| 33 \| 202 \| 2221 \| \| C_76_H_146_O_8_Na^+^ \| 1210.0889 \| \| 96 \| 4 \| 30 \| 386 \| 2892 \| \| C_74_H_140_O_10_Na^+^ \| 1212.0369 \| \| 54 \| 5 \| 27 \| 272 \| 1467 \| \| C_72_H_134_O_12_Na^+^ \| 1213.9744 \| \| 18 \| 6 \| 24 \| 109 \| 434 \| \| C_70_H_128_O_14_Na^+^ \| 1215.9215 \| \| 4 \| 7 \| 21 \| 28 \| 84 \| \| C_82_H_162_O_4_Na^+^ \| 1234.2358 \| \| 15 \| 2 \| 37 \| 30 \| 556 \| \| C_80_H_156_O_6_Na^+^ \| 1236.1788 \| \| 61 \| 3 \| 34 \| 184 \| 2088 \| \| C_80_H_156_O_6_Na^+^ \| 1238.1193 \| \| 85 \| 4 \| 31 \| 341 \| 2642 \| \| C_80_H_156_O_6_Na^+^ \| 1240.0654 \| \| 56 \| 5 \| 28 \| 281 \| 1571 \| \| C_80_H_156_O_6_Na^+^ \| 1242.0049 \| \| 20 \| 6 \| 25 \| 123 \| 511 \| \| C_80_H_156_O_6_Na^+^ \| 1243.9418 \| \| 5 \| 7 \| 22 \| 33 \| 103 \| \| C_84_H_166_O_4_Na^+^ \| 1262.2687 \| \| 15 \| 2 \| 38 \| 29 \| 551 \| \| C_82_H_160_O_6_Na^+^ \| 1264.2091 \| \| 55 \| 3 \| 35 \| 166 \| 1932 \| \| C_80_H_154_O_8_Na^+^ \| 1266.1550 \| \| 72 \| 4 \| 32 \| 290 \| 2319 \| \| C_78_H_148_O_10_Na^+^ \| 1268.0943 \| \| 58 \| 5 \| 29 \| 290 \| 1684 \| \| C_76_H_142_O_12_Na^+^ \| 1270.0392 \| \| 22 \| 6 \| 26 \| 133 \| 578 \| \| C_74_H_136_O_14_Na^+^ \| 1271.9773 \| \| 5 \| 7 \| 23 \| 34 \| 111 \| \| C_86_H_170_O_4_Na^+^ \| 1290.2936 \| \| 10 \| 2 \| 39 \| 21 \| 402 \| \| C_84_H_164_O_6_Na^+^ \| 1292.2430 \| \| 52 \| 3 \| 36 \| 156 \| 1877 \| \| C_82_H_158_O_8_Na^+^ \| 1294.1814 \| \| 73 \| 4 \| 33 \| 293 \| 2421 \| \| C_80_H_152_O_10_Na^+^ \| 1296.1255 \| \| 54 \| 5 \| 30 \| 272 \| 1631 \| \| C_78_H_146_O_12_Na^+^ \| 1298.0710 \| \| 23 \| 6 \| 27 \| 138 \| 619 \| \| C_76_H_140_O_14_Na^+^ \| 1300.0179 \| \| 7 \| 7 \| 24 \| 49 \| 167 \| \| C_88_H_174_O_4_Na^+^ \| 1318.3252 \| \| 9 \| 2 \| 40 \| 19 \| 378 \| \| C_86_H_168_O_6_Na^+^ \| 1320.2705 \| \| 38 \| 3 \| 37 \| 113 \| 1395 \| \| C_84_H_162_O_8_Na^+^ \| 1322.2131 \| \| 65 \| 4 \| 34 \| 259 \| 2199 \| \| C_82_H_156_O_10_Na^+^ \| 1324.1571 \| \| 52 \| 5 \| 31 \| 258 \| 1600 \| \| C_80_H_150_O_12_Na^+^ \| 1326.1025 \| \| 21 \| 6 \| 28 \| 126 \| 586 \| \| C_78_H_144_O_14_Na^+^ \| 1328.0452 \| \| 8 \| 7 \| 25 \| 59 \| 210 \| \| C_90_H_178_O_4_Na^+^ \| 1346.3537 \| \| 6 \| 2 \| 41 \| 12 \| 248 \| \| C_88_H_172_O_6_Na^+^ \| 1348.3069 \| \| 30 \| 3 \| 38 \| 89 \| 1122 \| \| C_86_H_166_O_8_Na^+^ \| 1350.2404 \| \| 51 \| 4 \| 35 \| 204 \| 1788 \| \| C_84_H_160_O_10_Na^+^ \| 1352.1879 \| \| 51 \| 5 \| 32 \| 253 \| 1622 \| \| C_82_H_154_O_12_Na^+^ \| 1354.1326 \| \| 25 \| 6 \| 29 \| 153 \| 737 \| \| C_80_H_148_O_14_Na^+^ \| 1356.0745 \| \| 9 \| 7 \| 26 \| 64 \| 238 \| \| C_92_H_182_O_4_Na^+^ \| 1374.3867 \| \| 7 \| 2 \| 42 \| 14 \| 284 \| |
| --- | --- | --- | --- | --- | --- | --- | --- | --- | --- | --- | --- | --- | --- | --- | --- | --- | --- | --- | --- | --- | --- | --- | --- | --- | --- | --- | --- | --- | --- | --- | --- | --- | --- | --- | --- | --- | --- | --- | --- | --- | --- | --- | --- | --- | --- | --- | --- | --- | --- | --- | --- | --- | --- | --- | --- | --- | --- | --- | --- | --- | --- | --- | --- | --- | --- | --- | --- | --- | --- | --- | --- | --- | --- | --- | --- | --- | --- | --- | --- | --- | --- | --- | --- | --- | --- | --- | --- | --- | --- | --- | --- | --- | --- | --- | --- | --- | --- | --- | --- | --- | --- | --- | --- | --- | --- | --- | --- | --- | --- | --- | --- | --- | --- | --- | --- | --- | --- | --- | --- | --- | --- | --- | --- | --- | --- | --- | --- | --- | --- | --- | --- | --- | --- | --- | --- | --- | --- | --- | --- | --- | --- | --- | --- | --- | --- | --- | --- | --- | --- | --- | --- | --- | --- | --- | --- | --- | --- | --- | --- | --- | --- | --- | --- | --- | --- | --- | --- | --- | --- | --- | --- | --- | --- | --- | --- | --- | --- | --- | --- | --- | --- | --- | --- | --- | --- | --- | --- | --- | --- | --- | --- | --- | --- | --- | --- | --- | --- | --- | --- | --- | --- | --- | --- | --- | --- | --- | --- | --- | --- | --- | --- | --- | --- | --- | --- | --- | --- | --- | --- | --- | --- | --- | --- | --- | --- | --- | --- | --- | --- | --- | --- | --- | --- | --- | --- | --- | --- | --- | --- | --- | --- | --- | --- | --- | --- | --- | --- | --- | --- | --- | --- | --- | --- | --- | --- | --- | --- | --- | --- | --- | --- | --- | --- | --- | --- | --- | --- | --- | --- | --- | --- | --- | --- | --- | --- | --- | --- | --- | --- | --- | --- | --- | --- | --- | --- | --- | --- | --- | --- | --- | --- | --- | --- | --- | --- | --- | --- | --- | --- | --- | --- | --- | --- | --- | --- | --- | --- | --- | --- | --- | --- | --- | --- | --- | --- | --- | --- | --- | --- | --- | --- | --- | --- | --- | --- | --- | --- | --- | --- | --- | --- | --- | --- | --- | --- | --- | --- | --- | --- | --- | --- | --- | --- | --- | --- | --- | --- | --- | --- | --- | --- | --- | --- | --- | --- | --- | --- | --- | --- | --- | --- | --- | --- | --- | --- | --- | --- | --- | --- | --- | --- | --- | --- | --- | --- | --- | --- | --- | --- | --- | --- | --- | --- | --- | --- | --- | --- | --- | --- | --- | --- | --- | --- | --- | --- | --- | --- | --- | --- | --- | --- | --- | --- | --- | --- | --- | --- | --- | --- | --- | --- | --- | --- | --- | --- | --- | --- | --- | --- | --- | --- | --- | --- | --- | --- | --- | --- | --- | --- | --- | --- | --- | --- | --- | --- | --- | --- | --- | --- | --- | --- | --- | --- | --- | --- | --- | --- | --- | --- | --- | --- | --- | --- | --- | --- | --- | --- | --- | --- | --- | --- | --- | --- | --- | --- | --- | --- | --- | --- | --- | --- | --- | --- | --- | --- | --- | --- | --- | --- | --- | --- | --- | --- | --- | --- | --- | --- | --- | --- | --- | --- | --- | --- | --- | --- | --- | --- | --- | --- | --- | --- | --- | --- | --- | --- | --- | --- | --- | --- | --- | --- | --- | --- | --- | --- | --- | --- | --- | --- | --- | --- | --- | --- | --- | --- | --- | --- | --- | --- | --- | --- | --- | --- | --- | --- | --- | --- | --- | --- | --- | --- | --- | --- | --- | --- | --- | --- | --- | --- | --- | --- | --- | --- | --- | --- | --- | --- | --- | --- | --- | --- | --- | --- | --- | --- | --- | --- | --- | --- | --- | --- | --- | --- | --- | --- | --- | --- | --- | --- | --- | --- | --- | --- | --- | --- | --- | --- | --- | --- | --- | --- | --- | --- | --- | --- | --- | --- | --- | --- | --- | --- | --- | --- | --- | --- | --- | --- | --- | --- | --- | --- | --- | --- | --- | --- | --- | --- | --- | --- | --- | --- | --- | --- | --- | --- | --- | --- | --- | --- | --- | --- | --- | --- | --- | --- | --- | --- | --- | --- | --- | --- | --- | --- | --- | --- | --- | --- | --- | --- | --- | --- | --- | --- | --- | --- | --- | --- | --- | --- | --- | --- | --- | --- | --- | --- | --- | --- | --- | --- | --- | --- | --- | --- | --- | --- | --- | --- | --- | --- | --- | --- | --- | --- | --- | --- | --- | --- | --- | --- | --- | --- | --- | --- | --- | --- | --- | --- | --- | --- | --- | --- | --- | --- | --- | --- | --- | --- | --- | --- | --- | --- | --- | --- | --- | --- | --- | --- | --- | --- | --- | --- | --- | --- | --- | --- | --- | --- | --- | --- | --- | --- | --- | --- | --- | --- | --- | --- |

**Table S3.** (next)

| \| **Elemental composition** \| ***m/z*** \| **ab.**  **(%)** \| **VA** \| **E** \| **VA*ab.** \| **E*ab.** \| \| --- \| --- \| --- \| --- \| --- \| --- \| --- \| \| C_90_H_176_O_6_Na^+^ \| 1376.3345 \| 25 \| 3 \| 39 \| 75 \| 979 \| \| C_88_H_170_O_8_Na^+^ \| 1378.2793 \| 49 \| 4 \| 36 \| 197 \| 1770 \| \| C_86_H_170_O_10_Na^+^ \| 1380.2213 \| 50 \| 5 \| 33 \| 252 \| 1663 \| \| C_84_H_164_O_12_Na^+^ \| 1382.1561 \| 25 \| 6 \| 30 \| 150 \| 751 \| \| C_82_H_158_O_14_Na^+^ \| 1384.1051 \| 9 \| 7 \| 27 \| 65 \| 252 \| \| C_80_H_152_O_16_Na^+^ \| 1386.0512 \| 3 \| 8 \| 24 \| 21 \| 64 \| \| C_94_H_186_O_4_Na^+^ \| 1402.4238 \| 5 \| 2 \| 43 \| 11 \| 236 \| \| C_92_H_180_O_6_Na^+^ \| 1404.3611 \| 21 \| 3 \| 40 \| 64 \| 855 \| \| C_90_H_174_O_8_Na^+^ \| 1406.3084 \| 43 \| 4 \| 37 \| 171 \| 1582 \| \| C_88_H_168_O_10_Na^+^ \| 1408.2484 \| 46 \| 5 \| 34 \| 228 \| 1549 \| \| C_86_H_162_O_12_Na^+^ \| 1410.1941 \| 22 \| 6 \| 31 \| 134 \| 694 \| \| C_84_H_156_O_14_Na^+^ \| 1412.1281 \| 6 \| 7 \| 28 \| 41 \| 165 \| \| C_82_H_150_O_16_Na^+^ \| 1414.0765 \| 3 \| -9 \| 77 \| -31 \| 265 \| \| C_96_H_190_O_4_Na^+^ \| 1430.4608 \| 3 \| 2 \| 44 \| 6 \| 134 \| \| C_94_H_184_O_6_Na^+^ \| 1432.3956 \| 17 \| 3 \| 41 \| 50 \| 679 \| \| C_92_H_178_O_8_Na^+^ \| 1434.3361 \| 35 \| 4 \| 38 \| 141 \| 1340 \| \| C_90_H_172_O_10_Na^+^ \| 1436.2779 \| 36 \| 5 \| 35 \| 182 \| 1273 \| \| C_88_H_166_O_12_Na^+^ \| 1438.2297 \| 22 \| 6 \| 32 \| 131 \| 700 \| \| C_86_H_160_O_14_Na^+^ \| 1440.1654 \| 10 \| 7 \| 29 \| 71 \| 296 \| \| C_84_H_154_O_16_Na^+^ \| 1442.1155 \| 4 \| 8 \| 26 \| 29 \| 94 \| \| C_96_H_188_O_6_Na^+^ \| 1460.4270 \| 12 \| 3 \| 42 \| 36 \| 497 \| \| C_94_H_182_O_8_Na^+^ \| 1462.3731 \| 26 \| 4 \| 39 \| 105 \| 1024 \| \| C_92_H_176_O_10_Na^+^ \| 1464.3120 \| 32 \| 5 \| 36 \| 161 \| 1156 \| \| C_90_H_170_O_12_Na^+^ \| 1466.2569 \| 20 \| 6 \| 33 \| 123 \| 676 \| \| C_88_H_164_O_14_Na^+^ \| 1468.2027 \| 8 \| 7 \| 30 \| 54 \| 233 \| \| C_86_H_158_O_16_Na^+^ \| 1470.1476 \| 3 \| 8 \| 27 \| 25 \| 85 \| \| C_98_H_192_O_6_Na^+^ \| 1488.4623 \| 9 \| 3 \| 43 \| 28 \| 402 \| \| C_96_H_186_O_8_Na^+^ \| 1490.4011 \| 23 \| 4 \| 40 \| 94 \| 938 \| \| C_94_H_180_O_10_Na^+^ \| 1492.3489 \| 28 \| 5 \| 37 \| 142 \| 1050 \| \| C_92_H_174_O_12_Na^+^ \| 1494.2835 \| 20 \| 6 \| 34 \| 117 \| 665 \| \| C_90_H_168_O_14_Na^+^ \| 1496.2293 \| 9 \| 7 \| 31 \| 62 \| 272 \| \| C_88_H_162_O_16_Na^+^ \| 1498.1784 \| 3 \| 8 \| 28 \| 22 \| 77 \| \| C_100_H_196_O_6_Na^+^ \| 1516.4896 \| 7 \| 3 \| 44 \| 22 \| 324 \| \| C_98_H_190_O_8_Na^+^ \| 1518.4356 \| 18 \| 4 \| 41 \| 74 \| 756 \| \| C_96_H_184_O_10_Na^+^ \| 1520.3692 \| 22 \| 5 \| 38 \| 109 \| 832 \| \| C_94_H_178_O_12_Na^+^ \| 1522.3161 \| 18 \| 6 \| 35 \| 109 \| 635 \| \|  \|  \|  \|  \|  \|  \|  \| \|  \|  \|  \|  \|  \|  \|  \| | \| **Elemental composition** \| ***m/z*** \| **ab.**  **(%)** \| **VA** \| **E** \| **VA*ab.** \| **E*ab.** \| \| --- \| --- \| --- \| --- \| --- \| --- \| --- \| \| C_92_H_172_O_14_Na^+^ \| 1524.2509 \| 5 \| 7 \| 32 \| 38 \| 176 \| \| C_90_H_166_O_16_Na^+^ \| 1526.1853 \| 1 \| 8 \| 78 \| -12 \| 113 \| \| C_102_H_200_O_6_Na^+^ \| 1544.5180 \| 5 \| 3 \| 45 \| 15 \| 218 \| \| C_100_H_194_O_8_Na^+^ \| 1546.4704 \| 15 \| 4 \| 42 \| 61 \| 640 \| \| C_98_H_188_O_10_Na^+^ \| 1548.4090 \| 19 \| 5 \| 39 \| 94 \| 732 \| \| C_96_H_182_O_12_Na^+^ \| 1550.3514 \| 15 \| 6 \| 36 \| 90 \| 541 \| \| C_94_H_176_O_14_Na^+^ \| 1552.2955 \| 6 \| 7 \| 33 \| 43 \| 202 \| \| C_92_H_170_O_16_Na^+^ \| 1554.2383 \| 4 \| 8 \| 30 \| 28 \| 107 \| \| C_104_H_204_O_6_Na^+^ \| 1572.5498 \| 5 \| 3 \| 46 \| 15 \| 234 \| \| C_102_H_198_O_8_Na^+^ \| 1574.4980 \| 11 \| 4 \| 43 \| 45 \| 489 \| \| C_100_H_192_O_10_Na^+^ \| 1576.4430 \| 14 \| 5 \| 40 \| 68 \| 547 \| \| C_98_H_186_O_12_Na^+^ \| 1578.3810 \| 14 \| 6 \| 37 \| 86 \| 530 \| \| C_96_H_180_O_14_Na^+^ \| 1580.3271 \| 7 \| 7 \| 34 \| 47 \| 228 \| \| C_94_H_174_O_16_Na^+^ \| 1582.2742 \| 3 \| 8 \| 31 \| 25 \| 97 \| \| C_106_H_208_O_6_Na^+^ \| 1600.5772 \| 4 \| 3 \| 47 \| 11 \| 168 \| \| C_104_H_202_O_8_Na^+^ \| 1602.5316 \| 10 \| 4 \| 44 \| 40 \| 444 \| \| C_102_H_196_O_10_Na^+^ \| 1604.4590 \| 11 \| 5 \| 41 \| 53 \| 438 \| \| C_100_H_190_O_12_Na^+^ \| 1606.4099 \| 11 \| 6 \| 38 \| 66 \| 416 \| \| C_98_H_184_O_14_Na^+^ \| 1608.3520 \| 8 \| 7 \| 35 \| 55 \| 276 \| \| C_96_H_178_O_16_Na^+^ \| 1610.3086 \| 3 \| 8 \| 32 \| 27 \| 107 \| \|  \|  \|  \|  \|  \| **VAav** \| **Eav** \| \|  \|  \|  \|  \|  \| 3.9 \| 29.6 \| \|  \|  \|  \| %VA (mol) \| 0.12 \|  \|  \| \|  \|  \|  \| **%VA (wt)** \| **0.29** \|  \|  \| \|  \|  \|  \|  \|  \|  \|  \| \|  \|  \|  \|  \|  \|  \|  \| \|  \|  \|  \|  \|  \|  \|  \| \|  \|  \|  \|  \|  \|  \|  \| \|  \|  \|  \|  \|  \|  \|  \| \|  \|  \|  \|  \|  \|  \|  \| \|  \|  \|  \|  \|  \|  \|  \| \|  \|  \|  \|  \|  \|  \|  \| \|  \|  \|  \|  \|  \|  \|  \| \|  \|  \|  \|  \|  \|  \|  \| \|  \|  \|  \|  \| \|  \|  \| \|  \|  \|  \|  \| \|  \|  \| \|  \|  \|  \|  \|  \|  \|  \| \|  \|  \|  \|  \|  \|  \|  \| |
| --- | --- | --- | --- | --- | --- | --- | --- | --- | --- | --- | --- | --- | --- | --- | --- | --- | --- | --- | --- | --- | --- | --- | --- | --- | --- | --- | --- | --- | --- | --- | --- | --- | --- | --- | --- | --- | --- | --- | --- | --- | --- | --- | --- | --- | --- | --- | --- | --- | --- | --- | --- | --- | --- | --- | --- | --- | --- | --- | --- | --- | --- | --- | --- | --- | --- | --- | --- | --- | --- | --- | --- | --- | --- | --- | --- | --- | --- | --- | --- | --- | --- | --- | --- | --- | --- | --- | --- | --- | --- | --- | --- | --- | --- | --- | --- | --- | --- | --- | --- | --- | --- | --- | --- | --- | --- | --- | --- | --- | --- | --- | --- | --- | --- | --- | --- | --- | --- | --- | --- | --- | --- | --- | --- | --- | --- | --- | --- | --- | --- | --- | --- | --- | --- | --- | --- | --- | --- | --- | --- | --- | --- | --- | --- | --- | --- | --- | --- | --- | --- | --- | --- | --- | --- | --- | --- | --- | --- | --- | --- | --- | --- | --- | --- | --- | --- | --- | --- | --- | --- | --- | --- | --- | --- | --- | --- | --- | --- | --- | --- | --- | --- | --- | --- | --- | --- | --- | --- | --- | --- | --- | --- | --- | --- | --- | --- | --- | --- | --- | --- | --- | --- | --- | --- | --- | --- | --- | --- | --- | --- | --- | --- | --- | --- | --- | --- | --- | --- | --- | --- | --- | --- | --- | --- | --- | --- | --- | --- | --- | --- | --- | --- | --- | --- | --- | --- | --- | --- | --- | --- | --- | --- | --- | --- | --- | --- | --- | --- | --- | --- | --- | --- | --- | --- | --- | --- | --- | --- | --- | --- | --- | --- | --- | --- | --- | --- | --- | --- | --- | --- | --- | --- | --- | --- | --- | --- | --- | --- | --- | --- | --- | --- | --- | --- | --- | --- | --- | --- | --- | --- | --- | --- | --- | --- | --- | --- | --- | --- | --- | --- | --- | --- | --- | --- | --- | --- | --- | --- | --- | --- | --- | --- | --- | --- | --- | --- | --- | --- | --- | --- | --- | --- | --- | --- | --- | --- | --- | --- | --- | --- | --- | --- | --- | --- | --- | --- | --- | --- | --- | --- | --- | --- | --- | --- | --- | --- | --- | --- | --- | --- | --- | --- | --- | --- | --- | --- | --- | --- | --- | --- | --- | --- | --- | --- | --- | --- | --- | --- | --- | --- | --- | --- | --- | --- | --- | --- | --- | --- | --- | --- | --- | --- | --- | --- | --- | --- | --- | --- | --- | --- | --- | --- | --- | --- | --- | --- | --- | --- | --- | --- | --- | --- | --- | --- | --- | --- | --- | --- | --- | --- | --- | --- | --- | --- | --- | --- | --- | --- | --- | --- | --- | --- | --- | --- | --- | --- | --- | --- | --- | --- | --- | --- | --- | --- | --- | --- | --- | --- | --- | --- | --- | --- | --- | --- | --- | --- | --- | --- | --- | --- | --- | --- | --- | --- | --- | --- | --- | --- | --- | --- | --- | --- | --- | --- | --- | --- | --- | --- | --- | --- | --- | --- | --- | --- | --- | --- | --- | --- | --- | --- | --- | --- | --- | --- | --- | --- | --- | --- | --- | --- | --- | --- | --- | --- | --- | --- | --- | --- | --- | --- | --- | --- | --- | --- | --- | --- | --- | --- | --- | --- | --- | --- | --- | --- | --- | --- | --- | --- | --- | --- | --- | --- | --- | --- | --- | --- | --- | --- | --- | --- | --- | --- | --- | --- | --- | --- | --- | --- | --- | --- | --- | --- | --- | --- | --- | --- | --- | --- |

**Table S4.** VA content (mol% and wt%) calculated from the accurate mass measurements and associated compositions in E and VA units from the MALDI mass spectrum of EVA18 (fraction #2).

| \| **Elemental composition** \| ***m/z*** \| **ab.**  **(%)** \| **VA** \| **E** \| **VA*ab.** \| **E*ab.** \| \| --- \| --- \| --- \| --- \| --- \| --- \| --- \| \| C_58_H_116_O_2_Na^+^ \| 867.8709 \| 12 \| 1 \| 27 \| 12 \| 314 \| \| C_56_H_110_O_4_Na^+^ \| 869.8197 \| 34 \| 2 \| 24 \| 69 \| 824 \| \| C_54_H_104_O_6_Na^+^ \| 871.7605 \| 30 \| 3 \| 21 \| 90 \| 628 \| \| C_58_H_114_O_4_Na^+^ \| 897.8463 \| 45 \| 2 \| 25 \| 91 \| 1133 \| \| C_56_H_108_O_6_Na^+^ \| 899.7939 \| 37 \| 3 \| 22 \| 112 \| 821 \| \| C_54_H_102_O_8_Na^+^ \| 901.7401 \| 17 \| 4 \| 19 \| 68 \| 325 \| \| C_62_H_124_O_2_Na^+^ \| 923.9385 \| 16 \| 1 \| 29 \| 16 \| 470 \| \| C_60_H_118_O_4_Na^+^ \| 925.8826 \| 56 \| 2 \| 26 \| 112 \| 1454 \| \| C_58_H_112_O_6_Na^+^ \| 927.8252 \| 45 \| 3 \| 23 \| 135 \| 1035 \| \| C_56_H_106_O_8_Na^+^ \| 929.7664 \| 17 \| 4 \| 20 \| 67 \| 336 \| \| C_64_H_128_O_2_Na^+^ \| 951.9707 \| 11 \| 1 \| 30 \| 11 \| 337 \| \| C_62_H_122_O_4_Na^+^ \| 953.9156 \| 59 \| 2 \| 27 \| 117 \| 1583 \| \| C_60_H_116_O_6_Na^+^ \| 955.8589 \| 66 \| 3 \| 24 \| 199 \| 1589 \| \| C_58_H_110_O_8_Na^+^ \| 957.8042 \| 21 \| 4 \| 21 \| 84 \| 442 \| \| C_66_H_132_O_2_Na^+^ \| 980.0035 \| 15 \| 1 \| 31 \| 15 \| 470 \| \| C_64_H_126_O_4_Na^+^ \| 981.9479 \| 65 \| 2 \| 28 \| 130 \| 1815 \| \| C_62_H_120_O_6_Na^+^ \| 983.8907 \| 62 \| 3 \| 25 \| 185 \| 1542 \| \| C_60_H_114_O_8_Na^+^ \| 985.8353 \| 35 \| 4 \| 22 \| 141 \| 776 \| \| C_68_H_136_O_2_Na^+^ \| 1008.0370 \| 12 \| 1 \| 32 \| 12 \| 371 \| \| C_66_H_130_O_4_Na^+^ \| 1009.9834 \| 70 \| 2 \| 29 \| 140 \| 2023 \| \| C_64_H_124_O_6_Na^+^ \| 1011.9243 \| 86 \| 3 \| 26 \| 257 \| 2228 \| \| C_62_H_118_O_8_Na^+^ \| 1013.8671 \| 31 \| 4 \| 23 \| 123 \| 705 \| \| C_70_H_140_O_2_Na^+^ \| 1036.0726 \| 12 \| 1 \| 33 \| 12 \| 390 \| \| C_68_H_134_O_4_Na^+^ \| 1038.0125 \| 61 \| 2 \| 30 \| 122 \| 1826 \| \| C_66_H_128_O_6_Na^+^ \| 1039.9578 \| 89 \| 3 \| 27 \| 268 \| 2414 \| \| C_64_H_122_O_8_Na^+^ \| 1041.9013 \| 45 \| 4 \| 24 \| 181 \| 1089 \| \| C_70_H_138_O_4_Na^+^ \| 1066.0490 \| 61 \| 2 \| 31 \| 122 \| 1893 \| \| C_68_H_132_O_6_Na^+^ \| 1067.9866 \| 79 \| 3 \| 28 \| 236 \| 2199 \| \| C_66_H_126_O_8_Na^+^ \| 1069.9297 \| 52 \| 4 \| 25 \| 209 \| 1306 \| \| C_64_H_120_O_10_Na^+^ \| 1071.8745 \| 16 \| 5 \| 22 \| 81 \| 358 \| \| C_74_H_148_O_2_Na^+^ \| 1092.1397 \| 11 \| 1 \| 35 \| 11 \| 391 \| \| C_72_H_142_O_4_Na^+^ \| 1094.0741 \| 55 \| 2 \| 32 \| 109 \| 1751 \| \| C_70_H_136_O_6_Na^+^ \| 1096.0256 \| 98 \| 3 \| 29 \| 295 \| 2848 \| \| C_68_H_130_O_8_Na^+^ \| 1097.9673 \| 47 \| 4 \| 26 \| 187 \| 1217 \| \| C_66_H_124_O_10_Na^+^ \| 1099.9069 \| 15 \| 5 \| 23 \| 76 \| 352 \| \| C_74_H_146_O_4_Na^+^ \| 1122.1117 \| 60 \| 2 \| 33 \| 120 \| 1975 \| \| C_72_H_140_O_6_Na^+^ \| 1124.0532 \| 100 \| 3 \| 30 \| 300 \| 3000 \| \| C_70_H_134_O_8_Na^+^ \| 1125.9964 \| 65 \| 4 \| 27 \| 259 \| 1750 \| \| C_68_H_128_O_10_Na^+^ \| 1127.9451 \| 15 \| 5 \| 24 \| 75 \| 360 \| \| C_76_H_150_O_4_Na^+^ \| 1150.1445 \| 46 \| 2 \| 34 \| 92 \| 1565 \| \| C_74_H_144_O_6_Na^+^ \| 1152.0905 \| 93 \| 3 \| 31 \| 278 \| 2868 \| \| C_72_H_138_O_8_Na^+^ \| 1154.0264 \| 64 \| 4 \| 28 \| 256 \| 1789 \| \| C_70_H_132_O_10_Na^+^ \| 1155.9718 \| 21 \| 5 \| 25 \| 107 \| 537 \| \| C_78_H_154_O_4_Na^+^ \| 1178.1750 \| 44 \| 2 \| 35 \| 89 \| 1553 \| \| C_76_H_148_O_6_Na^+^ \| 1180.1209 \| 93 \| 3 \| 32 \| 278 \| 2963 \| | \| **Elemental composition** \| ***m/z*** \| **ab.**  **(%)** \| **VA** \| **E** \| **VA*ab.** \| **E*ab.** \| \| --- \| --- \| --- \| --- \| --- \| --- \| --- \| \| C_74_H_142_O_8_Na^+^ \| 1182.0604 \| 64 \| 4 \| 29 \| 256 \| 1852 \| \| C_72_H_136_O_10_Na^+^ \| 1184.0055 \| 26 \| 5 \| 26 \| 130 \| 679 \| \| C_76_H_158_O_4_Na^+^ \| 1206.1989 \| 31 \| 2 \| 36 \| 61 \| 1105 \| \| C_78_H_152_O_6_Na^+^ \| 1208.1478 \| 95 \| 3 \| 33 \| 286 \| 3146 \| \| C_76_H_146_O_8_Na^+^ \| 1210.0941 \| 65 \| 4 \| 30 \| 260 \| 1951 \| \| C_74_H_140_O_10_Na^+^ \| 1212.0421 \| 24 \| 5 \| 27 \| 118 \| 639 \| \| C_82_H_162_O_4_Na^+^ \| 1234.2365 \| 39 \| 2 \| 37 \| 79 \| 1455 \| \| C_80_H_156_O_6_Na^+^ \| 1236.1835 \| 83 \| 3 \| 34 \| 250 \| 2831 \| \| C_80_H_156_O_6_Na^+^ \| 1238.1240 \| 73 \| 4 \| 31 \| 291 \| 2252 \| \| C_80_H_156_O_6_Na^+^ \| 1240.0659 \| 31 \| 5 \| 28 \| 155 \| 870 \| \| C_80_H_156_O_6_Na^+^ \| 1242.0216 \| 7 \| 6 \| 25 \| 42 \| 175 \| \| C_84_H_166_O_4_Na^+^ \| 1262.2727 \| 33 \| 2 \| 38 \| 67 \| 1269 \| \| C_82_H_160_O_6_Na^+^ \| 1264.2049 \| 69 \| 3 \| 35 \| 208 \| 2430 \| \| C_80_H_154_O_8_Na^+^ \| 1266.1508 \| 61 \| 4 \| 32 \| 243 \| 1942 \| \| C_78_H_148_O_10_Na^+^ \| 1268.0982 \| 27 \| 5 \| 29 \| 133 \| 769 \| \| C_76_H_142_O_12_Na^+^ \| 1270.0430 \| 8 \| 6 \| 26 \| 46 \| 198 \| \| C_86_H_170_O_4_Na^+^ \| 1290.2969 \| 27 \| 2 \| 39 \| 54 \| 1050 \| \| C_84_H_164_O_6_Na^+^ \| 1292.2462 \| 70 \| 3 \| 36 \| 210 \| 2519 \| \| C_82_H_158_O_8_Na^+^ \| 1294.1846 \| 80 \| 4 \| 33 \| 320 \| 2640 \| \| C_80_H_152_O_10_Na^+^ \| 1296.1285 \| 28 \| 5 \| 30 \| 140 \| 843 \| \| C_78_H_146_O_12_Na^+^ \| 1298.0740 \| 10 \| 6 \| 27 \| 57 \| 257 \| \| C_88_H_174_O_4_Na^+^ \| 1318.3233 \| 20 \| 2 \| 40 \| 40 \| 809 \| \| C_86_H_168_O_6_Na^+^ \| 1320.2728 \| 67 \| 3 \| 37 \| 200 \| 2462 \| \| C_84_H_162_O_8_Na^+^ \| 1322.2153 \| 71 \| 4 \| 34 \| 286 \| 2428 \| \| C_82_H_156_O_10_Na^+^ \| 1324.1592 \| 30 \| 5 \| 31 \| 151 \| 936 \| \| C_80_H_150_O_12_Na^+^ \| 1326.1088 \| 11 \| 6 \| 28 \| 67 \| 314 \| \| C_90_H_178_O_4_Na^+^ \| 1346.3676 \| 21 \| 2 \| 41 \| 41 \| 844 \| \| C_88_H_172_O_6_Na^+^ \| 1348.3038 \| 56 \| 3 \| 38 \| 169 \| 2138 \| \| C_86_H_166_O_8_Na^+^ \| 1350.2499 \| 51 \| 4 \| 35 \| 205 \| 1796 \| \| C_84_H_160_O_10_Na^+^ \| 1352.1931 \| 26 \| 5 \| 32 \| 131 \| 836 \| \| C_82_H_154_O_12_Na^+^ \| 1354.1293 \| 9 \| 6 \| 29 \| 55 \| 267 \| \| C_92_H_182_O_4_Na^+^ \| 1374.3910 \| 20 \| 2 \| 42 \| 40 \| 846 \| \| C_90_H_176_O_6_Na^+^ \| 1376.3387 \| 47 \| 3 \| 39 \| 141 \| 1835 \| \| C_88_H_170_O_8_Na^+^ \| 1378.2792 \| 49 \| 4 \| 36 \| 197 \| 1773 \| \| C_86_H_170_O_10_Na^+^ \| 1380.2211 \| 28 \| 5 \| 33 \| 140 \| 924 \| \| C_84_H_164_O_12_Na^+^ \| 1382.1729 \| 10 \| 6 \| 30 \| 58 \| 288 \| \| C_94_H_186_O_4_Na^+^ \| 1402.4225 \| 16 \| 2 \| 43 \| 32 \| 698 \| \| C_92_H_180_O_6_Na^+^ \| 1404.3641 \| 36 \| 3 \| 40 \| 109 \| 1450 \| \| C_90_H_174_O_8_Na^+^ \| 1406.3113 \| 50 \| 4 \| 37 \| 202 \| 1867 \| \| C_88_H_168_O_10_Na^+^ \| 1408.2555 \| 28 \| 5 \| 34 \| 138 \| 941 \| \| C_86_H_162_O_12_Na^+^ \| 1410.2011 \| 11 \| 6 \| 31 \| 69 \| 354 \| \| C_96_H_190_O_4_Na^+^ \| 1430.4407 \| 8 \| 2 \| 44 \| 16 \| 363 \| \| C_94_H_184_O_6_Na^+^ \| 1432.3885 \| 35 \| 3 \| 41 \| 105 \| 1433 \| \| C_92_H_178_O_8_Na^+^ \| 1434.3419 \| 48 \| 4 \| 38 \| 193 \| 1834 \| \| C_90_H_172_O_10_Na^+^ \| 1436.2836 \| 30 \| 5 \| 35 \| 151 \| 1054 \| |
| --- | --- | --- | --- | --- | --- | --- | --- | --- | --- | --- | --- | --- | --- | --- | --- | --- | --- | --- | --- | --- | --- | --- | --- | --- | --- | --- | --- | --- | --- | --- | --- | --- | --- | --- | --- | --- | --- | --- | --- | --- | --- | --- | --- | --- | --- | --- | --- | --- | --- | --- | --- | --- | --- | --- | --- | --- | --- | --- | --- | --- | --- | --- | --- | --- | --- | --- | --- | --- | --- | --- | --- | --- | --- | --- | --- | --- | --- | --- | --- | --- | --- | --- | --- | --- | --- | --- | --- | --- | --- | --- | --- | --- | --- | --- | --- | --- | --- | --- | --- | --- | --- | --- | --- | --- | --- | --- | --- | --- | --- | --- | --- | --- | --- | --- | --- | --- | --- | --- | --- | --- | --- | --- | --- | --- | --- | --- | --- | --- | --- | --- | --- | --- | --- | --- | --- | --- | --- | --- | --- | --- | --- | --- | --- | --- | --- | --- | --- | --- | --- | --- | --- | --- | --- | --- | --- | --- | --- | --- | --- | --- | --- | --- | --- | --- | --- | --- | --- | --- | --- | --- | --- | --- | --- | --- | --- | --- | --- | --- | --- | --- | --- | --- | --- | --- | --- | --- | --- | --- | --- | --- | --- | --- | --- | --- | --- | --- | --- | --- | --- | --- | --- | --- | --- | --- | --- | --- | --- | --- | --- | --- | --- | --- | --- | --- | --- | --- | --- | --- | --- | --- | --- | --- | --- | --- | --- | --- | --- | --- | --- | --- | --- | --- | --- | --- | --- | --- | --- | --- | --- | --- | --- | --- | --- | --- | --- | --- | --- | --- | --- | --- | --- | --- | --- | --- | --- | --- | --- | --- | --- | --- | --- | --- | --- | --- | --- | --- | --- | --- | --- | --- | --- | --- | --- | --- | --- | --- | --- | --- | --- | --- | --- | --- | --- | --- | --- | --- | --- | --- | --- | --- | --- | --- | --- | --- | --- | --- | --- | --- | --- | --- | --- | --- | --- | --- | --- | --- | --- | --- | --- | --- | --- | --- | --- | --- | --- | --- | --- | --- | --- | --- | --- | --- | --- | --- | --- | --- | --- | --- | --- | --- | --- | --- | --- | --- | --- | --- | --- | --- | --- | --- | --- | --- | --- | --- | --- | --- | --- | --- | --- | --- | --- | --- | --- | --- | --- | --- | --- | --- | --- | --- | --- | --- | --- | --- | --- | --- | --- | --- | --- | --- | --- | --- | --- | --- | --- | --- | --- | --- | --- | --- | --- | --- | --- | --- | --- | --- | --- | --- | --- | --- | --- | --- | --- | --- | --- | --- | --- | --- | --- | --- | --- | --- | --- | --- | --- | --- | --- | --- | --- | --- | --- | --- | --- | --- | --- | --- | --- | --- | --- | --- | --- | --- | --- | --- | --- | --- | --- | --- | --- | --- | --- | --- | --- | --- | --- | --- | --- | --- | --- | --- | --- | --- | --- | --- | --- | --- | --- | --- | --- | --- | --- | --- | --- | --- | --- | --- | --- | --- | --- | --- | --- | --- | --- | --- | --- | --- | --- | --- | --- | --- | --- | --- | --- | --- | --- | --- | --- | --- | --- | --- | --- | --- | --- | --- | --- | --- | --- | --- | --- | --- | --- | --- | --- | --- | --- | --- | --- | --- | --- | --- | --- | --- | --- | --- | --- | --- | --- | --- | --- | --- | --- | --- | --- | --- | --- | --- | --- | --- | --- | --- | --- | --- | --- | --- | --- | --- | --- | --- | --- | --- | --- | --- | --- | --- | --- | --- | --- | --- | --- | --- | --- | --- | --- | --- | --- | --- | --- | --- | --- | --- | --- | --- | --- | --- | --- | --- | --- | --- | --- | --- | --- | --- | --- | --- | --- | --- | --- | --- | --- | --- | --- | --- | --- | --- | --- | --- | --- | --- | --- | --- | --- | --- | --- | --- | --- | --- | --- | --- | --- | --- | --- | --- | --- | --- | --- | --- | --- | --- | --- | --- | --- | --- | --- | --- | --- | --- | --- | --- | --- | --- | --- | --- | --- | --- | --- | --- | --- | --- | --- | --- | --- | --- | --- | --- | --- | --- | --- | --- | --- | --- | --- | --- | --- | --- | --- | --- | --- | --- | --- | --- | --- | --- | --- | --- | --- |

**Table S4.** (next)

| \| **Elemental composition** \| ***m/z*** \| **ab.**  **(%)** \| **VA** \| **E** \| **VA*ab.** \| **E*ab.** \| \| --- \| --- \| --- \| --- \| --- \| --- \| --- \| \| C_88_H_166_O_12_Na^+^ \| 1438.2222 \| 9 \| 6 \| 32 \| 57 \| 304 \| \| C_98_H_194_O_4_Na^+^ \| 1458.4676 \| 7 \| 2 \| 45 \| 15 \| 332 \| \| C_96_H_188_O_6_Na^+^ \| 1460.4255 \| 31 \| 3 \| 42 \| 92 \| 1284 \| \| C_94_H_182_O_8_Na^+^ \| 1462.3672 \| 45 \| 4 \| 39 \| 178 \| 1738 \| \| C_92_H_176_O_10_Na^+^ \| 1464.3101 \| 23 \| 5 \| 36 \| 114 \| 822 \| \| C_100_H_198_O_4_Na^+^ \| 1486.5114 \| 8 \| 2 \| 46 \| 16 \| 376 \| \| C_98_H_192_O_6_Na^+^ \| 1488.4545 \| 21 \| 3 \| 43 \| 64 \| 915 \| \| C_96_H_186_O_8_Na^+^ \| 1490.4005 \| 36 \| 4 \| 40 \| 142 \| 1425 \| \| C_94_H_180_O_10_Na^+^ \| 1492.3544 \| 18 \| 5 \| 37 \| 91 \| 671 \| \| C_92_H_174_O_12_Na^+^ \| 1494.2868 \| 13 \| 6 \| 34 \| 79 \| 445 \| \| C_100_H_196_O_6_Na^+^ \| 1516.4897 \| 17 \| 3 \| 44 \| 51 \| 741 \| \| C_98_H_190_O_8_Na^+^ \| 1518.4366 \| 28 \| 4 \| 41 \| 112 \| 1143 \| \| C_96_H_184_O_10_Na^+^ \| 1520.3776 \| 20 \| 5 \| 38 \| 100 \| 759 \| \| C_94_H_178_O_12_Na^+^ \| 1522.3134 \| 10 \| 6 \| 35 \| 58 \| 339 \| \| C_102_H_200_O_6_Na^+^ \| 1544.5250 \| 13 \| 3 \| 45 \| 39 \| 588 \| \| C_100_H_194_O_8_Na^+^ \| 1546.4607 \| 23 \| 4 \| 42 \| 93 \| 979 \| \| C_98_H_188_O_10_Na^+^ \| 1548.3955 \| 14 \| 5 \| 39 \| 72 \| 558 \| \| C_96_H_182_O_12_Na^+^ \| 1550.3471 \| 8 \| 6 \| 36 \| 47 \| 281 \| \| C_104_H_204_O_6_Na^+^ \| 1572.5540 \| 9 \| 3 \| 46 \| 28 \| 431 \| \| C_102_H_198_O_8_Na^+^ \| 1574.4886 \| 16 \| 4 \| 43 \| 62 \| 669 \| \| C_100_H_192_O_10_Na^+^ \| 1576.4231 \| 15 \| 5 \| 40 \| 74 \| 595 \| \| C_98_H_186_O_12_Na^+^ \| 1578.3698 \| 6 \| 6 \| 37 \| 35 \| 217 \| \|  \|  \|  \|  \|  \|  \|  \| \|  \|  \|  \|  \|  \| **VAav** \| **Eav** \| \|  \|  \|  \|  \|  \| 3.3 \| 31.5 \| \|  \|  \|  \| %VA (mol) \| 0.09 \|  \|  \| \|  \|  \|  \| **%VA (wt)** \| **0.24** \|  \|  \| \|  \|  \|  \|  \|  \|  \|  \| \|  \|  \|  \|  \|  \|  \|  \| \|  \|  \|  \|  \|  \|  \|  \| \|  \|  \|  \|  \|  \|  \|  \| \|  \|  \|  \|  \|  \|  \|  \| \|  \|  \|  \|  \|  \|  \|  \| \|  \|  \|  \|  \|  \|  \|  \| \|  \|  \|  \|  \|  \|  \|  \| \|  \|  \|  \|  \|  \|  \|  \| | \|  \|  \|  \|  \|  \|  \| \| --- \| --- \| --- \| --- \| --- \| --- \| \|  \|  \|  \|  \|  \|  \| \|  \|  \|  \| \|  \|  \| \|  \|  \|  \| \|  \|  \| \|  \|  \|  \|  \|  \|  \| \|  \|  \|  \|  \|  \|  \| \|  \|  \|  \|  \|  \|  \| \|  \|  \|  \|  \|  \|  \| \|  \|  \|  \|  \|  \|  \| \|  \|  \|  \|  \|  \|  \| \|  \|  \|  \|  \|  \|  \| \|  \|  \|  \|  \|  \|  \| \|  \|  \|  \|  \|  \|  \| \|  \|  \|  \|  \|  \|  \| \|  \|  \|  \|  \|  \|  \| \|  \|  \|  \|  \|  \|  \| \|  \|  \|  \|  \|  \|  \| \|  \|  \|  \|  \|  \|  \| \|  \|  \|  \|  \|  \|  \| \|  \|  \|  \|  \|  \|  \| \|  \|  \|  \|  \|  \|  \| \|  \|  \|  \|  \|  \|  \| \|  \|  \|  \|  \|  \|  \| \|  \|  \|  \|  \|  \|  \| \|  \|  \|  \|  \|  \|  \| \|  \|  \|  \|  \|  \|  \| \|  \|  \|  \|  \|  \|  \| \|  \|  \|  \|  \|  \|  \| \|  \|  \|  \|  \|  \|  \| \|  \|  \|  \|  \|  \|  \| \|  \|  \|  \|  \|  \|  \| \|  \|  \|  \|  \|  \|  \| \|  \|  \|  \|  \|  \|  \| \|  \|  \|  \|  \|  \|  \| \|  \|  \|  \|  \|  \|  \| \|  \|  \|  \|  \|  \|  \| \|  \|  \|  \|  \|  \|  \| |
| --- | --- | --- | --- | --- | --- | --- | --- | --- | --- | --- | --- | --- | --- | --- | --- | --- | --- | --- | --- | --- | --- | --- | --- | --- | --- | --- | --- | --- | --- | --- | --- | --- | --- | --- | --- | --- | --- | --- | --- | --- | --- | --- | --- | --- | --- | --- | --- | --- | --- | --- | --- | --- | --- | --- | --- | --- | --- | --- | --- | --- | --- | --- | --- | --- | --- | --- | --- | --- | --- | --- | --- | --- | --- | --- | --- | --- | --- | --- | --- | --- | --- | --- | --- | --- | --- | --- | --- | --- | --- | --- | --- | --- | --- | --- | --- | --- | --- | --- | --- | --- | --- | --- | --- | --- | --- | --- | --- | --- | --- | --- | --- | --- | --- | --- | --- | --- | --- | --- | --- | --- | --- | --- | --- | --- | --- | --- | --- | --- | --- | --- | --- | --- | --- | --- | --- | --- | --- | --- | --- | --- | --- | --- | --- | --- | --- | --- | --- | --- | --- | --- | --- | --- | --- | --- | --- | --- | --- | --- | --- | --- | --- | --- | --- | --- | --- | --- | --- | --- | --- | --- | --- | --- | --- | --- | --- | --- | --- | --- | --- | --- | --- | --- | --- | --- | --- | --- | --- | --- | --- | --- | --- | --- | --- | --- | --- | --- | --- | --- | --- | --- | --- | --- | --- | --- | --- | --- | --- | --- | --- | --- | --- | --- | --- | --- | --- | --- | --- | --- | --- | --- | --- | --- | --- | --- | --- | --- | --- | --- | --- | --- | --- | --- | --- | --- | --- | --- | --- | --- | --- | --- | --- | --- | --- | --- | --- | --- | --- | --- | --- | --- | --- | --- | --- | --- | --- | --- | --- | --- | --- | --- | --- | --- | --- | --- | --- | --- | --- | --- | --- | --- | --- | --- | --- | --- | --- | --- | --- | --- | --- | --- | --- | --- | --- | --- | --- | --- | --- | --- | --- | --- | --- | --- | --- | --- | --- | --- | --- | --- | --- | --- | --- | --- | --- | --- | --- | --- | --- | --- | --- | --- | --- | --- | --- | --- | --- | --- | --- | --- | --- | --- | --- | --- | --- | --- | --- | --- | --- | --- | --- | --- | --- | --- | --- | --- | --- | --- | --- | --- | --- | --- | --- | --- | --- | --- | --- | --- | --- | --- | --- | --- | --- | --- | --- | --- | --- | --- | --- | --- | --- | --- | --- | --- | --- | --- | --- | --- | --- | --- | --- | --- | --- | --- | --- | --- | --- | --- | --- | --- | --- | --- | --- | --- | --- | --- | --- | --- | --- | --- | --- | --- | --- | --- | --- | --- | --- | --- | --- | --- | --- | --- | --- | --- | --- | --- | --- | --- | --- | --- | --- | --- | --- | --- | --- | --- | --- | --- | --- | --- | --- | --- | --- | --- | --- | --- | --- | --- | --- | --- | --- | --- | --- | --- | --- | --- | --- | --- | --- | --- | --- | --- | --- | --- | --- | --- | --- | --- | --- | --- | --- | --- | --- | --- | --- | --- | --- | --- | --- | --- | --- | --- | --- | --- | --- | --- | --- | --- | --- | --- | --- | --- | --- | --- | --- | --- | --- | --- | --- | --- | --- | --- | --- | --- |


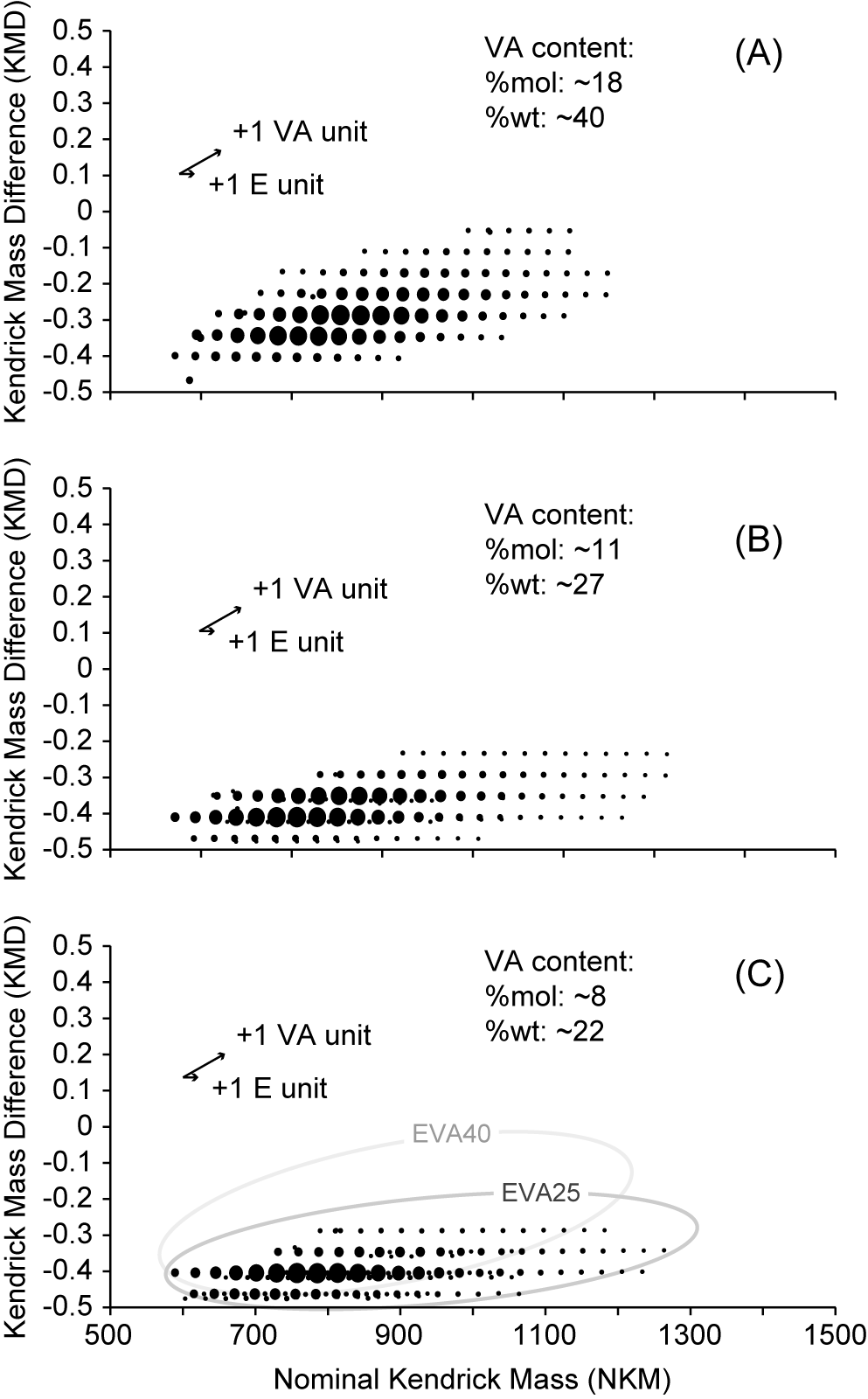


**Figure S5.** KMD plots from the fractions #3 of (A) EVA40, (B) EVA25 and (C) EVA18. VA content (mol% and wt%) calculated from the E_m_VA_n_ at the centroid is given in each case and found in good agreement with the values provided by the supplier for EVA40 and EVA25.

Approximations of the KMD plots for EVA40 and EVA25 are reprinted in (C) for sake of comparison (light grey and dark grey, resp.)


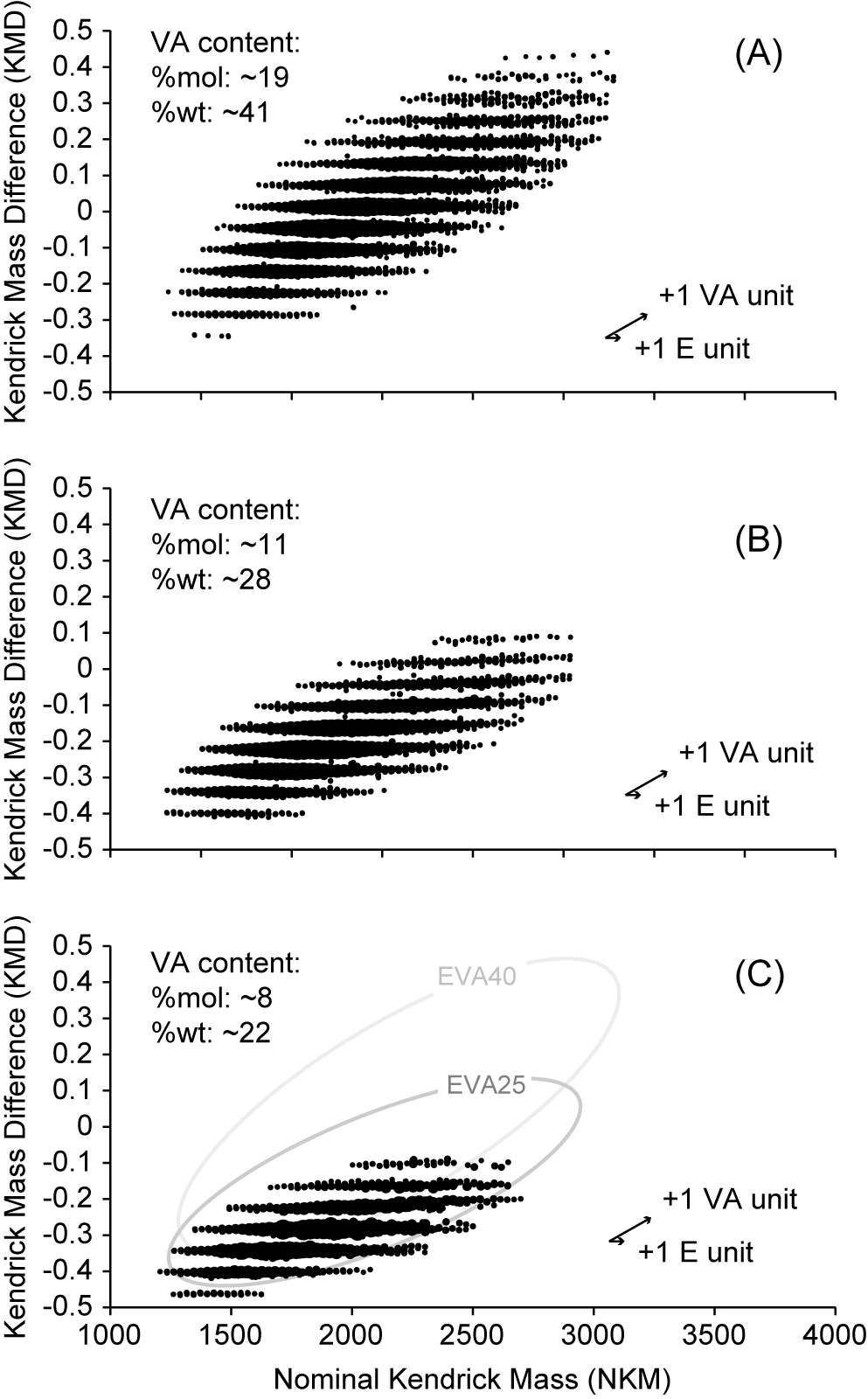


**Figure S6.** KMD plots from the fractions #1 of (A) EVA40, (B) EVA25 and (C) EVA18. VA content (mol% and wt%) calculated from the E_m_VA_n_ at the centroid is given in each case and found in good agreement with the values provided by the supplier for EVA40 and EVA25.

Approximations of the KMD plots for EVA40 and EVA25 are reprinted in (C) for sake of comparison (light grey and dark grey, resp.)


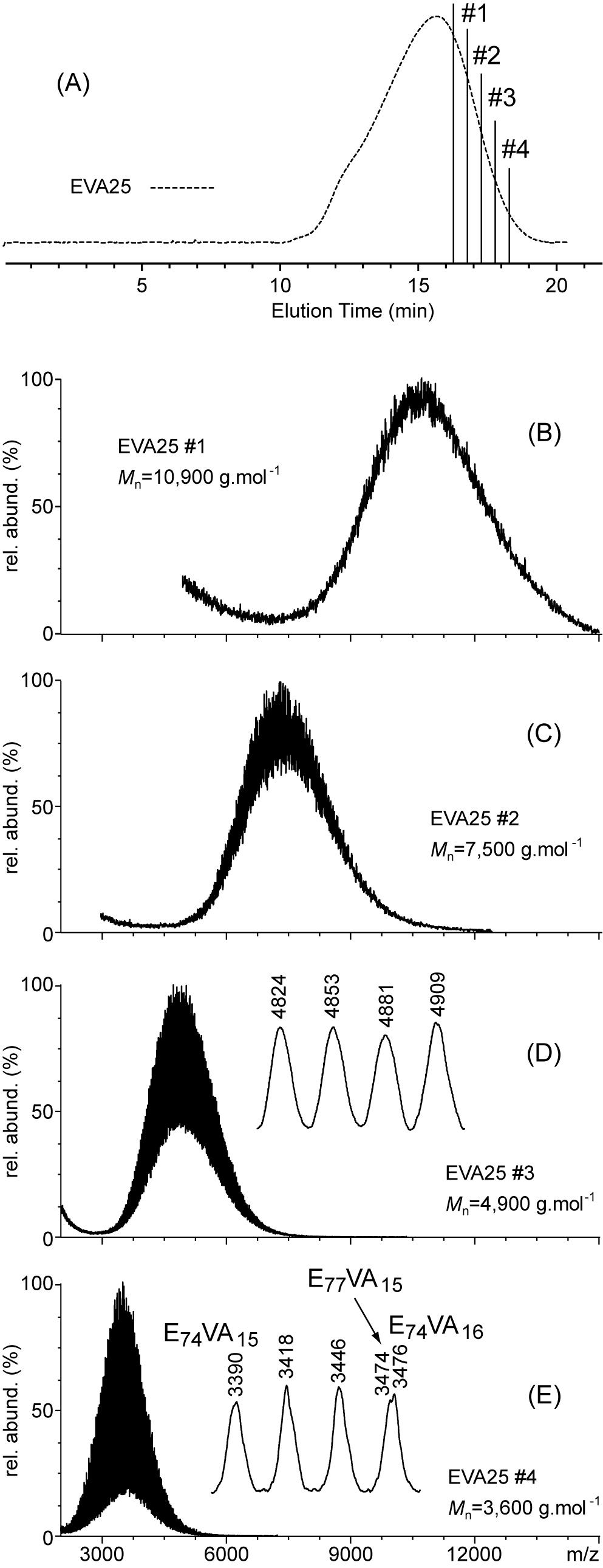


**Figure S7.** (A) SEC chromatogram of EVA25 and (B-E) MALDI mass spectra of the four collected fractions. Number average molecular weight *M*_n_ is mentioned for each spectrum.

The resolving power is still high enough for the last fraction #4 to allow the distinction of E_m+3_VA_n_ and E_m_VA_n+1_ species (spaced by 2 Da) but the isobaric issue of E_m+3_VA_n_(^13^C_2_) and E_m_VA_n+1_ can no longer be overcome.

No peak assignment could be proposed for the first three fractions owing to the lack of resolving power, preventing for the evaluation of the VA content. Molecular weight remains the only extractable information from those mass spectra.
